# Bone marrow B lymphopoiesis accelerates early cerebral amyloid pathology

**DOI:** 10.1038/s41392-025-02419-0

**Published:** 2025-09-18

**Authors:** Jing Zhang, Wenting Fang, Hanchen Liu, Ran Li, Zhibao Zhu, Xin Wu, Shaobo Yao, Ying Fu, Rui Li, Wanjin Chen, Qinyong Ye, Qiang Liu, Xiaochun Chen

**Affiliations:** 1 Department of Neurology, Fujian Medical University Union Hospital, Fujian Key Laboratory of Molecular Neurology and Institute of Neuroscience, Fujian Medical University, Fuzhou, China; 2 Department of Neurology, Tianjin Neurological Institute, Tianjin Medical University General Hospital, Tianjin, China; 3 Department of Neurology and Institute of Neurology, The First Affiliated Hospital of Fujian Medical University, Fuzhou, China

**Keywords:** Neurological disorders, Neuroimmunology

## Abstract

Bone marrow is a major source of hematogenous cells that orchestrate brain immunity. However, alterations in the bone marrow hematopoietic system in patients with Alzheimer’s disease (AD) and their potential impacts on neuroinflammation and cerebral β-amyloid (Aβ) pathology remain unknown. Here, we report that Aβ accumulates within the bone marrow of patients with AD and is particularly concentrated in the central nervous system-surrounding bones. In 5 × FAD and APP/PS1 mice, two classic mouse AD models, Aβ accumulates within the skull bone marrow prior to substantial cerebral Aβ deposits. Flow cytometry and cell tracking analyses demonstrated that these AD mice exhibit enhanced bone marrow hematopoiesis in B lymphoid lineages, specifically an increase in age-associated B cells (ABCs), accompanied by heightened output of these cells into the brain parenchyma. Furthermore, intracranial Aβ injection into IL-6 knockout mice revealed that Aβ promotes B lymphocyte generation, particularly ABCs, via IL-6 signaling. Single-cell sequencing analysis following intracerebroventricular ABCs injection, combined with in vitro microglial culture studies, demonstrated that bone marrow-derived ABCs directly augment microglial reactivity, ultimately exacerbating Aβ neuropathology and cognitive deficits in AD models. Notably, blockade of IL-6R restricts B-cell activity and ABCs in the bone marrow, delays cerebral Aβ pathology and improves cognition. Our findings reveal the potential involvement of bone marrow-derived B cells in the early cerebral amyloid pathology in two mouse AD models and suggest that these B cells may serve as potential therapeutic candidates for patients with AD.

## Introduction

Amyloid plaques feature prominently in Alzheimer’s disease (AD). As an essential indicator of AD diagnosis, Aβ starts to aggregate insidiously about 20 years before the presence of any obvious symptoms.^1^ During this long interval, neuroinflammation is a key driving factor for the AD progression,^2^ which can be exacerbated by nearly half of the AD risk genes (APOE, TREM2, CD33, etc.) that are implicated in immune processes,^3,4^ increased levels of complement molecules and inflammatory mediators,^5^ and the reactive gliosis and infiltration of immune cells in the brain. Recent studies document microglia as the primary contributors to neuroinflammation in the AD brain, and suggest that their activation status regulates the pathological progression of AD.^5,6^ In addition to the resident microglia in the brain,^7–10^ the peripheral immune system, encompassing innate and acquired immune cells, has been found to be involved in the AD pathogenesis.^11,12^

Previous studies have revealed the detrimental effects of peripheral-derived myeloid cells (e.g., neutrophils and monocytes) on the AD progression_._^13,14^ Recent studies reported an increase of T cells in AD mouse models^15^ and in the leptomeninges, CSF, and hippocampus of patients with AD.^16^ Moreover, T cell infiltration in AD brains is linked with tau pathology, but not with amyloid pathology, and tau aggregation is considered to be an advanced stage of AD progression.^17–19^ Wei et al. explored the beneficial role of CD8^+^ T cells in AD mice.^20^ The involvement of B cells in AD has been reported less extensively than other immune components. Patients with AD demonstrate elevated systemic autoantibodies and an increased population of antibody-secreting B cells in peripheral blood.^21^ Matteo et al. documented that individuals with moderate-to-severe AD report a decrease in peripheral CD19^+^ B cell count and a remodeling of B cell subsets. This remodeling is characterized by an increase of double-negative (IgD^-^CD27^-^) memory B cells, a marked reduction in naïve B cells (IgD^+^ CD27^−^), and an upregulation of receptors for pro-inflammatory cytokines.^22,23^ Within the AD brain, B lymphocytes are found to be adjacent to Aβ plaques^24^ and IgG-immunopositive neurons.^25^ Although the above evidence suggests that the peripheral immune system is implicated in AD pathogenesis, much remains to be desired with regards to the origin and types of peripheral immune cells and the relevant mechanisms in the early stage of AD.

Under physiological conditions, the integrity of the brain barrier system—encompassing the meninges, blood-cerebrospinal fluid barrier, and blood-brain barrier (BBB)—limits the presence of peripheral immune cells within the brain parenchyma to very low levels, allowing their participation in brain development and functional homeostasis. Conversely, in the late stages of AD, vascular inflammation, BBB dysfunction, and impaired meningeal lymphatic drainage collectively facilitate substantial infiltration of peripheral immune cells. However, it remains unresolved regarding the origin of peripheral immune cells and the operative mechanism by which they enter the brain parenchyma during the early stages of AD. As a critical immune organ within the peripheral compartment, the bone marrow (BM) is the major site of hematopoiesis, where hematopoietic stem and progenitor cells (HSPCs) produce immune cells that orchestrate immune homeostasis in both the periphery and the central nervous system (CNS).^26–32^ Recent evidence has shown that there is a direct short-vessel connection between the cranial bone marrow and the meninges, through which the skull BM supplies myeloid cells and B cells to the meninges and CNS parenchyma to monitor brain homeostasis.^26–29^ Utilizing TSPO-PET imaging, Kolabas et al. detected elevated myeloid cell activation within the skull bone marrow of AD patients.^27^ Consequently, the skull bone marrow serves as a primary source of brain-surveilling immune cells, reflecting neuroinflammatory responses in various neurological disorders. Although few studies have reported the presence of abnormal bone marrow hematopoiesis in AD model mice,^30,33,34^ several key questions need to be answered in order to understand how bone marrow in the peripheral compartment communicates with the brain to orchestrate neuroinflammation.

In this study, we uncover skull bone marrow-derived B-cell that accelerates early AD pathogenesis. We detected Aβ deposition in the skull BM of AD patients and young AD model mice. Aβ promotes the differentiation and proliferation of HSCs and B lymphocytes, especially age-associated B cells (ABCs), in the bone marrow via IL-6. Notably, in vivo tracing of bone marrow cells revealed that ABCs from skull BM can infiltrate the brain parenchyma and lead to accelerated Aβ deposition. Furthermore, blocking IL-6R suppresses B cells and ABCs in the bone marrow, delays cerebral Aβ pathology and improves cognition. These findings suggest that bone marrow-derived B cells are potential therapeutic targets for patients with AD.

## Results

### Aβ accumulates in the bone marrow of patients with AD and AD model mice

**Fig. 1:**
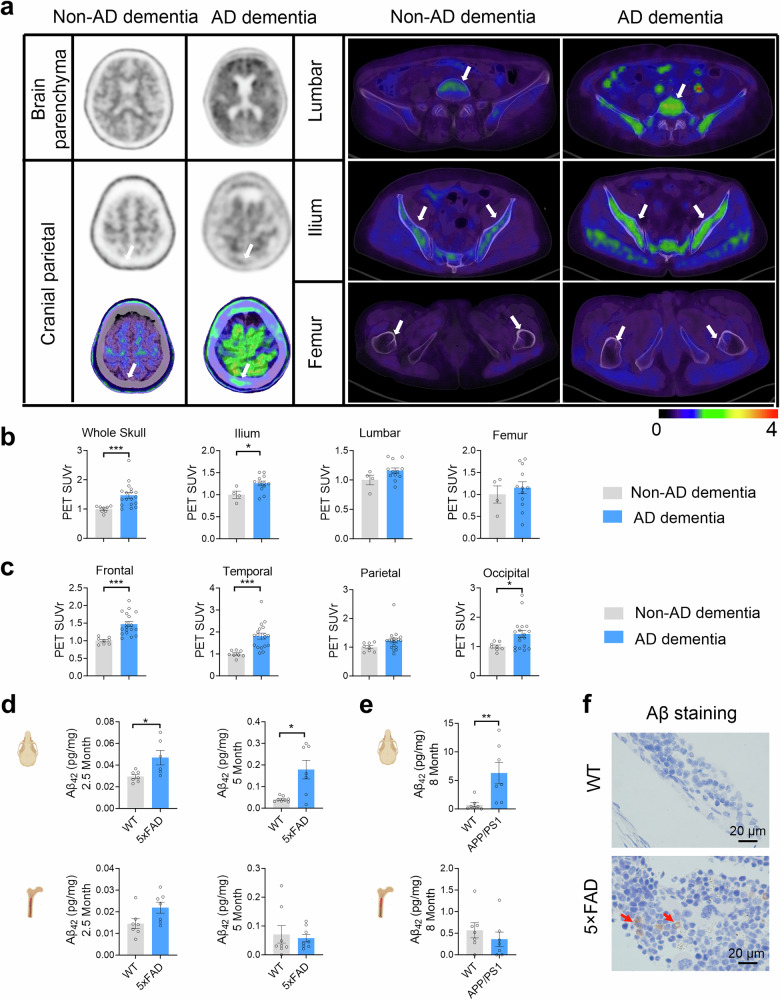
Aβ accumulates in the skull BM of patients with AD and AD model mice. **a** ^18^F-92 and ^18^F-AV45 PET/CT scanning analysis of Aβ deposition in the brain and bone marrow. Images demonstrating the uptake of the PET tracers in the body bone marrow of the non-AD dementia group and AD dementia group. The white arrows indicate the cranial parietal spine, lumbar spine, iliac crest and femur. **b**, **c** Quantitative measurement standardized uptake values of the skull, ilium, lumbar, and femur (**b**) and subregions of the skull (**c**) (SUVr). n = 8 and 18 skulls and subregions of the skull for non-AD dementia patients and AD dementia patients, respectively. *n* = 4 and 12 ilium, lumbar, and femur for non-AD dementias and AD dementias patients, respectively. **d**, **e** Hypersensitive ELISA analysis of Aβ_42_ in the supernatant of skull and femur bone marrow from 5 × FAD mice (**d**), APP/PS1 mice (**e**) and littermates of WT counterparts. *n* = 6–8 mice per group. **f** Immunohistochemical staining showing Aβ deposition in the skull bone marrow of 5-month-old WT (top) and 5 × FAD (bottom) mice. The data are expressed as the means ± SEMs. *p < 0.05, **p < 0.01; two-tailed unpaired Student’s t test was used

The aggregation of Aβ in the brain is a major neuropathologic hallmark of AD, but whether Aβ deposits in peripheral immune organs such as the bone marrow remains unknown in patients with AD. For this purpose, Aβ accumulation was assessed via the regional cortical tracer uptake (RCTU) system by positron emission tomography/computed tomography (PET/CT).^35,36^ A total of 26 dementia patients were included, comprising 8 non-AD dementia patients with negative brain Aβ-PET scans and 18 AD dementia patients with positive brain Aβ-PET scans (supplementary Table 1). In addition to Aβ deposition in the brain parenchyma, Aβ accumulation in the skull bones and other peripheral bones was detected in patients with AD dementia compared with non-AD dementia patients (Fig. 1a). Analysis of tracer uptake revealed that the standardized uptake value ratio (SUVr) in the skull of patients with AD dementia was markedly greater than that in non-AD dementia patients (Fig. 1b). Interestingly, Aβ deposits were also found in the bone marrow of flat bones, such as the ilium, in AD dementia patients but not in the bone marrow of long bones (e.g., the femur) in any patient group. When SUV values in skull subregions were analyzed according to corresponding cerebral cortex regions, significant increases were found in the frontal, temporal, and occipital skulls of patients with AD dementia compared with those with non-AD dementia, whereas the parietal skull showed no significant difference (Fig. 1c). These results demonstrate that Aβ is deposited within the skull bone marrow of AD dementia patients.

To test whether Aβ deposits in the bone marrow also exist in mouse models of AD, we quantified Aβ_42_ levels in the skull and femur BM of 2.5-month-old and 5-month-old 5 × FAD mice via a high-sensitivity ELISA kit. We found that the level of Aβ_42_ in the skull BM supernatant was higher in 2.5-month-old 5 × FAD mice than in wild-type (WT) mice and increased further in the skull BM supernatant at the age of 5 months (Fig. 1d). In contrast, no marked increase in Aβ_42_ was evident in the femoral BM of 5 × FAD mice. Similarly, Aβ_42_ was deposited in the skull bone marrow of 8-month-old APP/PS1 mice but not in the femoral bone marrow (Fig. 1e). The immunohistochemical results also revealed Aβ accumulation in the skull BM of 5-month-old 5×FAD mice (Fig. 1f). These results demonstrate that Aβ deposition occurs in the skull BM prior to massive plaque accumulation within the brain parenchyma during the early AD stage.

### Proliferation and differentiation of skull bone marrow HSCs and B lymphopoiesis in the early AD stage

**Fig. 2:**
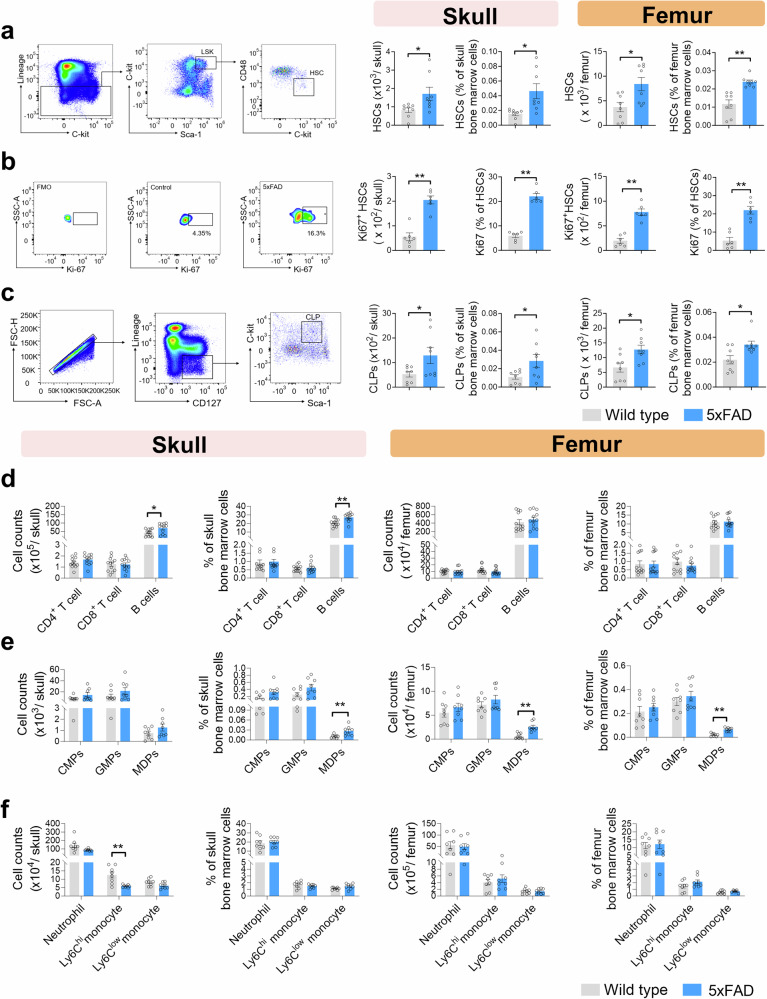
Proliferation and differentiation of HSCs and increased lymphopoiesis in the skull BM of 3-month-old 5×FAD mice. Flow cytometry detection of bone marrow HSCs, immune progenitor cells and mature immune cells in 3-month-old 5 × FAD mice and WT mice. **a** Gating strategy for HSCs in the BM (Lin^-^ c-kit^+^ sca-1^+^ CD34^−^ FLK2^−^ CD48^−^ CD150^+^) (left), counts and frequency of HSCs in the skull and femur BM (right). n = 8 mice per group for skulls or femurs. **b** Gating strategy for HSCs with Ki67^+^ cells in the BM (Ki67^+^) (left), counts and frequency of Ki67^+^ HSCs in the skull and femur BM (right). *n* = 6 mice per group for skulls or femurs. **c** Gating strategy of common lymphoid progenitors (CLPs, Lin^−^ CD127^+^ Sca-1^int^ c-Kit^int^) in the bone marrow (left), counts and frequency of CLPs in the skull and femur BM (right). *n* = 8 mice per group for skulls or femurs. **d** Counts and frequencies of B cells (CD45^+^ CD3^-^ CD19^+^), CD4 + T cells (CD45^+^ CD3^+^ CD4^+^) and CD8 + T cells (CD45^+^ CD3^+^ CD8^+^) in the skull (left) and femur BM (right). *n* = 12 mice per group for skulls or femurs. The gating strategy is depicted in supplementary Fig. 1a. **e** Counts and frequency of common myeloid progenitors (CMPs, Lin^−^ Sca-1^−^ c-Kit^+^ CD34^+^ CD16/32^int^), granulocyte-monocyte progenitors (GMPs, Lin^−^ Sca-1^−^ c-Kit^+^ CD34^+^ CD16/32^hi^) and monocyte-dendritic cell progenitors (MDPs, Lin^−^ Sca-1^−^ c-Kit^+^ CD34^+^ CD16/32^hi^ CD115^+^) in the skull (left) and femur BM (right). The gating strategy is depicted in supplementary Fig. 1b. *n* = 8 mice per group for skulls or femurs. **f** Counts and frequency of neutrophils (CD45^+^ CD11b^+^ Ly6G^+^), Ly6C^high^ monocytes (CD45^+^ CD11b^+^ Ly6G^−^ F4/80^−^ Ly6C^high^), and Ly6C^low^ monocytes (CD45^+^ CD11b^+^ Ly6G^−^ F4/80^-^ Ly6C^low^) in the skull (left) and femur BM (right). The gating strategy is depicted in supplementary Fig. 1c. *n* = 8 mice per group for skulls or femurs. The data are expressed as the means ± SEMs. *p < 0.05, **p < 0.01; two-tailed unpaired Student’s t test was used

To assess the potential alterations in bone marrow hematopoietic cell lineages in AD, we performed flow cytometric analysis of bone marrow HSCs, immune progenitor cells and mature immune cells from 3-month-old 5 × FAD mice and WT mice. The results showed a remarkable increase of long-term HSCs (Lin^-^ c-kit^+^ sca-1^+^ CD34^-^ FLK2^-^ CD48^-^ CD150^+^) in the skull BM (Fig. 2a), Ki67^+^ HSCs in 5 × FAD mice (Fig. 2b), and skull BM lymphoid progenitor cells (CLPs) (Fig. 2c). Notably, there was a robust increase of B lymphocytes, not CD4^+^ T cells or CD8^+^ T cells, in the skull BM (Fig. 2d, supplementary Fig. 1a). Whereas, the number of monocyte‒dendritic progenitor (MDP) cells, common myeloid progenitors (CMPs), and granulocyte–monocyte progenitors (GMPs) was not significantly altered (Fig. 2e, supplementary Fig. 1b). Moreover, except for Ly6C^high^ monocytes, no apparent discrepancy in the number of neutrophils or Ly6C^low^ monocytes was evident between 5 × FAD mice and WT mice (Fig. 2f, supplementary Fig. 1c). In addition, we detected increases in HSCs, Ki67^+^ HSCs, CLPs and MDPs within femur BM (Fig. 2a–c, e). However, there were few changes in the number of other immune progenitor cells and mature immune cells within the femur BM (Fig. 2d, f). Overall, in 3-month-old 5 × FAD mice, we observed an increase of both HSC counts and activity in the skull and femur BM but only an increase of B lymphopoiesis in the skull BM. In 8-month-old APP/PS1 mice, both HSCs and proliferative HSCs were also elevated in the BM (supplementary Fig. 2a, b and 2e, f), with a marked increase of CLPs only in the skull BM (supplementary Fig. 2c, g).

To further characterize the dynamic alterations in the bone marrow hematopoietic system, we also performed flow cytometry analysis of bone marrow from 10-month-old 5 × FAD mice and WT mice. We found a sustained increase in HSC count and activity (supplementary Fig. 3a, b), together with a dramatic increase in B lymphopoiesis in both skull BM and femur BM (supplementary Fig. 3c, d). These results highlight an enhanced differentiation of HSCs to the lymphoid lineage, particularly B cells in the BM of AD mice, early only in the skull BM and late in the femoral BM.

### Bone marrow-derived B lymphocytes infiltrate the brain parenchyma in the early AD stage

**Fig. 3:**
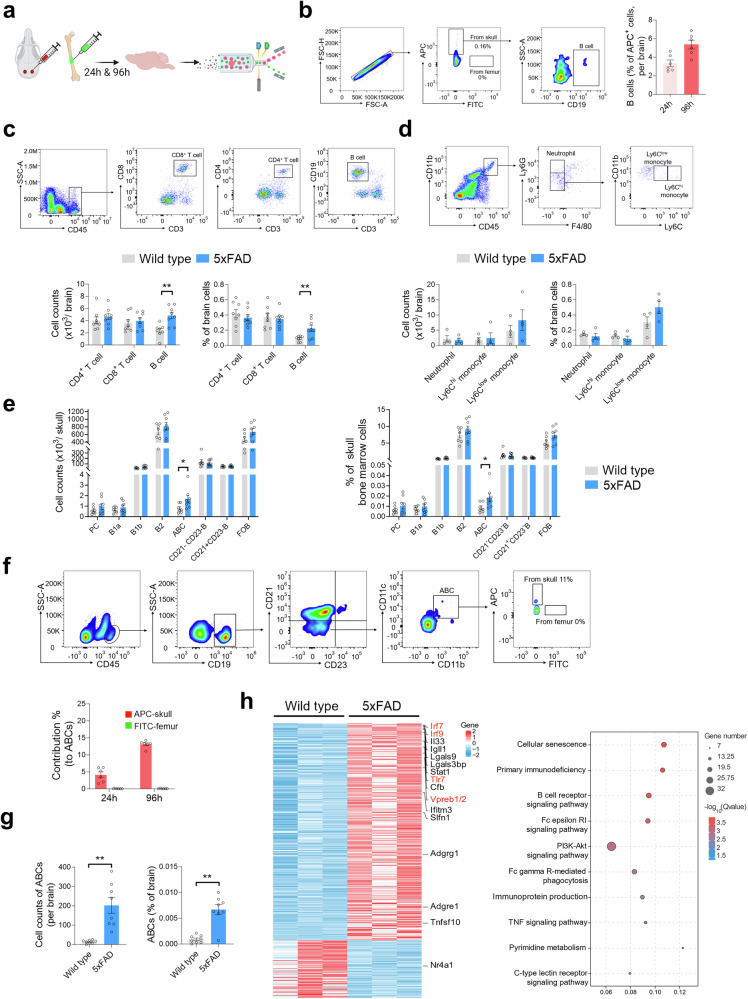
Skull bone marrow-derived B cells, particularly ABCs, invade the brain parenchyma of 3-month-old 5 × FAD mice. **a**, **b** Schematic diagram depicting dye tracing of bone marrow (**a**). APC^+^ tracers and FITC^+^ tracers were injected into the cavities of the skulls and femurs of 3-month-old 5 × FAD mice, respectively, and flow cytometry of the brain was performed at 24 h and 96 h. Gating strategy for APC^+^ B cells (CD45^+^ CD19^+^) (left), frequency of APC^+^ B cells in APC^+^ cells in brain tissue (right) (**b**). *n* = 6 5 × FAD mice. **c** Flow cytometry analysis of lymphocytes in the brain tissue of 3-month-old 5 × FAD mice and WT counterparts. Gating strategy for lymphocytes in brain tissue (top). The counts and frequencies of B cells, CD4^+^ T cells and CD8^+^ T cells (bottom). *n* = 8 mice per group. **d** Flow cytometry analysis of myeloid cells in the brain tissue of 3-month-old 5 × FAD mice and WT counterparts. Gating strategy for myeloid cells in brain tissue (top). The counts and frequency of neutrophils (CD45^high^ CD11b^+^ Ly6G^+^), Ly6C^high^ monocytes (CD45^high^ CD11b^+^ Ly6G^−^ F4/80^−^ Ly6C ^high^) and Ly6C^low^ monocytes (CD45^high^ CD11b^+^ Ly6G^−^ F4/80^−^ Ly6C ^low^) (bottom). *n* = 4 mice per group. **e** Flow cytometry analysis of B-cell subpopulations in the skull BM of 3-month-old 5 × FAD mice and WT counterparts. *n* = 7–8 mice per group. Counts and frequency of plasma cells (PCs) (CD45^+^ CD3^−^ CD19^+^CD138^+^), B1a (CD45^+^ CD3^−^ CD19^+^ CD11b^+^CD5^+^), B1b (CD45^+^ CD3^−^ CD19^+^ CD11b^+^CD5^−^), B2 (CD45^+^ CD3^-^ CD19^+^CD11b^−^CD5^−^), ABCs (CD45^+^ CD19^+^ CD11b^+^ CD11c^+^ CD21^−^ CD23^-^), CD21^-^CD23^-^B cells (CD45^+^ CD19^+^ CD21^−^CD23^−^), CD21^+^CD23^−^B cells (CD45^+^ CD19^+^ CD21^+^CD23^−^), and FOB (CD45^+^ CD19^+^ CD21^+^CD23^+^). The gating strategy is depicted in supplementary Fig. 1**d. f** Dye-tracing of bone marrow analysis of ABC output. Gating strategy for APC^+^ and FITC^+^ ABCs (top). The histogram represents the frequency of APC^+^ and FITC^+^ ABCs in brain tissue (bottom). *n* = 6 5 × FAD mice. **g** Flow cytometry analysis of ABCs in the brain tissue of 3-month-old 5 × FAD mice and WT counterparts. *n* = 8 mice per group. **h** Bulk RNA sequencing analysis of bone marrow-derived ABCs from 5 × FAD mice and WT counterparts. *n* = 3 mice per group. Heatmap showing the DEGs (fold change>2.0; adj. FDR value < 0.05) between 5 × FAD mice and WT counterparts; representative genes are listed on the right (left). Plot showing the top pathways enriched with the DEGs according to KEGG analysis (right). The data are expressed as the means ± SEMs. *p < 0.05 and **p < 0.01; two-tailed unpaired Student’s t test was used

To test whether B cells from skull BM infiltrate the brain parenchyma in 3-month-old 5 × FAD mice, we injected APC- or FITC-labeled tracers into the cavities of the skull and femur, respectively, to trace skull- or femur-derived cells by flow cytometry 24 h or 96 h after injection (Fig. 3a). Consistent with our speculation, the results showed the presence of APC-positive B cells in the brain parenchyma of 3-month-old 5 × FAD mice at both 24 h and 96 h. Importantly, there were no FITC-positive B cells in the brain parenchyma at any time point (Fig. 3b). These results evidence that newly generated B cells can be mobilized from skull BM and penetrate the brain parenchyma.

Next, we further investigated whether the production of B cells from the skull BM increases B cells in the brain. We calculated the number of peripheral immune cells in the brain parenchyma of young AD mice. Indeed, there was an increase of B cells in the brain tissue of 3-month-old 5 × FAD mice (Fig. 3c), but no marked changes in the number of CD8^+^ T cells, CD4^+^ T cells, neutrophils, Ly6C^high^ monocytes, or Ly6C^low^ monocytes, between 5 × FAD mice and WT counterparts (Fig. 3d). Similarly, an increase of B cells was predominant in the brains of 8-month-old APP/PS1 mice (supplementary Fig. 2h). Notably, we detected a sustained and dramatic increase in B cells in the brains of 10-month-old 5 × FAD mice (supplementary Fig. 4a). In addition, we detected similar numbers of B cells in the spleens of 3-month-old (supplementary Fig. 4c) and 10-month-old (supplementary Fig. 4d) 5 × FAD and WT mice. These data demonstrate sustained B-cell output from the bone marrow to the brain parenchyma in AD mice, indicating the contribution of these cells to Aβ pathology.

### Bone marrow-derived age-associated B cells infiltrate the brain parenchyma in the early AD stage

Furthermore, we analyzed the major subclusters of B cells in the skull BM via flow cytometry. Compared with littermate WT mice, 3-month-old 5 × FAD mice presented a significantly greater frequency and number of ABCs (CD45^+^ CD19^+^ CD11b^+^ CD11c^+^ CD21^−^ CD23^−^) and no significant differences in CD138^+^B, B1a (CD11b^+^ CD5^+^), B1b (CD11b^+^ CD5^−^), B2 (CD11b^−^ CD5^−^), CD21^−^ CD23^−^ B, CD21^+^ CD23^−^ B and FOB (CD21^+^ CD23^+^) (Fig. 3e) (supplementary Fig. 1d). Consistently, an increase in ABCs was also present in the skull BM of 8-month-old APP/PS1 mice (supplementary Fig. 2d). Bone marrow tracing further revealed that skull BM-derived ABCs could be exported to the brain parenchyma in 3-month-old 5 × FAD mice at either 24 h or 96 h (Fig. 3f). Furthermore, the number and percentage of ABCs in the brain parenchyma of both 3-month-old 5 × FAD mice (Fig. 3g) and 8-month-old APP/PS1 mice (supplementary Fig. 2j) were significantly increased, as determined via flow cytometry.

Next, consistent with the previous report,^37^ we also found that ABCs highly express the transcription factor Zeb2 (supplementary Fig. 5a, b). Immunofluorescence staining revealed that ABCs were predominantly located in the perivascular and periventricular regions of the brain parenchyma in 5 × FAD mice (supplementary Fig. 5c). To characterize the features of ABCs, we further performed bulk RNA-sequencing analysis of bone marrow-derived ABCs (Fig. 3h). Differential gene expression analysis between WT and 5 × FAD (fold change > 2.0; FDR < 0.05) revealed 410 differentially expressed genes. Notably, compared with WT mice, the 5 × FAD mice reported an increase in TLR7, IRF7, IRF9, and Vpreb1/2, which were previously reported to be associated with ABC production,^38^ and IL33, Lgals9, Cfb, and Tnfsf10, which are reportedly associated with the dysfunction of myeloid cells.^39,40^ However, Nr4a1, the gatekeeper of B-cell immune tolerance, was decreased in 5 × FAD mice.^41^ Pathway analysis confirmed that cell senescence, primary immunodeficiency, the B-cell receptor signaling pathway and immunoprotein production were among the highly impacted pathways in the context of AD. These results suggest that the differentiation and output to the brain parenchyma of the skull BM B lymphoid lineage, especially ABCs, are increased in the early AD stage.

### Aβ deposition increases the number of age-associated B cells in the BM via IL-6

**Fig. 4:**
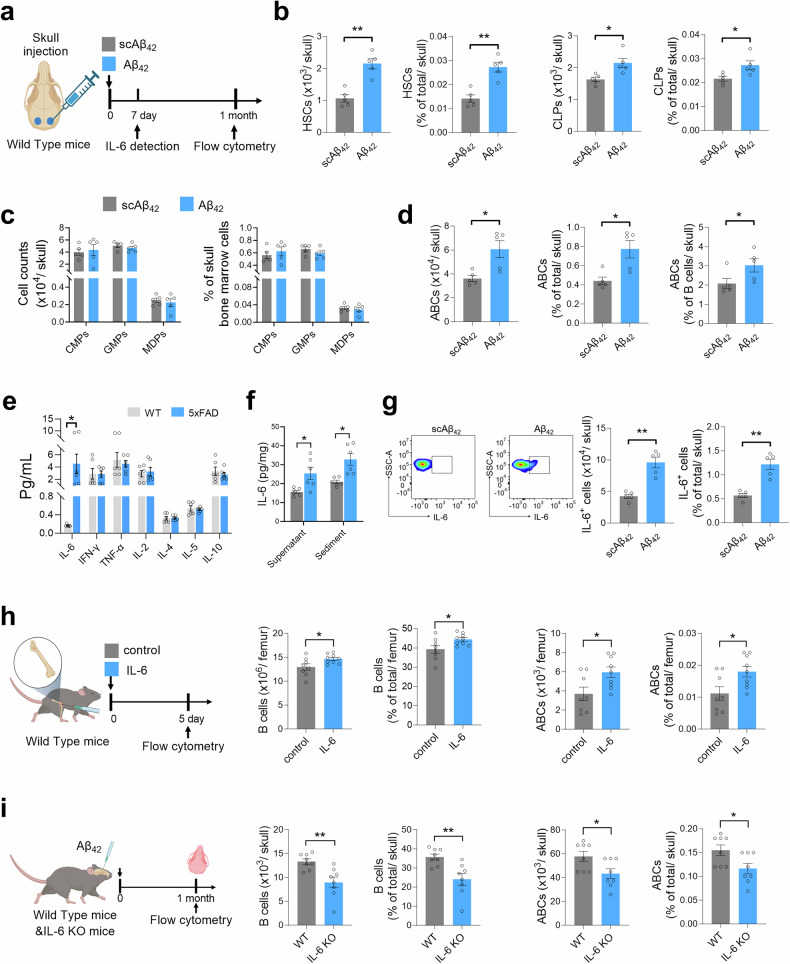
Aβ enhances skull bone marrow B lymphopoiesis and ABCs via IL-6. **a** Schematic diagram depicting the Aβ_42_ injection experiment in the skull cavities of 2.5-month-old WT counterparts. The outer periosteal layer of the skull bone was thinned near the BM sites, and 6 μl of Aβ_42_ (200 ng/μl) or scrambled Aβ_42_ (scAβ_42_) was applied to the two sites. After 7 days, ELISA for IL-6 was performed, followed by flow cytometry for the skull BM. **b–d** Counts and frequency of HSCs, CLPs (**b**), CMPs, GMPs, MDPs (**c**) and ABCs (**d**) in skull bone marrow. *n* = 5 WT mice per group. **e** Bar graph showing the cytokine expression profiles of the skull bone marrow of 3-month-old 5×FAD mice and WT mice. n = 6 mice per group. **f** ELISA analysis of IL-6 levels in the supernatant and cell pellet from skull bone marrow injected with Aβ_42_ or scAβ_42_. *n* = 6 WT mice per group. **g** Flow cytometry analysisof IL-6-positive cells in skull bone marrow. *n* = 5 WT mice per group. **h** Schematic diagram of the experiment in which the IL-6 protein was injected into the femur cavity of 2.5-month-old WT mice (left). Two microliters of IL-6 (100 ng/μl) or control (PBS) were applied to the bilateral femur after 5 days, followed by flow cytometry of the femur BM. Counts and frequency of B cells and ABCs in femur BM (right). *n* = 8 WT mice per group. **i** Schematic diagram depicting the Aβ_42_ injection experiment in the skull cavities of WT mice and IL-6 KO mice. The outer periosteal layer of the skull bone was thinned near the BM sites, and 6 μl of Aβ_42_ (200 ng/μl) was applied to the two sites after 1 month, followed by flow cytometry of the skull BM. Counts and frequency of B cells and ABCs in femur BM (right). *n* = 8 mice per group. The data are expressed as the means ± SEMs. *p < 0.05 and **p < 0.01; two-tailed unpaired Student’s t test was used

Since Aβ is deposited in the skull cavity both in patients with AD and in AD model mice, we hypothesized that Aβ directly promotes the proliferation and differentiation of HSCs and increases B lymphopoiesis and ABC production in AD mice. To address this issue, Aβ_42_ or scrambled Aβ_42_ was first injected into the skull cavities of WT mice and then the skull BM underwent the flow cytometric analysis at 1 month after injection (Fig. 4a). Surprisingly, Aβ_42_ injection significantly increased the number and frequency of HSCs, CLPs (Fig. 4b), and ABCs (Fig. 4d). However, the counts and frequencies of CMPs, GMPs, and MDPs were not significantly altered (Fig. 4c). Second, to determine the underlying mechanisms of increased B lymphopoiesis and ABC production in the skull BM of AD mice, we quantified immune factors from the skull BM and found that IL-6 level was greatly increased in 3-month-old 5×FAD mice (Fig. 4e) while that of other cytokines, such as IL-2, IL-4, IL-5, IL-10, TNF-α and IFN-γ, did not differ. Consistently, Aβ_42_ injection increased not only IL-6 level in the supernatants and precipitates of skull BM (Fig. 4f) but also the number and frequency of IL-6-positive cells in the skull BM of WT mice after 7 days of injection (Fig. 4a, g). These results indicate that Aβ not only increases IL-6 levels but also directly promotes the proliferation and differentiation of HSCs, the differentiation of B lymphocytes and the upregulation of ABCs in the bone marrow.

To determine the full and essential role of IL-6 in Aβ-mediated B lymphopoiesis and ABC production, first, IL-6 was injected into the BM cavity of WT mice, and flow cytometric analysis revealed that IL-6 increased the number and frequency of B cells and ABCs (Fig. 4h). Furthermore, Aβ_42_ was injected into the BM cavities of WT mice and IL-6 knockout mice and then the BM underwent flow cytometry at 1 month after injection. The results revealed that the number and frequency of B cells and ABCs were markedly lower in IL-6 KO mice than in WT counterparts (Fig. 4i). These data evidence that Aβ contributes to the differentiation and proliferation of B lymphocytes and the increase of ABCs in skull BM through IL-6 in the early AD stage.

### Bone marrow-derived ABCs accelerate early Aβ pathology and augment microglial reactivity in 5×FAD mice

**Fig. 5:**
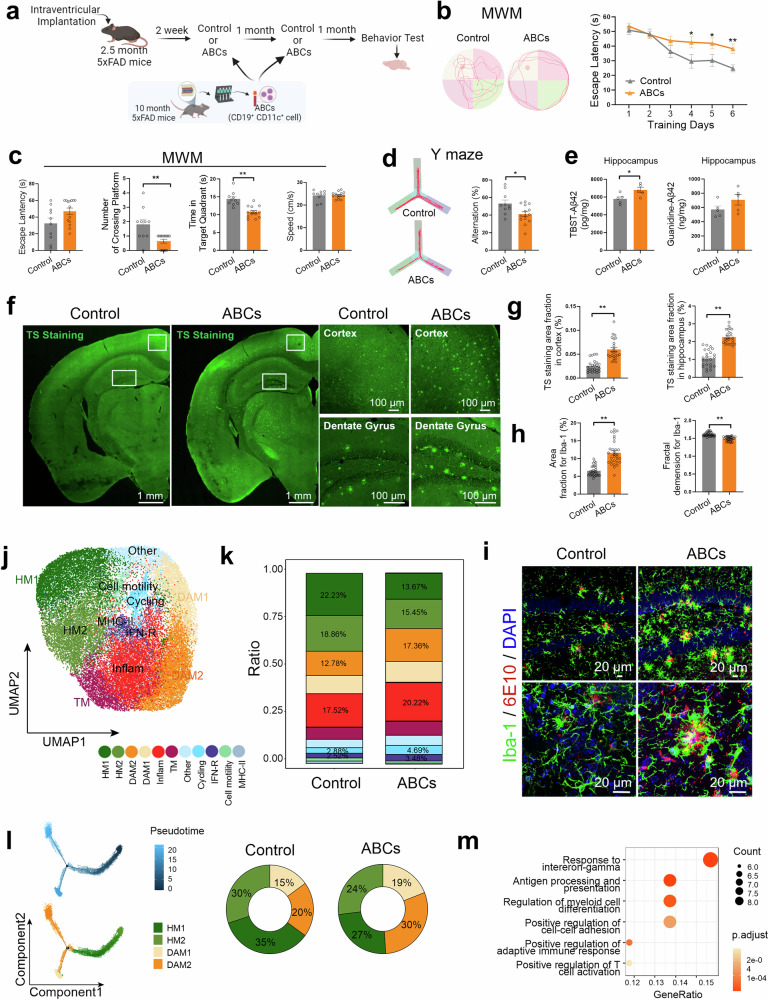
Bone marrow-derived ABCs augment Aβ pathology, microglial reactivity and cognitive impairment. **a** Schematic diagram of lateral ventricle injection of ABCs in 5×FAD mice. 5×FAD mice aged 2.5 months were intubated in the lateral ventricle and allowed to recover for 2 weeks, and bone marrow-derived ABCs were injected into the bilateral ventricles once a month until 5 months of age. Then, behavioral tests and pathology were performed. For the behavioral tests, *n* = 10 and 14 5×FAD-control and 5×FAD-ABCs, respectively, were used. CD19^+^ CD11c^+^ B cells derived from the BM of WT counterparts were used as controls. **b–d** Behavioral tests with the Morris water maze (MWM) and Y maze. The MWM test was used to assess spatial memory. Representative track images of the mice in the probe trial of the MWM test and escape latency to the platform during the training trials (**b**). Latency to first locate the target, number of target crossings, time spent in the target quadrant, and mean swimming speed of the mice in the probe trial of the MWM (**c**). Representative track images and correct spontaneous alternation rates in the Y maze (**d**). **e** Quantification of TBST-soluble Aβ_42_ (top) and guanidine-soluble Aβ_42_ (bottom) in the hippocampus via ELISA; *n* = 5 mice per group. **f–g** Representative images of TS staining of brain sections (left) and enlarged images of the cortex and hippocampal DG region (right) (**f**). Quantification of the TS-positive Aβ plaque area in the cortex and hippocampal DG region (right), n = 24 views from 3 mice per group (**g**). **h–i** Quantification of the Iba1-positive area (up) and Iba1-positive fractal dimension (down) in the hippocampus (**h**), *n* = 30 views from 3 mice per group for the area fraction, n = 36 views from 3 mice per group for the fractal dimension. Representative images of Aβ (red) and Iba1 (green) staining in the hippocampal DG region (top) and enlarged images (bottom) (**i**). **j–m** scRNA-seq was used to assess microglial reactivity. UMAP for 33394 microglia from *n* = 2 per group and annotated by cluster (**j**). Stacked bar plots showing the proportions of microglia in the two experimental groups (**k**). The pseudotime trajectory shows the trajectory of HM differentiation into DAM1 or DAM2 (left). Stacked bar plots showing the proportions of microglial states in the two experimental groups (right) (**i**). GO term analysis of DEGs associated with DAM in the ABC group compared with the control group (**m**). The data are presented as the means ± SEMs. *p < 0.05, **p < 0.01; two-tailed unpaired Student’s t test was used, except for the multiway repeated-measures ANOVA in (**b**)

To test the impact of ABC infiltration into the brain parenchyma on Aβ pathology, we sorted bone marrow-derived ABCs (CD19^+^ CD11c^+^) from 10-month-old 5×FAD mice and injected them into the bilateral ventricles of 3-month-old 5×FAD mice once a month until 5 months of age. CD19^+^ CD11c^+^ B cells derived from the BM of WT mice were used as controls (Fig. 5a). Compared with that of the control counterpart, cognitive impairment of the ABC group was greatly aggravated, as evidenced by the decrease in spatial learning and memory performance of 5×FAD mice in both the training trial (Fig. 5b) and probe trial (Fig. 5c) of the Morris water maze (MWM) task. ABCs also obviously impaired performance in the Y maze (Fig. 5d). Furthermore, thioflavin S (TS) staining was performed to examine Aβ plaques in brain sections from FAD-control and FAD-ABC mice to investigate whether ABC injections impact Aβ pathology in AD mice. Indeed, ABC injections significantly increased the Aβ plaque content in the hippocampus and cortex (Fig. 5f, g). Consistent with the TS staining results, ELISA analysis also revealed that the level of TBST-soluble Aβ_42_ was significantly greater in the hippocampus of FAD-ABC mice than in that of FAD-control counterparts (Fig. 5e). Microglia are central players in neuroinflammation that facilitate Aβ production and promote the deposition of Aβ plaques.^42–46^ We measured microglial activity after the injection of ABCs and found that ABC injection augmented reactive microgliosis and reduced the complexity of the microglial branches in 5×FAD mice (Fig. 5h, i). These findings demonstrate that the infiltration of ABCs into the brain accelerates the pathological process of AD.

To decipher specific microglial states associated with the ABCs that infiltrate the brain, single CD11b^+^ cells were sorted from the cortex and hippocampus of FAD-control and FAD-ABCs, and single-cell transcriptomes were obtained via the 10x Genomics platform (supplementary Fig. 6a). A total of 37146 single cells were visualized on UMAP dimensions. The unsupervised clustering reported 8 distinct cell types that were annotated on the basis of cell type-specific markers (supplementary Fig. 6b, c). Among microglia, eleven clusters, including homeostatic (HM), disease-associated (DAM), inflammatory, interferon-responsive (IFN-R), MHC-II, transition and proliferating microglia, were identified (Fig. 5j and supplementary Fig. 6d). The proportion of the HM cluster was lower in the FAD-ABC mice, whereas the proportion of the DAM, inflammation and IFN-R clusters was greater in the FAD-ABC mice (Fig. 5k). Pseudotime analysis revealed that the HM cluster of microglia may differentiate into DAMs in FAD-ABC mice (Fig. 5l). Consistently, the microglia in the FAD-control mice presented an increase in homeostatic marker genes (Cx3cr1, P2ry12 and Tmem119). In contrast, the expression of disease-related marker genes (Trem2, Apoe, Lyz2, Cst7, Lpl, Axl, Itgax and Spp1) was increased in FAD-ABC mice (supplementary Fig. 6e, f). Further immunofluorescence analysis confirmed the decrease in the homeostatic marker P2ry12 and increase in the disease-related marker Cd11c in FAD-ABC mice (supplementary Fig. 6g). GO term analysis revealed that response to interferon-gamma, antigen processing and presentation and positive regulation of the adaptive immune response were among the top altered pathways of DAM in the FAD-ABC group versus the FAD-control group (Fig. 5m). These data demonstrate that at the transcriptional level, ABCs can enhance the maladaptive response of microglia.

To investigate ABC–microglia crosstalk, microglia were treated with conditioned media (CM) from ABCs derived from 5×FAD mice and WT counterparts (supplementary Fig. 7a). The results showed that 5×FAD-derived ABCs increased IL-6 levels (supplementary Fig. 7b) and impaired microglial Aβ_42_-555 phagocytosis (supplementary Fig. 7c, d). Similarly, intracerebroventricular ABC administration to 10-month-old APP/PS1 mice markedly exacerbated the cerebral amyloid burden and maladaptive microglial activation (supplementary Fig. 7e, h). The results suggest that ABCs can augment microglial reactivity and switch on their detrimental effects related to neurodegeneration.^7,9,47^

Collectively, these data indicate that the infiltration of ABCs into the brain parenchyma aggravates Aβ pathology and enhances microglial reactivity in the early AD stage.

### Blockade of IL-6 signaling reduces the number of bone marrow-derived ABCs, cerebral Aβ pathology and cognitive impairments

**Fig. 6:**
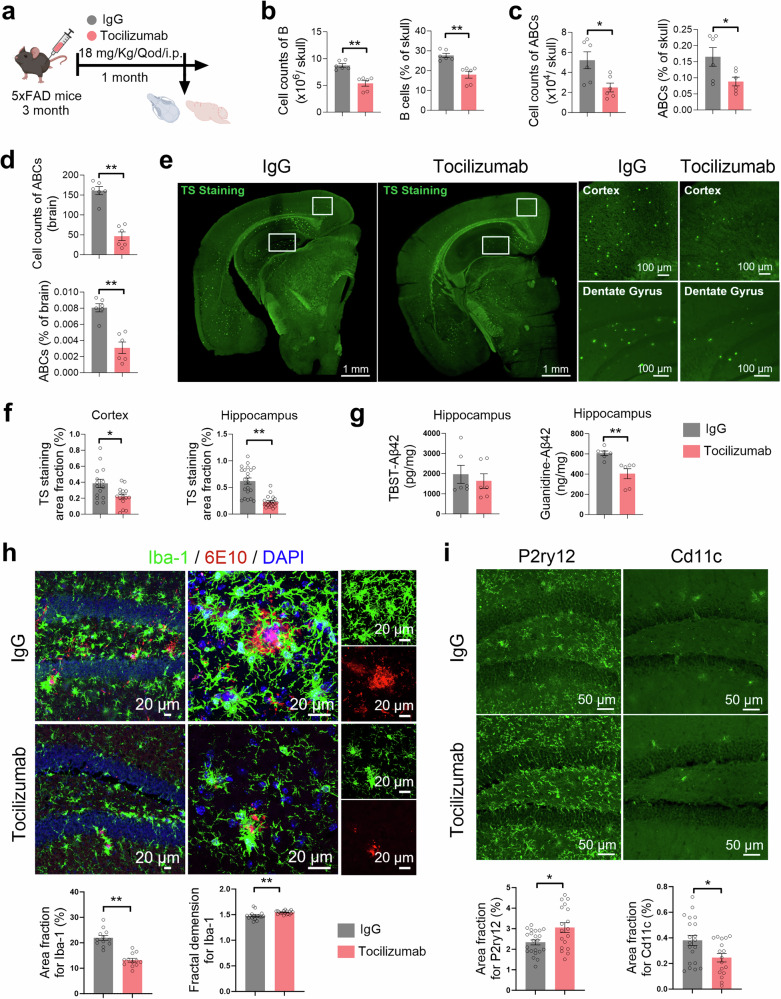
Blockade of IL-6R suppresses Aβ pathology in the early stage in 5×FAD mice. **a** Schematic diagram depicting the experiment in which tocilizumab blocks IL-6R. Tocilizumab (18 mg/kg) or the corresponding IgG control was intraperitoneally injected into 3-month-old 5×FAD mice every other day for 1 month, followed by flow cytometry of the brain and skull BM and immunofluorescence staining of the brain. **b–d** Counts and frequency of B cells (**b**) and ABCs (**c**) in skull bone marrow. Counts and frequency of ABCs in the brain parenchyma (**d**). *n* = 6 5×FAD mice per group. **e–g** Representative images of TS staining of brain sections (left) and enlarged images of the cortex and hippocampal DG region (right) (**e**). Quantification of the TS-positive Aβ plaque area in the cortex and hippocampal DG region (**f**), *n* = 15 views from 3 mice per group for the cortex, *n* = 21 views from 3 mice per group for the DG. Quantification of TBST-soluble Aβ_1-42_ and guanidine-soluble Aβ_1-42_ in the hippocampus via ELISA (**g**), *n* = 6 mice per group. **h** Representative images of Aβ (red) and Iba1 (green) staining in the hippocampal DG region (left) and enlarged images (right) (**h**, upper). Quantification of the Iba1-positive area (left) and Iba1-positive fractal dimension (right) in the hippocampus (**h**, bottom), *n* = 12 views from 3 mice per group for the area fraction, *n* = 18 views from 3 mice per group for the fractal dimension. **i** Representative images of P2ry12 (left) and Cd11c (right) staining in the hippocampal DG region (top). Quantification of P2ry12- and Cd11c-positive areas in the hippocampus (bottom), *n* = 4–7 views from 4 mice per group for the area fraction. The data are presented as the means ± SEMs. *p < 0.05, **p < 0.01; two-tailed unpaired Student’s t test was used

To further investigate the role of IL-6 signaling in bone marrow B-cell production in AD mice, the 3-month-old 5×FAD mice received an intraperitoneal injection of an FDA-approved monoclonal antibody (tocilizumab) that blocks the IL-6 receptor (Fig. 6a). The results showed that tocilizumab treatment markedly reduced counts and frequencies of B cells and ABCs in the skull BM (Fig. 6b, c) and the number of ABCs in the brain parenchyma of AD mice (Fig. 6d). These data evidence that IL-6 signaling plays a key role in bone marrow B lymphopoiesis and the output of ABCs into the brain parenchyma in AD mice.

Next, to explore whether the inhibition of IL-6 signaling confers protection against early AD, we collected brain tissue to measure Aβ deposition. Tocilizumab decreased Aβ deposition in the hippocampal DG and cortex of 5×FAD mice (Fig. 6e, f). Guanidine-soluble Aβ_42_ levels also greatly declined in the hippocampus of 5×FAD mice (Fig. 6g). Moreover, reactive gliosis and a reduction in the complexity of the microglial branches were improved in 5×FAD mice receiving tocilizumab (Fig. 6h). Immunofluorescence analysis confirmed the increase in the homeostatic marker P2ry12 and the decrease in the disease-related marker Cd11c in the 5×FAD-tocilizumab group (Fig. 6i). These results indicate that decreasing the number of BM-derived ABCs by blocking IL-6R can restrain early brain Aβ pathology and microglial reactivity.

Since the increase in the differentiation of HSCs to the B lymphoid lineage persisted in the bone marrow of AD mice (Fig. 2, supplementary Fig. 3 and supplementary Fig. 4), we investigated whether blocking BM B cells would similarly benefit AD mice at intermediate and advanced stages. We intraperitoneally injected 5-month-old 5×FAD mice with tocilizumab every two weeks until 8 months, after which the behavioral tests were performed (supplementary Fig. 8a). Tocilizumab obviously enhanced the performance of the mice in the Y maze (supplementary Fig. 8b). Moreover, tocilizumab treatment significantly improved spatial cognitive impairment, as indicated in the improved learning and memory performance of 5×FAD mice in both the training trial (supplementary Fig. 8c) and probe trial (supplementary Fig. 8d) of the MWM task. Notably, the low-dose and long-term tocilizumab intervention markedly decreased the number of ABCs in the skull BM and brain parenchyma of 5×FAD mice (supplementary Fig. 8e, f). Similarly, tocilizumab diminished the Aβ deposition in the hippocampus and cortex of 5×FAD mice (supplementary Fig. 8g–i). Moreover, gliosis and the complexity of the microglial branches were improved in 5×FAD mice receiving tocilizumab (supplementary Fig. 8j, k). To investigate the long-term effects of IL-6R blockade on AD pathology, 6-month-old 5×FAD mice received intraperitoneal injections (tocilizumab or control IgG) for one month, followed by a 1-month drug washout period. The results demonstrated that even after washout, the tocilizumab-treated group still presented a significantly reduced Aβ burden and attenuated aberrant microglial activation compared with the IgG-treated control group (supplementary Fig. 9).

Altogether, these data demonstrate that the blockade of IL-6 signaling can reduce the number of bone marrow-derived ABCs, restrain cerebral Aβ pathology and decrease microglial reactivity during AD progression.

## Discussion

This study revealed that there was a current under-recognition of BM as a source of B cells governing Aβ pathology in the early stage of AD. PET/CT scanning unexpectedly revealed Aβ deposits within the skull BM of AD patients. Surprisingly, we detected Aβ deposition in the skull BM but not in the femoral BM of young 5×FAD mice or APP/PS1 mice. Importantly, Aβ promotes the proliferation and differentiation of HSCs and B lymphopoiesis in the BM via IL-6 signaling. Notably, in vivo tracing of BM cells revealed that B lymphocytes, particularly ABCs from skull BM, can infiltrate the brain parenchyma and lead to accelerated Aβ deposition and microglial reactivity. Importantly, blocking IL-6R suppresses bone marrow B lymphopoiesis, cerebral Aβ pathology, microglial reactivity and cognitive impairments.

Aβ accumulation occurs naturally during the aging process.^48–50^ In the present study, Aβ accumulated in the skull BM of patients with AD and early AD mice (Fig. 1). Interestingly, Aβ deposition is preferentially found in the subregion of the skull, which corresponds to the frontal lobe, temporal lobe and occipital lobe susceptible to AD.^51,52^ Importantly, we demonstrated that Aβ deposition enhances bone marrow B lymphopoiesis, particularly that of ABCs, in AD model mice (Fig. 4). Combined with in vivo cell labeling in the skull and femur^53^ and flow cytometry analysis, we demonstrated that in the early AD stage in model mice, compared with cells in the femur BM, skull BM-derived B cells were more likely to migrate to adjacent brain tissue (Fig. 3b, f). Nevertheless, femur BM may also be involved in brain B-cell infiltration in late-stage AD mice. Our findings revealed that the CNS adjacent to skull bone marrow can sense the environment earlier, and B cells represent the “first responders” in the context of AD. Recent studies describe the calvaria as a source of meningeal myeloid cells^53^ and B cells,^26^ and indicate that, under homeostasis and inflammation, skull BM provides the CNS with myeloid cells.^26,29^ We further integrate these findings and reveal that these immune cells are involved in the typical neurodegenerative disease AD.

We found that B cells enter the brain, particularly ABCs, and exacerbate Aβ deposition and microglial reactivity (Fig. 5), which is in line with the previous findings that B cells are detrimental to AD.^54^ Instead, the bright spot in our work was that B cells infiltrating the brain parenchyma from the CNS adjacent to the skull bone marrow, not from the circulation. These findings highlight the importance of B cells within skull bone marrow during the initiation and progression of AD. ABCs can produce antibodies and display antigen-presenting and proinflammatory abilities, which are critical components of the immune response and increasingly associated with the pathogenesis of aging-related diseases, autoimmune disorders, and infections.^55^ We show that bone marrow-derived ABCs from AD mice overexpress interferon-stimulated genes, cytokines, adhesion molecules, etc., which likely confers greater damage to the AD brain. Consistently, single-cell transcriptomic analysis of microglia revealed that ABCs accelerate the transition from HMs to DAMs, inflammation, and IFN-R subclusters, and enhance neurodegeneration-associated microglial activity.^7,9,47^ Further cellular experiments demonstrated that AD mouse-derived ABCs promoted inflammatory factor expression in microglia and impaired their phagocytosis of Aβ (supplementary Fig. 7a–d). However, many issues remain to be addressed, such as the possible interaction molecules between ABCs and microglia and the effects of ABC infiltration into the brain on chemotaxis and the activation of peripheral T cells.

The literature indicates that the bone marrow microenvironment is critical for HSC aging.^56^ The senescence of the bone marrow hematopoietic niche drives a shift from asymmetric to symmetric division in HSCs and impairs their self-renewal capacity due to a decrease in key niche factors such as IGF1, Cxcl12, IL7, SCF, and Notch ligands. Moreover, inflammatory mediator-primed accumulation of megakaryocytes (MKs), macrophages (Mφs), plasma cells (PCs), aging-associated B cells, and myeloid-derived suppressor cells (MDSCs) collectively promotes the platelet/myeloid-biased phenotype in HSCs through the secretion of inflammatory cytokines, including CCL5, TNF-α, IL-1β, IFN-γ, Wnt5a, and TGFβ. Our findings demonstrate that Aβ induces HSC proliferation and differentiation and B lymphopoiesis through IL-6 signaling. Bone marrow hematopoiesis is controlled by BM niche cell-derived molecules, including growth factors, chemokines and membrane-binding ligands.^56^ B lymphopoiesis also relies on the production of supportive signals by BM stromal cells^57,58^; in particular, IL-6 plays a critical role in the differentiation and maturation of B cells.^59–61^ In the present study, we provide evidence that IL-6 signaling is a sufficient and necessary condition for B lymphopoiesis in the bone marrow (Fig. 4e–i). Future research urgently requires integration with patient-derived samples to comprehensively elucidate the effects of bone marrow microenvironmental factors on AD-associated hematopoietic dysregulation. Although the cellular components that produce IL-6 in the context of AD await further examination, the priority should focus on B cells as preferential responders so as to appreciate their role in AD development. In addition, where does Aβ in skull BM come from in the early stage? Recent studies revealed that cerebrospinal fluid (CSF) accesses skull BM via dura-skull channels and that CSF-borne cues promote myelopoiesis during spinal cord injury^28^ and bacterial meningitis.^56^ These findings suggest that future studies need to confirm whether the source of Aβ in skull BM involves CSF in AD.

Our findings have clinical relevance. Given that the bone marrow output of ABCs accelerates Aβ neuropathology and microglial reactivity, we propose that the detection of B-cell levels in CSF holds promise as a marker for the early diagnosis of clinical AD. Given that the changes in B cells within the bone marrow during the early AD stages in mice are dominated by an increase in ABCs (Fig. 3e) and that IL-6 is essential for the Aβ-induced upregulation of ABCs (Fig. 4h, i), we treated AD model mice with the IL-6R inhibitor tocilizumab rather than the traditional approach of depleting all B cells with an anti-CD20 antibody. Unsurprisingly, we found that tocilizumab treatment reduced ABCs in the bone marrow and brain of AD mice, restrained brain Aβ pathology, regulated microglial activation, and improved cognitive function in both the early (Fig. 6) and late stages (supplementary Figs. 8, 9). However, Rishi et al. did not find any association between the risk of AD and that of related dementia in patients with rheumatoid arthritis treated with tocilizumab compared with abatacept (a T-cell activation inhibitor).^62^ Our findings indicate that targeting bone marrow B cells with tocilizumab may deserve future investigations in patients with AD. As such, we propose that targeting bone marrow B cells can reduce early cerebral Aβ pathology and perhaps benefit patients with AD. Given that the IL-6 signaling pathway is involved not only in immune regulation but also in broader physiological processes, long-term tocilizumab use requires careful monitoring for hematologic, metabolic, and immune-related off-target effects, balancing therapeutic benefits against systemic physiological disruptions.

Our study revealed that bone marrow-derived B cells accelerate Aβ pathology and exacerbate microglial reactivity and that immune therapy targeting bone marrow-derived B cells via IL-6R blockade may benefit early AD patients. There are several issues to be explored in the future. Owing to the complexity of bone marrow stromal cells, we cannot currently identify the major cell type responsible for both the response to Aβ and the production of IL-6. In the present study, we have not yet been able to directly block the IL-6/IL-6R axis in the bone marrow cavity, and for tocilizumab to affect ABC or B cells to attenuate Aβ pathology, this will need to be addressed with bone marrow chimeras of IL-6R-specific deficient B cells. Owing to ethical limitations, determining how the bone marrow of patients with AD changes is difficult, and a transgenic monkey AD model may be able to answer this question. In addition, APOE4 and TREM2, two important risk factors for AD, are highly expressed in peripheral myeloid cells. Further investigations are warranted to test whether APOE4 and TREM2 can accelerate the pathological process of AD involving bone marrow-derived B cells.

## Materials and methods

### Patients

Patient studies observed the Declaration of Helsinki. The inclusion of human subjects and supporting documentation were granted by the Ethics Committees of The First Affiliated Hospital of Fujian Medical University (Approval No. : MRCTA, ECFAH of FMU [2021]692). All the subjects gave their informed consent at the time of enrollment. PET/CT images were acquired from 8 non-AD dementias (2 males and 6 females) and 18 AD dementias (11 males and 7 females). No marked difference was present in age (66.88 ± 7.16 vs. 68.47 ± 9.61 years, *P* = 0. 681), education (11.63 ± 5.81 vs. 10.76 ± 5.27 years, *P* = 0.716) and MMSE (21.63 ± 5.37 vs. 17.65 ± 5.37, *P* = 0.10) scores of the recruited subjects (supplementary Table 1).

### Animals

The 5×FAD mice, a model that coexpresses five familial AD mutations in the human amyloid precursor protein [K670N/M671L (Swedish) + I716V (Florida) + V717I (London)] and human presenilin 1 (M146L + L286V) under the control of the murine Thy-1 promoter,^63^ were provided by Jackson Laboratory (stock no. 034848‑JAX, Bar Harbor, ME, USA). APP/PS1 mice (stock no. 034832‑JAX), another model that features a mutant human presenilin 1 (PS1-dE9) and a chimeric mouse/human amyloid precursor protein (Mo/HuAPP695swe), and IL-6 knockout mice (B6.129S2-*Il6*^tm1Kopf^/J) (stock no. 002650) were procured from Jackson Laboratory (Bar Harbor, ME, USA). All the mutant mice were backcrossed to the B6 background for 15 generations. The genotypes were verified via a PCR analysis of the tail DNA as documented previously.^64^ Both sexes of mice were enrolled in this study and raised (a maximum of five individuals per cage) in a pathogen-free environment, which was maintained on a controlled light-dark cycle, with a stable temperature of 21 ± 1 °C and a humidity level ranging from 50% to 60%. The animals accessed both water and food unrestrictedly. All animal procedures followed the regulations and policies established by the Institutional Animal Care and Use Committee at Fujian Medical University and adhered to international standards for the ethical treatment of animals.

### Single-cell RNA sequencing

For the intracerebroventricular injection of ABCs, the single-cell sequencing was performed for CD11b^+^ cells from the forebrain and hippocampus of 5×FAD mice. Single-cell capture was achieved via a 10x Genomics single-cell 3’ system. Downstream gene expression matrices were acquired via the function of Cell Ranger with default parameters. Low-quality cells whose gene expression was fewer than 200 or more than 8,000 genes and whose number of mitochondrial genes was >6% and whose genes were expressed in fewer than 3 cells were excluded from further analysis. After filtering, the remaining 18685 MG cells from the control group and 18461 MG cells from the ABC group were analyzed in this study. The data were then normalized via the function ‘NormalizeData’ of Seurat. The top 2000 highly variable genes were recruited via the function ‘FindVariableFeatures’, and then ‘ScaleData’ was adopted. Principal component (PC) analysis was performed via the ‘RunPCA’ function of Seurat. The top 20 PCs were used for dimensionality reduction via the ‘RunUMAP’ function. The cell types were categorized according to the expression of canonical marker genes for each cluster: microglia (Tmem119, Cx3cr1, P2ry12), astrocytes (Gfap, Slc1a2, S100b), BAMs (Mrc1, Ms4a7, Pf4), oligodendrocytes (Mbp, Mog, Olig1), neutrophils (Lcn2, Retnlg, Msrb1), ependymal cells (Foxj1, Ttr, Ak7), endothelial cells (Prom1, Pecam1, Fn1), and T cells (Ccl5, Trbc2, Id2).

After the identification of cell types, the microglial lineage was extracted and subclustered for further analysis. Subclustering was performed via Seurat with the top 11 principal components. The identification of marker genes was accomplished by comparing each cluster with all other clusters via the FindAllMarkers function with default settings (log-fold change threshold of 0.25 and >10% of cells expressing the gene). The cell clusters from each tissue were annotated on the basis of the expression of marker genes. To further assess the activation of microglia, the Monocle package was used to analyze single-cell trajectories to discover developmental transitions. GO enrichment analysis of the differentially expressed gene sets was processed via the clusterProfiler R package. GO terms with adjusted P values less than 0.05 were deemed significantly enriched with DEGs.

### Bone marrow cell dye tracer

The cell tracker was injected into the cranium and femur according to the literature with some modifications,^53^ the bone marrow cell tracer for leg bones was the FITC channel (CellTracker™ Green CMFDA dye), and the bone marrow cell tracer for cranial bones was the APC channel (CellTracker™ dark red dye). Flow cytometric assays were performed on the tracer cells 24 h or 96 h after dye injection. In summary, the procedure for microinjection into the tibial and skull marrow was carried out sequentially under anesthesia with 1.5% isoflurane, utilizing a 5 µl syringe (#65 Hamilton Co., USA) fitted with a custom 34 G blunt needle. A midline incision was created in the skin above the skull to reveal both the anterior (near the bregma) and posterior (including the cerebellum) marrow sites. One or two microinjections were administered at each marrow location (specifically the left frontal, right frontal, and occipital sites). Initially, a 33 G needle was employed for a careful “predrilling” procedure to avoid damaging the inner skull wall. If the procedures failed at this phase, the animals involved were excluded from the study. Approximately 2–3 µl of a red fluorescent tracker was meticulously injected at each site through the previously drilled openings, taking 20–30 seconds per injection, culminating in a total of 10 µl of the red tracker injected across the skull (spanning four injection sites). The injection process was closely observed under a microscope. Subsequently, the skin was sutured using 6–0 silk thread. For the tibial marrow injection, the skin was disinfected and incised just below the knee, where the muscle insertion site was gently scraped off the bone at the designated injection point. A 30 G needle was utilized to perforate the bone wall, after which the needle from the Hamilton syringe was inserted into the marrow cavity to deliver 3 µl of a green cell tracker over a span of 30 s. Finally, the skin was sutured using 6–0 thread.

### Bone marrow cavity injection of Aβ or IL-6

Preparation of oligomeric Aβ_42_: Human Aβ_42_ peptide (AS-20276, AnaSpec) was first dissolved in 1,1,1,3,3,3-hexafluoro-2-propanol (HFIP), evaporated in a hood overnight, and then vacuum freeze-dried at 4 °C for 1 h before the dried film was stored at -80°C. After the preparation of oligomeric Aβ_42_, the dried peptide was dissolved in dimethyl sulfoxide (DMSO) to a final concentration of 5 mM. After brief sonication for 1 min in the bath sonicator, a cold phenol-free F-12 cell culture medium was added to a final concentration of 100 μM Aβ_42_. Finally, this mixture was transferred to 4 °C and incubated for 24 h to generate oligomeric Aβ_42_.

The cranial injection method involves the use of a 33 G needle to first lightly drill the hole, which does not penetrate the skull; then, the injection site at the location of the parietal bone near the sagittal fossa is selected, the 34 G needle is slowly inserted into the lumen, and the microliter syringe (Hamilton) is slowly pushed to reduce fluid leakage. Two sites were injected into the skull, and 3 µl of 0.6 µg oligomeric Aβ was injected into each site. Scrambled Aβ_42_ was used as a control.

IL-6 femur marrow injection method: The disinfected skin was incised open up to the knee, and the muscle insertion site was locally scraped off the bone at the chosen site. A pore was created on the bone wall with a 30 G needle. The needle of the Hamilton syringe was inserted into the marrow cavity to inject 2 µl of 100 ng of IL-6 protein (50 ng/µl) into each femur over 30 s. The skin was sutured with 6–0 thread.

### Tocilizumab administration

Tocilizumab (Roche, Schweiz) was diluted in saline. Briefly, for short-term intervention, we administered tocilizumab (18 mg/kg) or control IgG intraperitoneally to three-month-old mice once every other day for 1 month. For long-term intervention, we administered tocilizumab (8 mg/kg) or control IgG intraperitoneally to five-month-old mice once every 2 weeks for 3 months. The corresponding behavioral experiments were proceeded at the end of the treatment. The animals were divided into IgG-treated and tocilizumab-supplemented groups according to a random number table.

### Statistical analysis

Animals were randomly assigned to treatment groups by Excel-generated random numbers. All analyses were processed by investigators blinded to the grouping. All the experiments reported were duplicated at least twice. All the data were described as the means ± SEMs and analyzed with GraphPad Prism 8.0. Data normality was examined by the Shapiro‒Wilk test. The homogeneity of variance was assessed by Bartlett’s test. For comparisons between two independent, unpaired groups, the data with a normal distribution and homogeneous variances were assessed by the unpaired Student’s t test and otherwise by the Mann‒Whitney U test. Data from three groups with one variable were compared by One-way analysis of variance (ANOVA) followed by Tukey’s post hoc test. Comparisons among multiple groups with two or more variables were analyzed by two-way ANOVA followed by the Bonferroni post hoc correction. MWM analysis was conducted by one-way or multiway repeated-measures ANOVA. The specific statistical methods and statistical parameters are detailed in the figures and figure legends. Significance was set at *p* < 0.05 and expressed as ∗*p* < 0.05, and ∗∗*p* < 0.01, *p* < 0.001.

## Data avilability

Further information and requests for resources and reagents should be directed to and will be fulfilled by the lead contact, Xiaochun Chen (chenxc998@fjmu.edu.cn). The single-cell RNA sequencing and bulk RNA sequencing data used during this study are available from the Genome Sequence Archive (GSA) database with public access (CRA011593; CRA010690). This paper does not report original code. Source data files depicting the quantification values mentioned in the text or plotted in graphs are available in the paper or supplemental information.

## Supplementary information

Supplementary Material

Revised similarity report
