# Supplementary material for: Bone marrow B lymphopoiesis accelerates early cerebral amyloid pathology
Source: Signal Transduct Target Ther. 2025 Sep 18;10:312. doi: 10.1038/s41392-025-02419-0 (PMC12443962; doi:10.1038/s41392-025-02419-0)
Supplement: Supplementary file 1 — Supplementary Material [file 41392_2025_2419_MOESM1_ESM.docx]

Supplementary Materials for

B lymphopoiesis accelerates early cerebral amyloid pathology

Jing Zhang^1†*^, Wenting Fang^1†^, Hanchen Liu^1†^, Ran Li^2†^, Zhibao Zhu^3†^, Xin Wu^1^, Shaobo Yao^3^, Ying Fu^3^, Rui Li^1^, Wanjin Chen^3^, Qinyong Ye^1^, Qiang Liu^1,2*^, Xiaochun Chen^1*^

Correspondence to: chenxc998@fjmu.edu.cn; qliu@tmu.edu.cn; drzj@fjmu.edu.cn

**This PDF file includes:**

Materials and Methods

Supplementary Text

Figures. S1 to S9

Tables S1 to S2

**MATERIALS AND METHODS**

**Resource availability**

**Lead contact**

Further information and requests for resources and reagents should be directed to and will be fulfilled by the lead contact, Xiaochun Chen ([chenxc998@fjmu.edu.cn](mailto:chenxc998@fjmu.edu.cn)).

**Materials availability**

All the datasets presented in the paper are available in the supplemental material or deposited in the indicated repositories as described in the methods section. All RNA sequencing data supporting this study are available for download from the Genome Sequence Archive (GSA) database with public access ([CRA011593](https://ngdc.cncb.ac.cn/gsub/submit/gsa/subCRA018180); [CRA010690](https://ngdc.cncb.ac.cn/gsub/submit/gsa/subCRA016700)).

**Patients**

Patient studies observed the Declaration of Helsinki. The inclusion of human subjects and supporting documentation were granted by the Ethics Committees of The First Affiliated Hospital of Fujian Medical University (Approval No. : MRCTA, ECFAH of FMU [2021]692). All the subjects gave their informed consent at the time of enrollment. PET/CT images were acquired from 8 non-AD dementias (2 males and 6 females) and 18 AD dementias (11 males and 7 females). No marked difference was present in age (66.88± 7.16 vs. 68.47 ±9.61 years, P = 0. 681), education (11.63 ± 5.81 vs. 10.76 ± 5.27 years, P = 0.716) and MMSE (21.63 ± 5.37 vs. 17.65 ± 5.37, P = 0.10) scores of the recruited subjects (**supplementary Table 1**).

**Animals**

The 5×FAD mice, a model that coexpresses five familial AD mutations in the human amyloid precursor protein [K670N/M671L (Swedish) + I716V (Florida) + V717I (London)] and human presenilin 1 (M146L+L286V) under the control of the murine Thy-1 promoter ^1^, were provided by Jackson Laboratory (stock no. 034848‑JAX, Bar Harbor, ME, USA). APP/PS1 mice (stock no. 034832‑JAX), another model that features a mutant human presenilin 1 (PS1-dE9) and a chimeric mouse/human amyloid precursor protein (Mo/HuAPP695swe), and IL-6 knockout mice (B6.129S2-*Il6*^tm1Kopf^/J) (stock no. 002650) were procured from Jackson Laboratory (Bar Harbor, ME, USA). All the mutant mice were backcrossed to the B6 background for 15 generations. The genotypes were verified via a PCR analysis of the tail DNA as documented previously ^2^. Both sexes of mice were enrolled in this study and raised (a maximum of five individuals per cage) in a pathogen-free environment, which was maintained on a controlled light-dark cycle, with a stable temperature of 21 ± 1°C and a humidity level ranging from 50% to 60%. The animals accessed both water and food unrestrictedly. All animal procedures followed the regulations and policies established by the Institutional Animal Care and Use Committee at Fujian Medical University and adhered to international standards for the ethical treatment of animals.

**Human PET/CT imaging**

PET/CT imaging was performed using distinct Aβ tracers across two patient cohorts: 4 non-AD dementia patients and 12 AD dementia patients were scanned with the 18F-92 tracer; 4 non-AD dementia patients and 6 AD dementia patients were scanned with the 18F-AV45 tracer. PET was conducted to assess the brain and bone marrow Aβ burden via a PET/CT system (Biograph mCT64 PET/CT, Siemens Healthcare Molecular Imaging, Knoxville, USA). Before each PET acquisition, low-dose CT scans were acquired from the vertex to mid-thigh via the following parameters: spiral mode with a 120 kVp tube voltage, automated tube current modulation (35--165 mAs), 0.8 s/rotation gantry speed, table feed of 15 mm/rotation, pitch factor of 1.0, 512 × 512 matrix, and 3 mm slice thickness. The PET/CT initiated 60 min after the intravenous injection of 9.02–11.00 mCi (334–407 MBq) 18F-92 or 4.67–5.46 mCi (172–202 MBq) 18F-AV45 for 20 min, with no obvious difference in the injected dose across the recruited subjects (**supplementary Table 1**). Before the PET scans, all patients provided written informed consent. PET data were immediately obtained in 3D acquisition mode over the same anatomical coverage, with 6–8 bed positions (2 min per position), a 128 × 128 matrix, and a 30 cm transaxial field of view. PET images were reconstructed via the filtered back-projection (FBP) algorithm with corrections for decay, normalization, dead time, photon attenuation, scatter and random coincidences and then coregistered and displayed via dedicated software (TrueD software, Siemens).

The PET images were analyzed via SPM12 (http://www.fil.ion.ucl.ac.uk/spm). The images were first transformed into SUVs, corrected for body weight, and then spatially normalized into Montreal Neurological Institute (MNI) stereotactic space. All images were normalized by cerebellar gray matter scaling (defined by the Hammers atlas ^3^) prior to analysis, and an SUV analysis served for pseudoreference tissue independent validation. Aβ tracer uptake was detected in four cortical regions (frontal cortex, parietal cortex, lateral temporal cortex, and posterior cingulate cortex/precuneus), and the global uptake in the brain was subsequently measured by the brain amyloid plaque load (BAPL) system ^4^. We defined amyloid-PET as negative when the visual assessment of Aβ tracer uptake was scored as 1 and positive when it was scored as 2--3. The SUVmax of Aβ was measured within manually defined regions of interest (ROIs) in the skull, lumbar spine, ilium and femur. These values were converted to the SUVr using the cerebellar region as the reference region. Subsequently, region-specific SUVr values were normalized to those of age-matched non-AD dementia patients.

**Single-cell RNA sequencing.**

For the intracerebroventricular injection of ABCs, the single-cell sequencing was performed for CD11b^+^ cells from the forebrain and hippocampus of 5×FAD mice. Single-cell capture was achieved via a 10x Genomics single-cell 3’ system. Downstream gene expression matrices were acquired via the function of Cell Ranger with default parameters. Low-quality cells whose gene expression was fewer than 200 or more than 8,000 genes and whose number of mitochondrial genes was > 6% and whose genes were expressed in fewer than 3 cells were excluded from further analysis. After filtering, the remaining 18685 MG cells from the control group and 18461 MG cells from the ABC group were analyzed in this study. The data were then normalized via the function ‘NormalizeData’ of Seurat. The top 2000 highly variable genes were recruited via the function ‘FindVariableFeatures’, and then ‘ScaleData’ was adopted. Principal component (PC) analysis was performed via the ‘RunPCA’ function of Seurat. The top 20 PCs were used for dimensionality reduction via the ‘RunUMAP’ function. The cell types were categorized according to the expression of canonical marker genes for each cluster: microglia (Tmem119, Cx3cr1, P2ry12), astrocytes (Gfap, Slc1a2, S100b), BAMs (Mrc1, Ms4a7, Pf4), oligodendrocytes (Mbp, Mog, Olig1), neutrophils (Lcn2, Retnlg, Msrb1), ependymal cells (Foxj1, Ttr, Ak7), endothelial cells (Prom1, Pecam1, Fn1), and T cells (Ccl5, Trbc2, Id2).

After the identification of cell types, the microglial lineage was extracted and subclustered for further analysis. Subclustering was performed via Seurat with the top 11 principal components. The identification of marker genes was accomplished by comparing each cluster with all other clusters via the FindAllMarkers function with default settings (log-fold change threshold of 0.25 and >10% of cells expressing the gene). The cell clusters from each tissue were annotated on the basis of the expression of marker genes. To further assess the activation of microglia, the Monocle package was used to analyze single-cell trajectories to discover developmental transitions. GO enrichment analysis of the differentially expressed gene sets was processed via the clusterProfiler R package. GO terms with adjusted P values less than 0.05 were deemed significantly enriched with DEGs.

**Bulk RNA sequencing.**

CD19^+^ CD11C^+^ B cells from skull bone marrow of 5×FAD and WT mice (n = 3 for each group) were isolated using anti-mouse CD19 and CD11c magnetic particles to prepare for RNA sequencing. Total RNA was extracted via a TRIzol reagent kit (Invitrogen) according to the manufacturer’s instructions. The integrity of the RNA was evaluated with an Agilent 2100 Bioanalyzer (Agilent Technologies, Palo Alto, CA, USA) and further verified by RNase-free agarose gel electrophoresis. After the enrichment of eukaryotic mRNA, reverse transcription was conducted to synthesize complementary DNA (cDNA). The resultant cDNA fragments underwent purification, were ligated to Illumina sequencing adapters, and were subsequently sequenced on the Illumina platform by Genedenovo Biotechnology Co., Ltd. (Guangzhou, China). The analysis of differential expression between the two experimental groups was executed with DESeq2 software, with the statistical significance set at a corrected p value of <0.05. KEGG pathway enrichment of DEGs was analyzed by a fold change >1.5 and FDR < 0.05.

**Flow cytometry**

After the anesthetization with isoflurane, the spleen, skull, and femur were then collected from the mice for further cell isolation. To isolate splenocytes, the tissue was ground through a 40 mm cell filter. After the PBS washing and lysing the erythrocytes in ACK lysis buffer, the single cells were resuspended in 1% BSA after PBS rinsing. BM cells were obtained and the femur bone marrow cavity was rinsed with cold PBS. Subsequently, the skull was cut into small pieces and filtered with a 70 mm cell strainer. Then, red blood cells were removed by adding ACK lysis buffer, the samples were washed in cold PBS, and single cells were resuspended in 1% BSA. After pericardial perfusion with cold PBS, the brain tissue was removed, minced, washed with PBS, and incubated in collagenase IV at 37°C for 30 min. Myeline was removed, and 30% Percoll (Sigma Aldrich) was added. Then, the single cells were resuspended in 1% BSA. Cell quantification was performed with an automated cell counter. A fluorophore-conjugated antibody was then added to the cell suspension, which was subsequently incubated on ice for 30 min. For intracellular staining, after surface labeling, the cells were fixed in fixation buffer for 20 min and then washed twice in permeabilization buffer. The samples were further incubated with Ki-67-BV650 (BioLegend) for 30 min. The cells were then rinsed and suspended in a flow cytometric buffer ^5^.

**Bone marrow cell dye tracer**

The cell tracker was injected into the cranium and femur according to the literature with some modifications ^6^, the bone marrow cell tracer for leg bones was the FITC channel (CellTracker™ Green CMFDA dye), and the bone marrow cell tracer for cranial bones was the APC channel (CellTracker™ dark red dye). Flow cytometric assays were performed on the tracer cells 24 hours or 96 hours after dye injection. In summary, the procedure for microinjection into the tibial and skull marrow was carried out sequentially under anesthesia with 1.5% isoflurane, utilizing a 5 µl syringe (#65 Hamilton Co., USA) fitted with a custom 34G blunt needle. A midline incision was created in the skin above the skull to reveal both the anterior (near the bregma) and posterior (including the cerebellum) marrow sites. One or two microinjections were administered at each marrow location (specifically the left frontal, right frontal, and occipital sites). Initially, a 33G needle was employed for a careful "predrilling" procedure to avoid damaging the inner skull wall. If the procedures failed at this phase, the animals involved were excluded from the study. Approximately 2–3 µl of a red fluorescent tracker was meticulously injected at each site through the previously drilled openings, taking 20–30 seconds per injection, culminating in a total of 10 µl of the red tracker injected across the skull (spanning four injection sites). The injection process was closely observed under a microscope. Subsequently, the skin was sutured using 6–0 silk thread. For the tibial marrow injection, the skin was disinfected and incised just below the knee, where the muscle insertion site was gently scraped off the bone at the designated injection point. A 30G needle was utilized to perforate the bone wall, after which the needle from the Hamilton syringe was inserted into the marrow cavity to deliver 3 µl of a green cell tracker over a span of 30 seconds. Finally, the skin was sutured using 6–0 thread.

**Bone marrow cavity injection of Aβ or IL-6**

Preparation of oligomeric Aβ_42_: Human Aβ_42_ peptide (AS-20276, AnaSpec) was first dissolved in 1,1,1,3,3,3-hexafluoro-2-propanol (HFIP), evaporated in a hood overnight, and then vacuum freeze-dried at 4°C for 1 hour before the dried film was stored at -80°C. After the preparation of oligomeric Aβ_42_, the dried peptide was dissolved in dimethyl sulfoxide (DMSO) to a final concentration of 5 mM. After brief sonication for 1 min in the bath sonicator, a cold phenol-free F-12 cell culture medium was added to a final concentration of 100 μM Aβ_42_. Finally, this mixture was transferred to 4°C and incubated for 24 h to generate oligomeric Aβ_42_.

The cranial injection method involves the use of a 33G needle to first lightly drill the hole, which does not penetrate the skull; then, the injection site at the location of the parietal bone near the sagittal fossa is selected, the 34G needle is slowly inserted into the lumen, and the microliter syringe (Hamilton) is slowly pushed to reduce fluid leakage. Two sites were injected into the skull, and 3 µl of 0.6 µg oligomeric Aβ was injected into each site. Scrambled Aβ_42_ was used as a control.

IL-6 femur marrow injection method: The disinfected skin was incised open up to the knee, and the muscle insertion site was locally scraped off the bone at the chosen site. A pore was created on the bone wall with a 30G needle. The needle of the Hamilton syringe was inserted into the marrow cavity to inject 2 µl of 100 ng of IL-6 protein (50 ng/µl) into each femur over 30 seconds. The skin was sutured with 6–0 thread.

**Intracerebroventricular (ICV) injection for ABCs**

5×FAD mice aged 2.5 months and APP/PS1 mice aged 10 months were randomly separated into two groups and subjected to ICV injection of ABCs. Briefly, the mice were deeply anesthetized with isoflurane and immobilized via a stereotactic device. A 0.5 mm burr hole was drilled in the skull, and a guide [cannula](https://www.sciencedirect.com/topics/medicine-and-dentistry/cannula) (62070, RWD) was implanted into the lateral ventricle (AP, +0.4 mm; ML, -/+0.9 mm; DV, - 2.1 mm) of each mouse. Melted agarose was applied around the catheter to seal the gap between the skull and the catheter. The first layer of dental cement is applied to all sides of the exposed skull and catheter. The second layer of dental acrylic cement was then added, and the mixture was left for 10 minutes to allow the dental cement to cure. A stainless-steel cap (62170, RWD) was applied to maintain the cannula [patency](https://www.sciencedirect.com/topics/biochemistry-genetics-and-molecular-biology/patency). The animals were removed from the stereotaxic apparatus and monitored at 37°C until waking. After the animals have recovered for 2 weeks, the ABCs can be injected into the lateral ventricles via a catheter.

ABCs from the bone marrow of 10-month-old 5×FAD mice, 18-month-old APP/PS1 mice, or WT counterparts were obtained via magnetic bead sorting, and fresh ABCs (cell concentration of 1*10^5/μl, 2 μl per lateral catheter injection) were injected by ICV at a rate of 0.2 μl/min via a microliter syringe (Hamilton); the needle was left in place for 5 min for cell infusion. ICV injections were performed once a month for 2 months. One month after the last ABC injection, the cognitive performance of the mice was measured via the MWM and Y maze trials.

**Magnetic bead sorting**

For the ABCs ICV injection experiment, we prepared single-cell suspensions as follows: the skull bone marrow cavity was rinsed with cold PBS and filtered with a 70 µm cell strainer. Then, red blood cells were removed by adding red cell lysate and washed in cold PBS. The single cells were resuspended in 1% BSA and incubated with a PE-conjugated anti-CD19 antibody (BioLegend), followed by staining with an anti-PE multisort kit (130--090--757, Miltenyi Biotec). After the isolation of CD19^+^ cells, multiple microbeads were removed via the multisort release reagent. The CD19^+^ cells were further sorted with CD11c magnetic beads (130-125-835, Miltenyi Biotec) to obtain CD19^+^CD11c^+^ cells. The cells were counted under a microscope, and ICV injection was performed as soon as possible.

One month after ABC ICV injection, the forebrain and hippocampus were dissociated into single-cell suspensions according to the instructions of the Adult Brain Dissociation Kit (130--107--677, Miltenyi Biotec); cell debris, myelin, and erythrocytes were subsequently removed; and the cells were immunolabeled with CD11b microbeads (catalog no. 130--093--634). The cell suspension was passed through the magnetic column to retain CD11b-positive cells. The latter were flushed out and kept for single-cell sequencing.

**Tocilizumab administration**

Tocilizumab (Roche, Schweiz) was diluted in saline. Briefly, for short-term intervention, we administered tocilizumab (18 mg/kg) or control IgG intraperitoneally to three-month-old mice once every other day for 1 month. For long-term intervention, we administered tocilizumab (8 mg/kg) or control IgG intraperitoneally to five-month-old mice once every 2 weeks for 3 months. The corresponding behavioral experiments were proceeded at the end of the treatment. The animals were divided into IgG-treated and tocilizumab-supplemented groups according to a random number table.

**Behavioral tests**

Before the test, the mice were handled for 10 minutes every day for one week. On the testing day, they were allowed 30 min to acclimate the environment. All behavioral experiments were conducted between 8:00 and 17:00 in a dimly lit environment (±20 lux) without food or water deprivation.

The assessment of working memory was implemented using the Y maze following established protocols with slight modifications ^7^. The Y maze consisted of three identical arms designated as A, B, and C. The animals were initially positioned in the start arm (A) and allowed a free exploration for 5 min, during which the sequence of arms explored (for example, ABCBA) was documented. A video recording system was employed to capture the total number of arm entries and instances of behavioral alternation. The proportion of spontaneous alternation was computed automatically through the Noldus behavioral tracking software.

The Morris water maze test was proceeded to assess the spatial learning and memory capabilities of the mice, as previously established with minor adjustments.^8^. Briefly, each mouse underwent acquisition experiments for 6 days and 4 trials per day. They were placed into the water from a predetermined starting position, and the time taken to locate the platform was recorded. On the 7th day, after the platform removal, the mice underwent a probe trial to assess memory retention. The swimming pattern of each animal was continuously monitored via a video camera, with data automatically logged by the tracking software (Noldus).

**ELISA.**

A Human Aβ_42_ ELISA Kit (Invitrogen, KHB3441) and a Human Aβ_42_ ELISA ultrasensitive Kit (Invitrogen, KHB 3544) were used to quantify the levels of Aβ_42_ in the hippocampus and bone marrow, respectively. The detailed experimental procedures are described in our previous article ^2^. The bone marrow proinflammatory cytokine IL-6 was measured via ELISA kits (Ruixinbio, Quanzhou, China; RX203049M).

**Cytokine array analysis.**

The supernatant of the skull marrow was collected for further analysis. The expression of IL-2, IFN-γ, TNF-α, IL-4, IL-5, IL-6, and IL-10 was detected via Luminex. After incubation and washing with antibodies, the levels of these cytokines were measured with a Luminex X-200 system. Luminex200 IS V2.1 software was used for analysis.

**Cell culture**

ABCs were sorted with magnetic beads as previously described and then freshly seeded in U-bottom 96-well plates (2×10^5^ cells/well) in a total volume of 200 μL of serum-free medium (SFM). B cells were activated with a combination of CD40L (0.5 μg/mL, Enzo Life Sciences) and an IgM BCR cross-linking antibody (10 μg/mL, Jackson ImmunoResearch) and washed after 12 h of culture, and the serum-free media was replaced. After a 2-day culture, the ABC supernatant was collected and added to primary microglia (1:1), as detailed below.

As described previously ^9^, primary microglial cultures were prepared. Briefly, mixed glial cultures were derived from WT mice at postnatal days 1 to 2, seeded in flasks coated with poly-l-lysine, and cultured in Dulbecco’s modified Eagle’s medium (DMEM) supplemented with 10% fetal bovine serum (FBS). Ten to 12 days after plating, primary microglia were harvested by shaking (200 rpm, 30 min) and once every 3 days thereafter (up to four harvests). Subsequently, the microglia were plated (2×10^5^ cells per well) and cocultured with the ABC supernatant for a duration of 48 h before RNA isolation or exposure to 10 μM Aβ_42_-555 (AnaSpec). After a 12-hour incubation period, the microglia underwent immunofluorescence analysis.

**RNA isolation and quantitative reverse transcription PCR**

For RNA isolation, total RNA was extracted utilizing TRIzol reagent (Thermo Fisher Scientific, 15596026) and 1 μg of this total RNA was reversely transcribed into complementary DNA (cDNA) with the ReverTra Ace qPCR RT Master Mix (TOYOBO, Osaka, Japan; FSQ-201). The real-time PCR was conducted using a LightCycler 480 System (Roche, Mannheim, Germany) along with the FastStart Universal SYBR Green Master Mix (Roche, 04913850001). The corresponding primer sequences are provided in **supplementary Table 2**. The relative gene expression was calculated by the 2^–ΔΔCt^ method and normalized against the Actin internal control.

**Immunofluorescence and immunohistochemical staining**

After a deep anesthetization with isoflurane, the animals were transcardially perfused with 0.9% NaCl. The brain tissue was removed, soaked in 4% paraformaldehyde solution for 1 day and dehydrated in 30% sucrose solution for 1 week. Coronal sections (40 µm) of mouse brains were placed in cryoprotective storage solution (30% glycerol, 40% 0.1 M PBS, and 30% ethylene glycol) and stored at -20°C before use. Upon use, the sections underwent six TBS washes, an incubation with blocking buffer (0.3% Triton X-100, 0.1% BSA, and 10% donkey serum dissolved in TBS) for 1 h at room temperature, and a further overnight incubation at 4°C with following primary antibodies: Iba-1 (1:500, Wako), 6E10 (1:500, Biolegend), P2ry12 (1:50, Biolegend), Cd11c (1:200, Cell Signaling Technology), and CD19 (1:50, Invitrogen). The secondary antibodies employed in this study consisted of Alexa Fluor 488-conjugated donkey anti-rat IgG, Alexa Fluor 594-conjugated donkey anti-mouse IgG, and Alexa Fluor 488-conjugated donkey anti-rabbit IgG, all diluted at a ratio of 1:2000. Subsequently, the prepared brain slices were affixed to slides coated with polylysine and preserved using a reagent to prevent fluorescence quenching (ProLong Gold Antifade Reagent).

Immunohistochemical staining of the skull BM of the mice was conducted as previously established ^10^ with an UltraSensitive™ SP IHC Kit (KIT- 9720; MXB biotechnologies, China). Floating tissue sections underwent three washing cycles in TBS. Following this, reagents A and B from the kit were administered prior to an overnight incubation with primary antibodies at 4°C. Subsequently, reagents C and D were introduced before the application of a DAB kit (MAX-002; MXB Biotechnologies, China) for staining. The antibodies utilized for immunochemical analysis included 6E10 at a dilution of 1:3000 (Biolegend). Imaging was conducted using an LSM780 confocal microscope (Carl Zeiss, Germany), and statistical evaluations were performed using ImageJ software.

**Thioflavin S (TS) staining**

TS staining was employed to identify Aβ plaques. Specifically, brain sections were subjected to staining with a solution of 0.002% TS (T1892-25G, Sigma‒Aldrich) dissolved in 50% ethanol for a duration of 8 minutes in a dark environment. Subsequently, the sections were rinsed twice with 50% ethanol and thrice with PBS.

**Statistical analysis**

Animals were randomly assigned to treatment groups by Excel-generated random numbers. All analyses were processed by investigators blinded to the grouping. All the experiments reported were duplicated at least twice. All the data were described as the means ± SEMs and analyzed with GraphPad Prism 8.0. Data normality was examined by the Shapiro‒Wilk test. The homogeneity of variance was assessed by Bartlett's test. For comparisons between two independent, unpaired groups, the data with a normal distribution and homogeneous variances were assessed by the unpaired Student's t test and otherwise by the Mann‒Whitney U test. Data from three groups with one variable were compared by One-way analysis of variance (ANOVA) followed by Tukey’s post hoc test. Comparisons among multiple groups with two or more variables were analyzed by two-way ANOVA followed by the Bonferroni post hoc correction. MWM analysis was conducted by one-way or multiway repeated-measures ANOVA. The specific statistical methods and statistical parameters are detailed in the figures and figure legends. Significance was set at p < 0.05 and expressed as ∗p < 0.05, and ∗∗p < 0.01, p < 0.001.

**Supplementary Text**

Graphical abstract


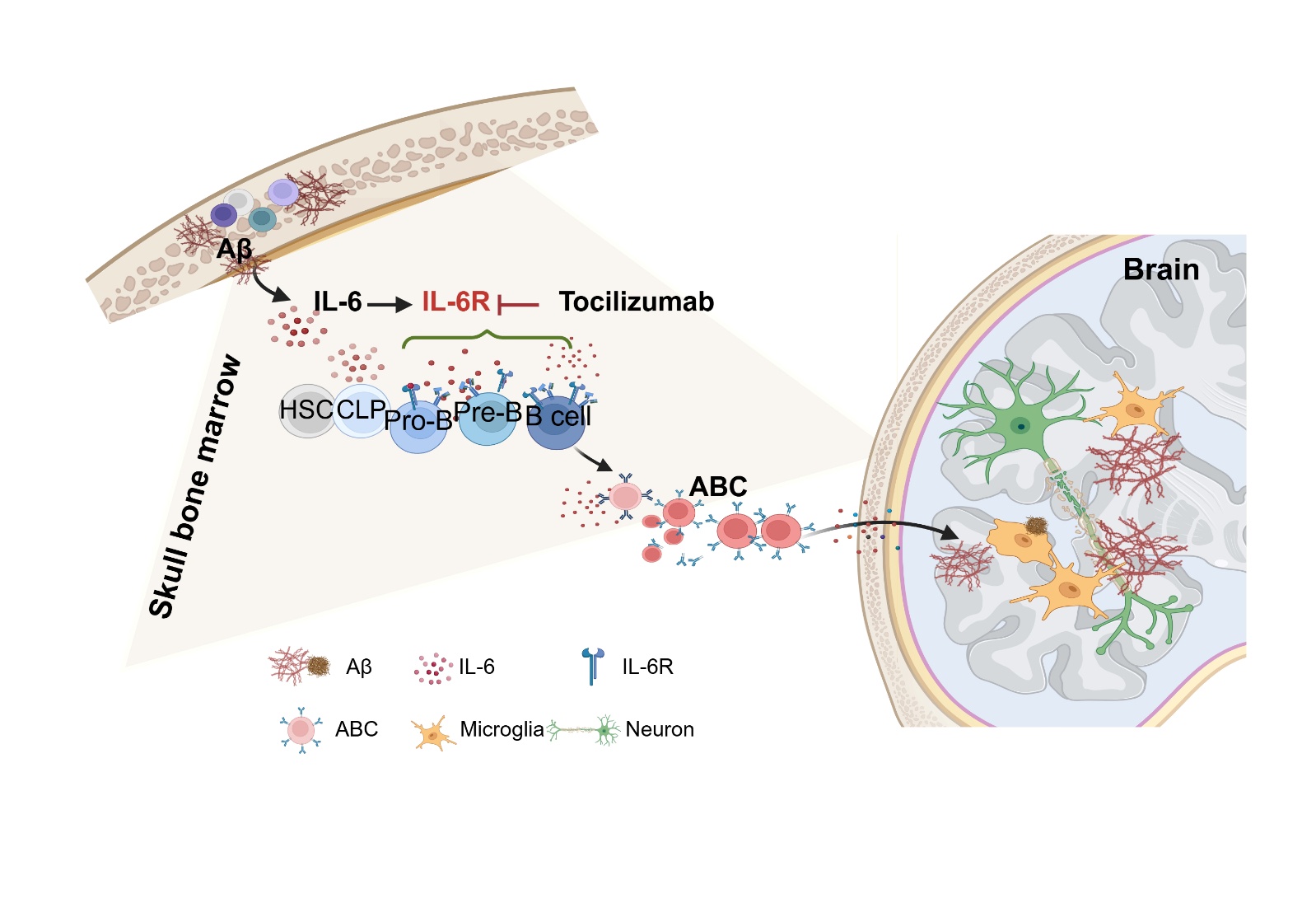


Aβ accumulates early within the central nervous system-surrounding bone marrow, leading to aberrant B lymphopoiesis that involves IL-6 signaling. This results in augmented output of age-associated B cells that accelerate Aβ neuropathology, suggesting that bone marrow-derived B cells may be a target for the future design of immune therapy for Alzheimer’s disease.


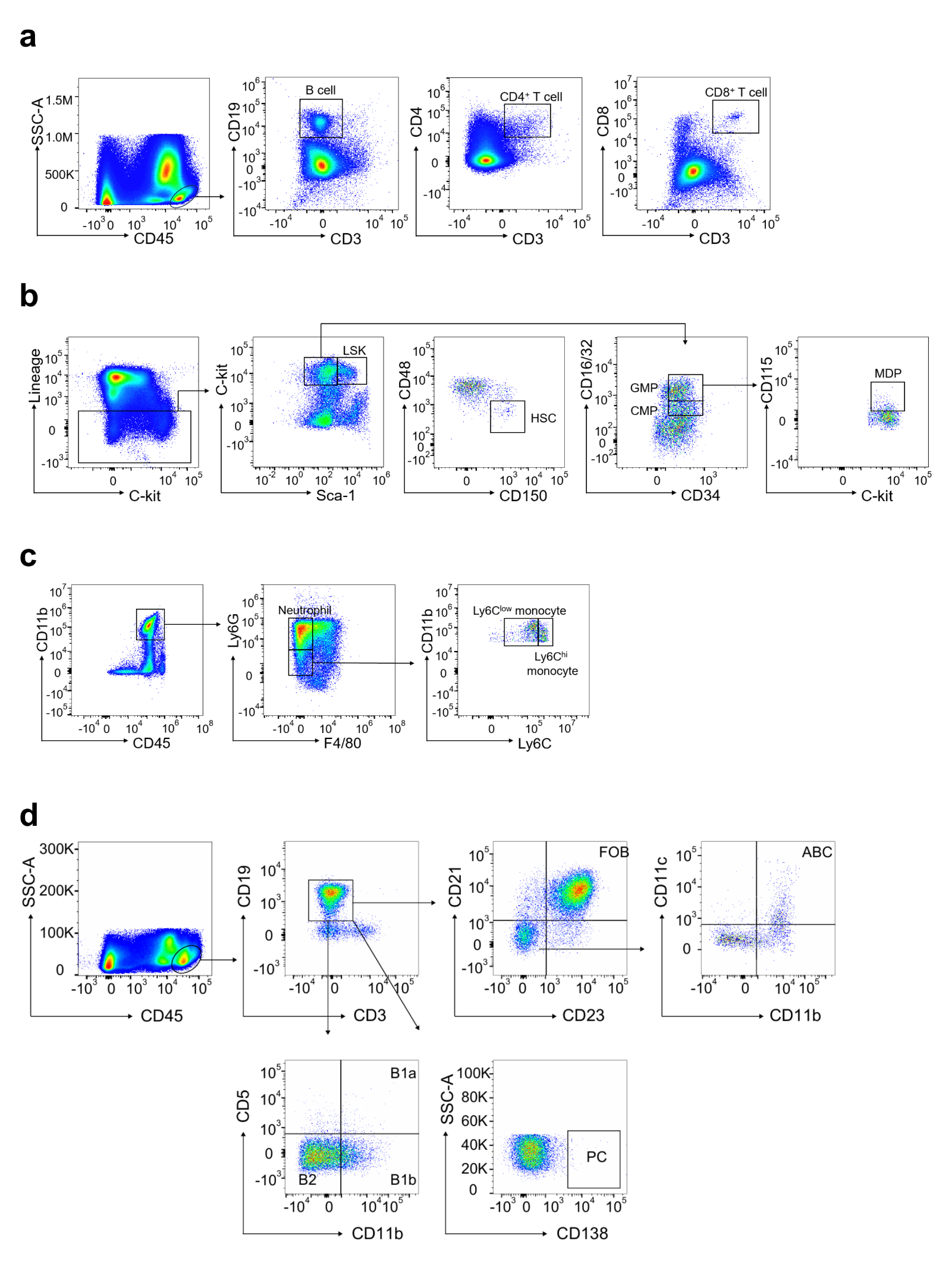
**Figure. S1.**

**Flow cytometry gating strategy of BM cellular lineages in 5×FAD and WT mice, related to Fig. 2-3. a** Flow cytometry gating strategy of BM B cells (CD45^+^ CD3^-^ CD19+), CD4+ T cells (CD45^+^ CD3^+^ CD4^+^), CD8+ T cells (CD45^+^ CD3^+^ CD8^+^). **b** Flow cytometry gating strategy of BM common myeloid progenitors (CMPs, Lin^-^ Sca-1^-^ c-Kit^+^ CD34^+^ CD16/32^int^), granulocyte-monocyte progenitors (GMPs, Lin^-^ Sca-1^-^ c-Kit^+^ CD34^+^ CD16/32^hi^), monocyte-dendritic cell progenitors (MDPs, Lin^-^ Sca-1^-^ c-Kit^+^ CD34^+^ CD16/32^hi^ CD115^+^). **c** Flow cytometry gating strategy of bone marrow neutrophils (CD45^+^ CD11b^+^ Ly6G^+^), Ly6C^high^ monocytes (CD45^+^ CD11b^+^ Ly6G^-^ F4/80^-^ Ly6C^high^), and Ly6C^low^ monocytes (CD45^+^ CD11b^+^ Ly6G^-^ F4/80^-^ Ly6C^low^). **d** Flow cytometry gating strategy of BM plasma cells (PC) (CD45^+^ CD3^-^ CD19^+^CD138^+^), B1a (CD45^+^ CD3^-^ CD19^+^ CD11b^+^CD5^+^), B1b(CD45^+^ CD3^-^ CD19^+^ CD11b^+^CD5^-^), B2(CD45^+^ CD3^-^ CD19^+^CD11b^-^CD5^-^), ABCs (CD45^+^ CD19^+^ CD11b^+^ CD11c^+^ CD21^-^ CD23^-^), CD21^-^CD23^-^B cells (CD45^+^ CD19^+^ CD21^-^CD23^-^), CD21^+^CD23^-^B cells (CD45^+^ CD19^+^ CD21^+^CD23^-^), FOB (CD45^+^ CD19^+^ CD21^+^CD23^+^).


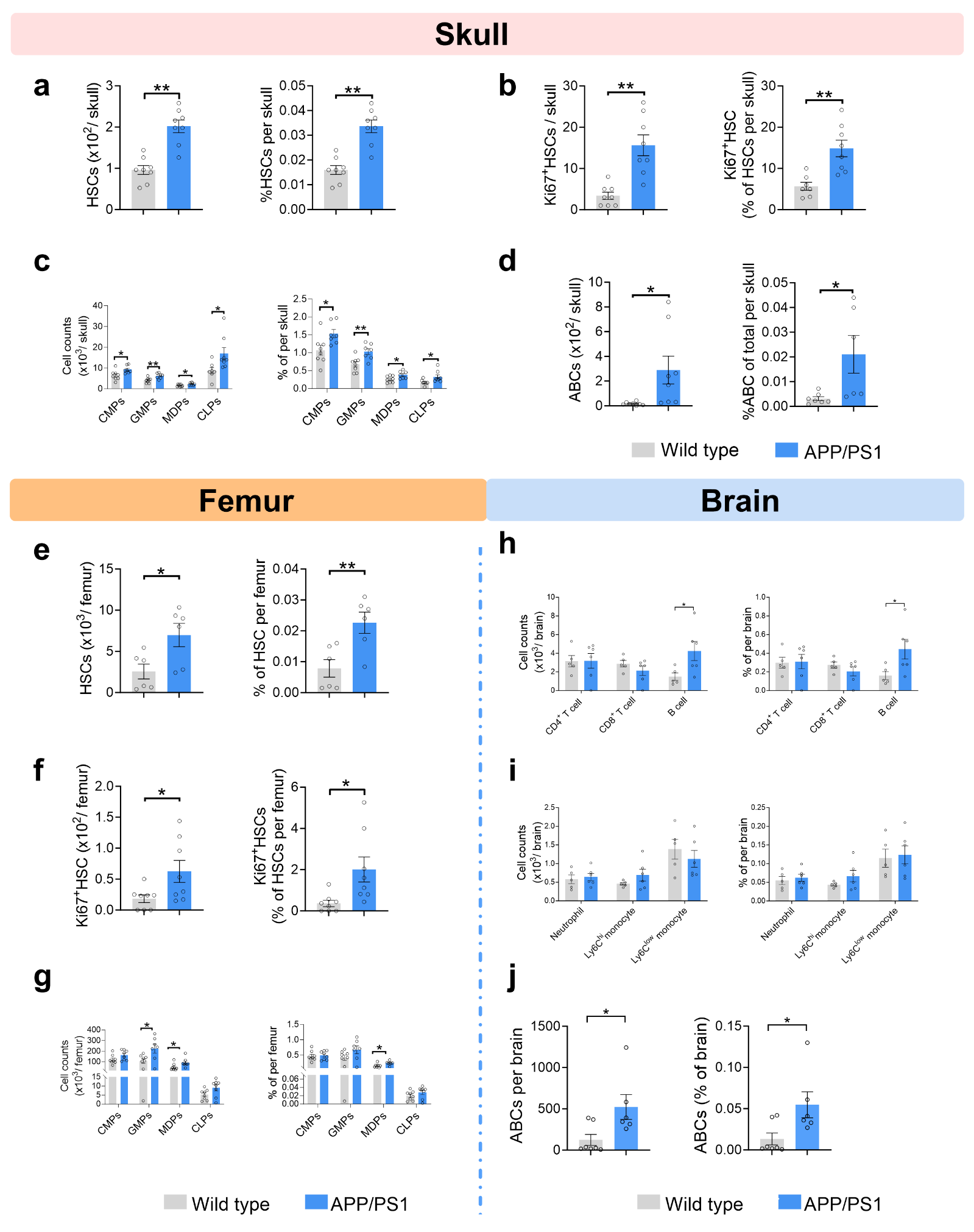
 **Figure. S2.**

**Mobilization of HSCs and increased lymphopoiesis in skull bone marrow of 8-month-old APP/PS1 mice, related to Fig. 2-3.** Flow cytometry detection of HSCs and immune progenitor cells of bone marrow and mature immune cells of brain in 8-month-old APP/PS1 mice and WT counterparts. **a-c** Counts and frequency of HSCs (**a**), Ki67^+^ HSCs (**b**), and immune progenitors (CLPs, CMPs, GMPs, and MDPs) (**c**) in skull bone marrow. n = 6-8 mice per group. **e-g** Counts and frequency of HSCs (**e**), Ki67^+^ HSCs (**f**), and immune progenitors (CLPs, CMPs, GMPs, and MDPs) (**g**) in femur bone marrow. n = 6-8 mice per group.

**h-i** Counts and frequency of mature immune cells including B cells, CD4^+^ T cells, CD8^+^ T cells (**h**), neutrophils, Ly6C^high^ monocytes, and Ly6C^low^ monocytes (**i**) in brain tissue. n = 5-6 mice per group.

**d, j** Counts and frequency of ABCs in skull bone marrow (**d**) and brain tissue (**j**). n = 7 mice per group (**d**)，n = 9 mice per group (**j**). Mean ± SEM. *p < 0.05 and **p < 0.01. Two-tailed unpaired Student’s t test.


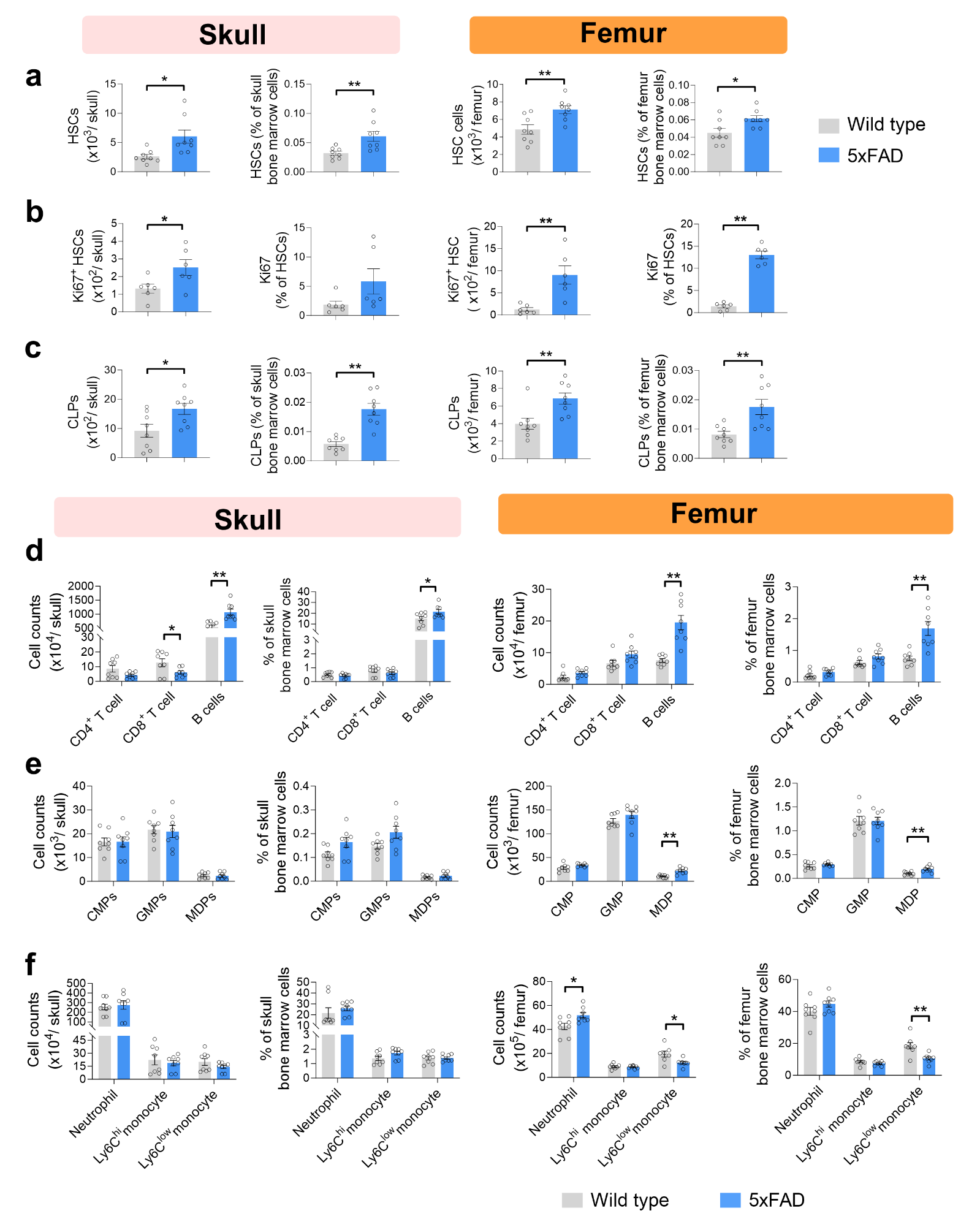
 **Figure. S3.**

**Mobilization of HSCs and increased lymphopoiesis in BM of 10-month-old 5×FAD mice, related to Fig. 2.** Flow cytometry detection of BM HSCs, immune progenitor cells and mature immune cells of 10-month-old 5×FAD mice and WT counterparts. **a-f** Counts and frequency of HSCs (**a**), Ki67^+^ HSCs (**b**), CLPs (**c**), downstream lymphocytes (**d**), other immune progenitors (CMPs, GMPs, and MDPs) (**e**) and myeloid cells (**f**) in skull (left) and femur bone marrow (right). n = 8 mice per group in **a, c, d, e, f**. n = 8 mice per group in **b**. Mean ± SEM. *p < 0.05 and **p < 0.01. Two-tailed unpaired Student’s t test.


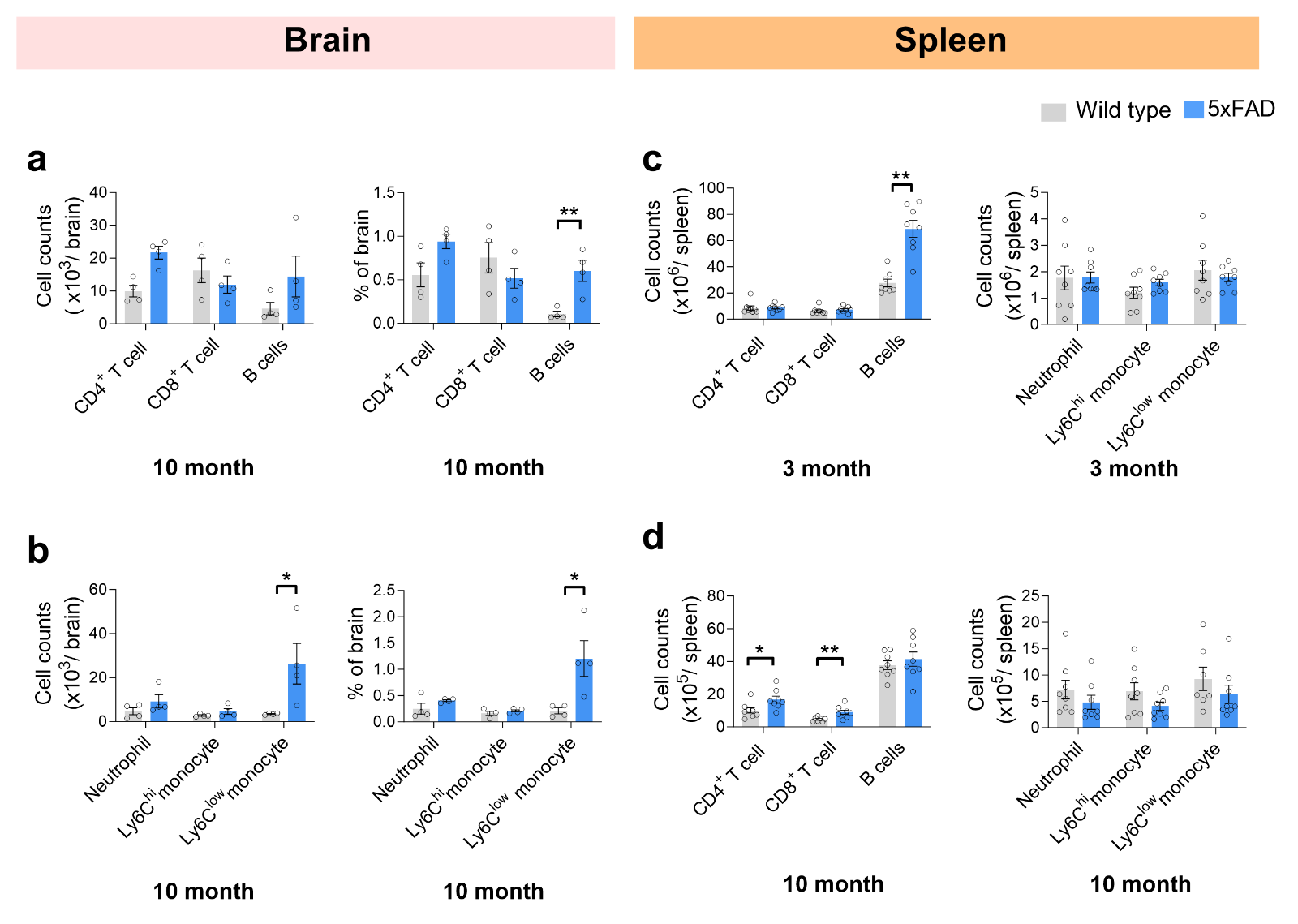
 **Figure. S4.**

**Flow cytometry analysis of brain and spleen cellular lineages in 5×FAD mice, related to Fig. 3. a, b** Counts and frequency of lymphocytes (**a**) and myeloid cells (**b**) of brain in 10-month-old 5×FAD mice and WT mice measured by flow cytometry. n = 4 mice per group. **c, d** Counts of mature immune cell lineage of spleen in 3-month-old (**c**) and 10-month-old (**d**) 5×FAD mice and WT counterparts by flow cytometry. n = 8 mice per group in **c** and **d**. Mean ± SEM. *p < 0.05 and **p < 0.01. Two-tailed unpaired Student’s t test.


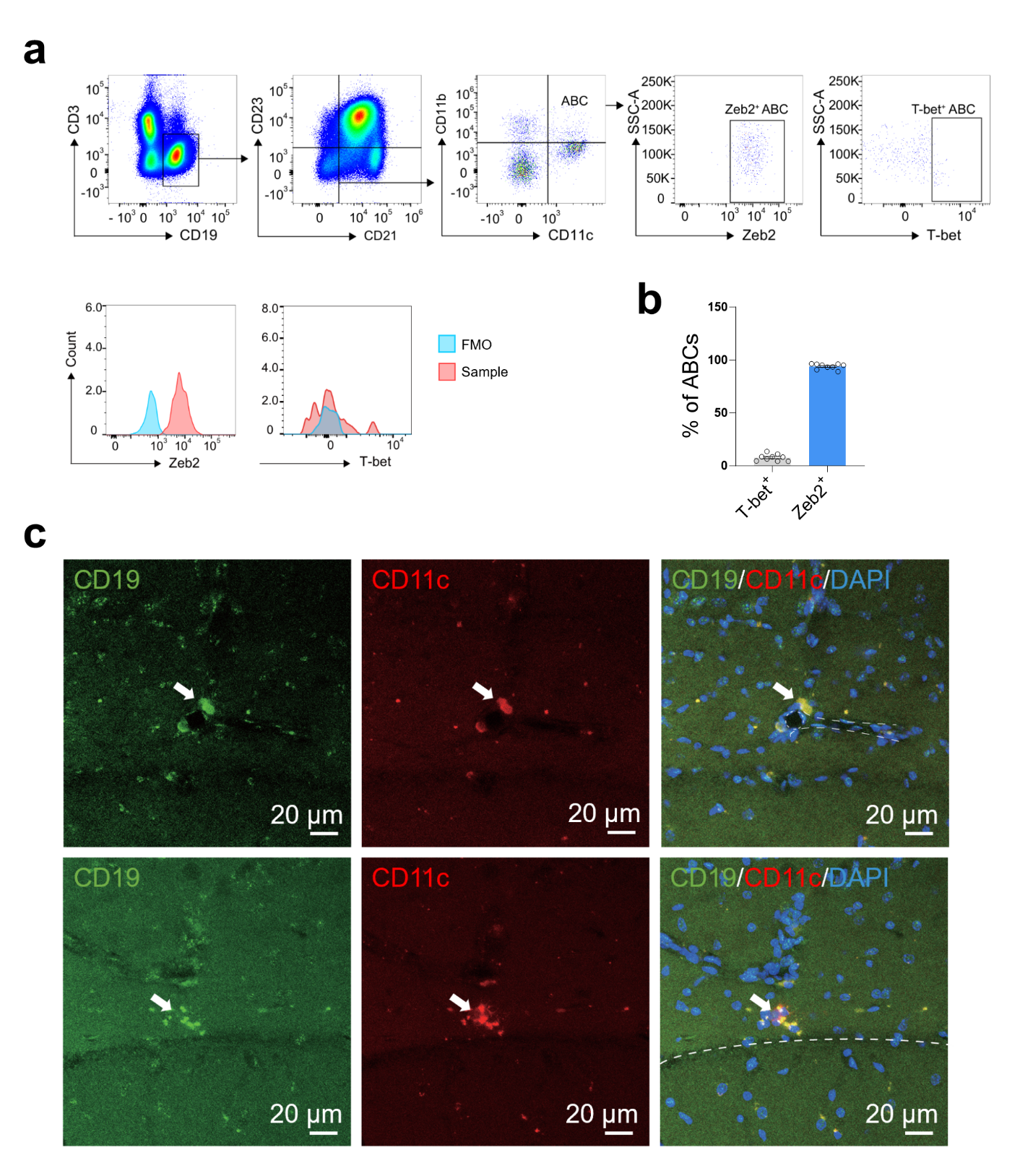


**Figure. S5.**

**Cellular identity of ABCs in 5×FAD mice, related to Fig. 3.** **a, b** Flow cytometry analysis of ABCs in spleen of 3-month-old 5×FAD mice. Gating strategy of Zeb2^+^ ABCs and T-bet^+^ ABCs in spleen (**a**). The percentage of Zeb2^+^ ABCs and T-bet^+^ ABCs within the ABCs (**b**). n = 8 mice per group. **c** Representative images of CD19 and CD11c staining in brain of 10-month-old 5×FAD mice. White arrows indicate positive cells.


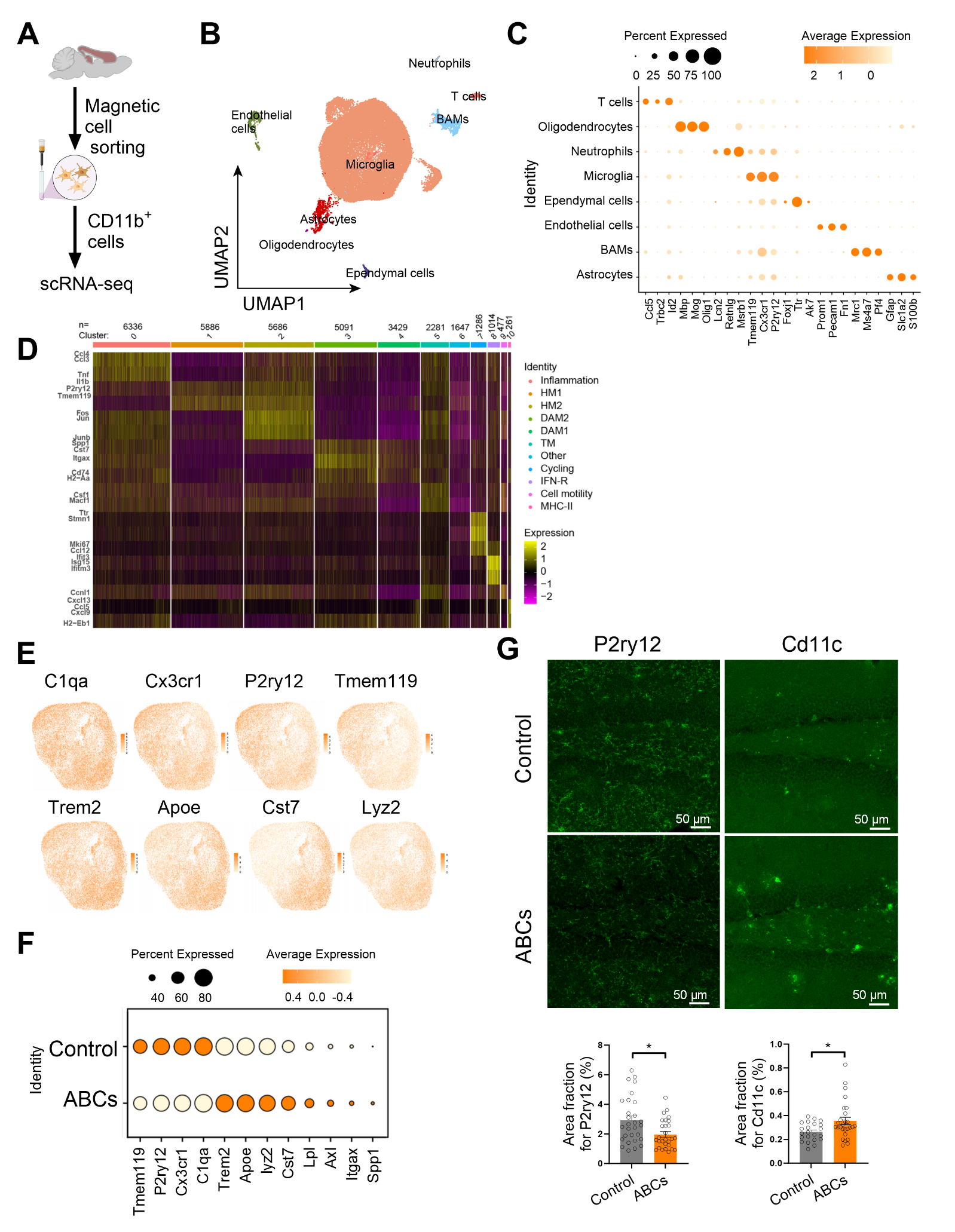


**Figure. S6.**

**Single-cell RNA sequencing of microglia in 5×FAD-control mice and 5×FAD-ABCs mice, related to Fig. 5. a** Experimental design for isolation of CD11b^+^ cells from hippocampus and cortex. **b** UMAP for 37146 cells from n = 2 per group and annotated by cluster. **c** Summarized graph of cell-type-specific marker for all cell types. **d** Gene expression heatmap of the top enriched genes for each microglia cluster. Expression of canonical marker genes delineates 11 microglial states population (HM, homeostatic microglia; DAMs, disease-associated microglia; TM, transitioning microglia; IFN-R, interferon responsive microglia; Inflammation, inflammatory microglia; Cycling, proliferative microglia; MHC-II, MHC class II microglia; Cell motility; Cycling, proliferative microglia). **e, f** UMAP of HM marker genes C1qa, Cx3cr1, P2ry12, Tmem119 and DAM marker genes Trem2, Apoe, Cst7, Lyz2 (**e**). Dot plot of HM and DAM genes (**f**). **g** Representative images of P2ry12 (left) and Cd11c (right) staining in the hippocampal DG region (up). Quantification of P2ry12- and Cd11c- positive area in hippocampus (down), n = 5-8 views from 4 mice per group for area fraction. Data are mean ± SEM. *p < 0.05, Two-tailed unpaired Student’s t test was used.


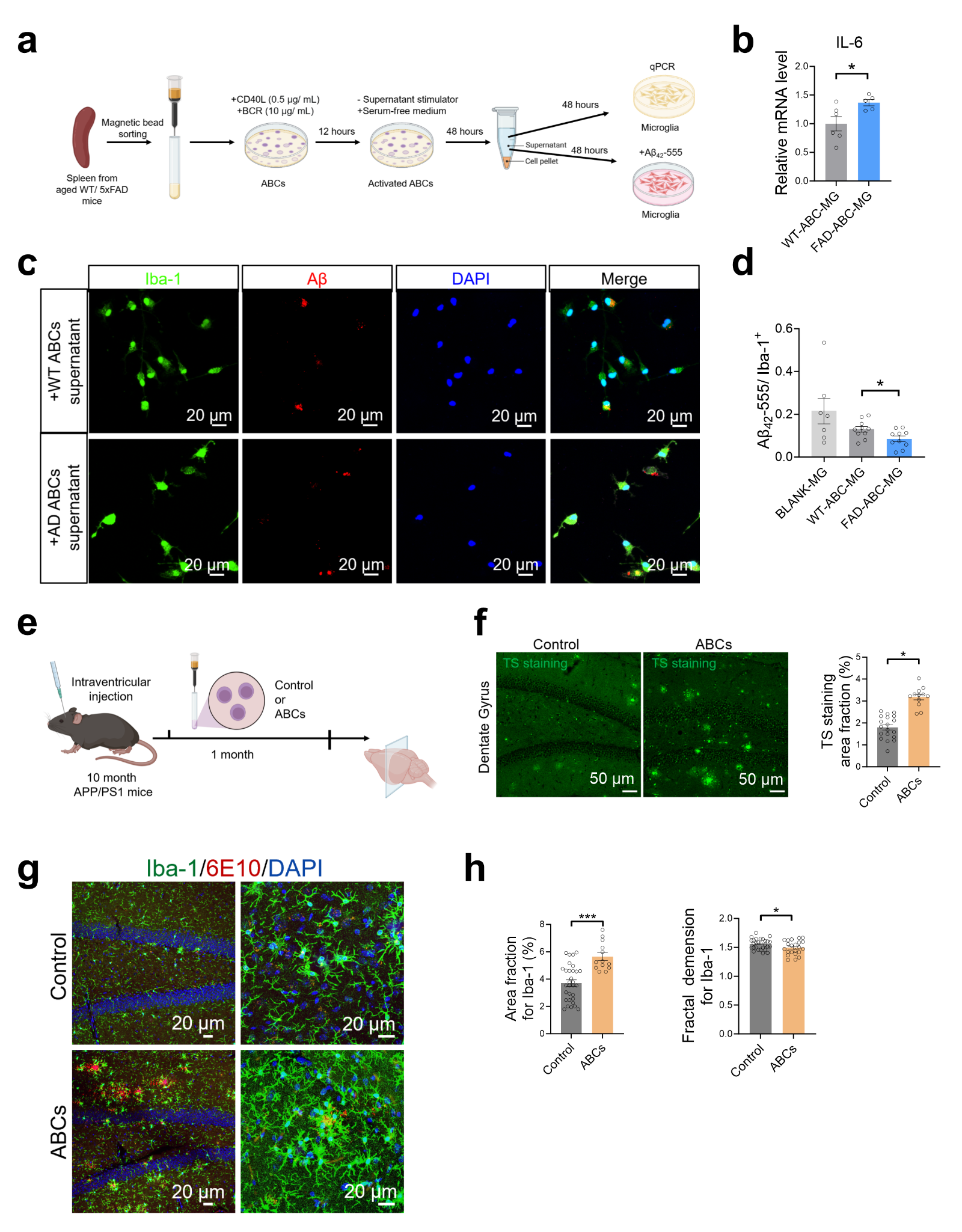


**Figure. S7.**

**ABCs augment Aβ pathology and microglia reactivity, related to Fig. 5. a** Experimental design for primary microglia cultured with supernatant from spleen-derived ABCs in vitro. **b** Quantitative real- time PCR analysis of IL-6 in microglia cultured with supernatant from spleen-derived ABCs in vitro. n = 5-6 per group. **c-d** Primary microglia cultured with supernatant from WT and 5×FAD spleen-derived ABCs for 48 hours and subsequently treated with 10 μM Aβ_42_-555 for another 12 hours, and the phagocytosis capacity of microglia were calculated on the basis of the HiLyte™ Fluor 555 intensity in Iba1^+^ cells. n = 7-9 per group.

**e** Schematic diagram of lateral ventricle injection of ABCs in APP/PS1 mice. The bone marrow derived ABCs from 18-month-old APP/PS1 mice were injected into the bilateral ventricles of 10-month-old APP/PS1 mice, then pathological analysis were performed 1-month post-intervention. **f** Representative images of TS staining in the DG region (left). Quantification of TS-positive Aβ plaque area in hippocampal DG region (right), n = 12-18 views from 4 mice per group. **g-h** Representative images of Aβ (red) and Iba1 (green) staining in the hippocampal DG region (**g**). Quantifications of Iba1-positive area (left) and Iba1-positive fractal dimension (right) in hippocampus (**h**), n = 13-30 views from 4 mice per group for area fraction, n =24-36 views from 4 mice per group for fractal dimension. Data are mean ± SEM. *p < 0.05, **p < 0.01, ***p < 0.001, Two-tailed unpaired Student’s t test was used in **f, h** and Mann-Whitney test was used in **b, d**.


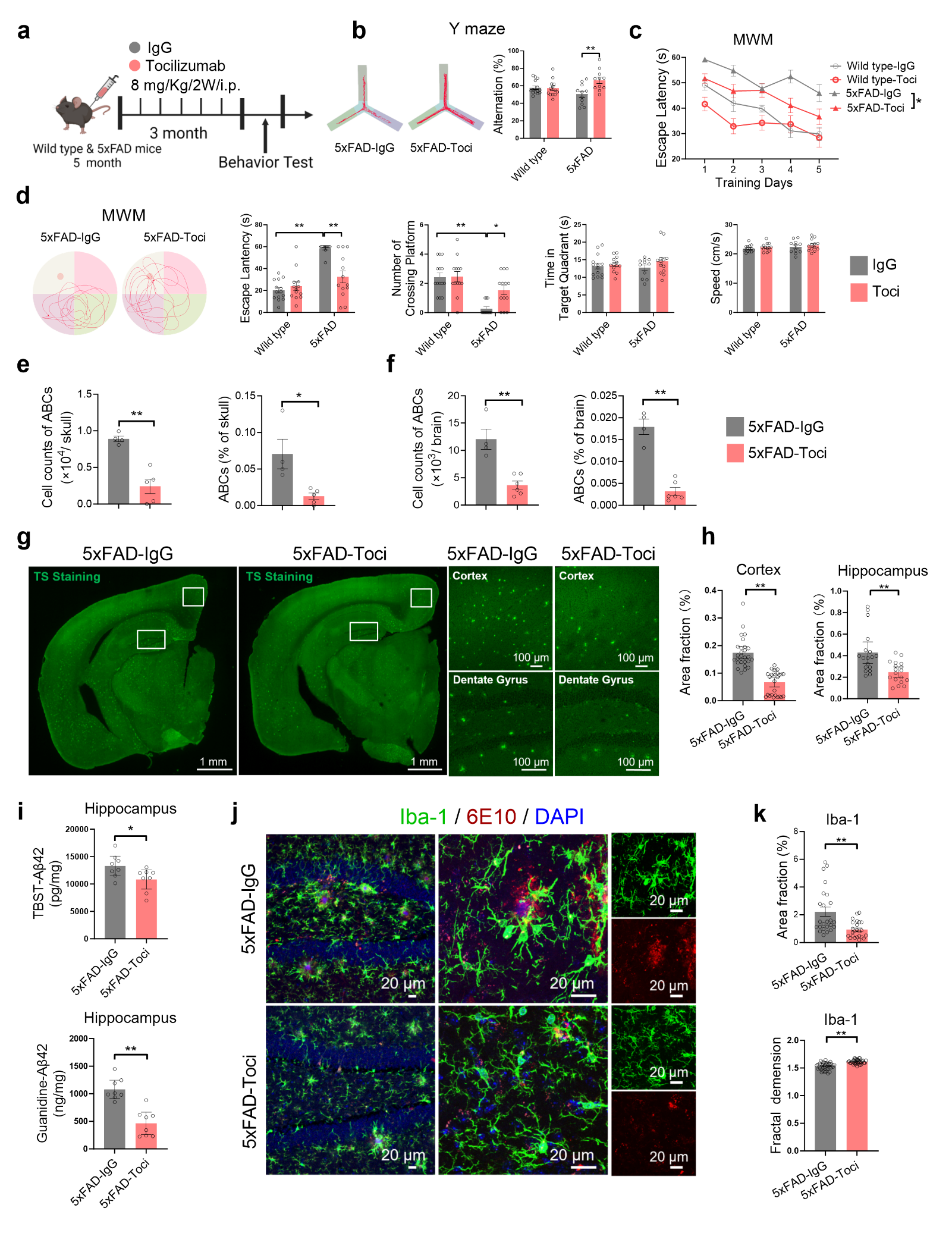


**Figure. S8.**

**Disruption of IL-6R signaling suppresses AD pathological progression and cognitive impairment, related to Fig. 6. a** Schematic diagram of tocilizumab administration in 5×FAD mice. 5-month-old 5×FAD mice were intraperitoneally injected tocilizumab (8mg/kg) or IgG control every two weeks until 8 month, then behavioral test and pathology were performed in turn. In behavioral testing, n=14, 13, 11, and 13 mice for WT-IgG, WT-Tocilizumab, 5×FAD-IgG, and 5×FAD-Tocilizumab, respectively. **b-d** Y maze and Morris Water Maze (MWM) tests. Representative track images and correct spontaneous alternation rate in a Y maze (**b**). Representative track images of 5×FAD mice in the probe trial of MWM and escape latency to the platform during the training trials (**c**). Latency of first time to locate the target, number of target crossings, time spent in target quadrant, and the mean swimming speed of mice in the probe trial (**d**). **e, f** Counts and frequency of ABCs in skull bone marrow (**e**) and brain (**f**). n=4-6 mice per group. **g-i** Representative images of TS staining in the brain sections (left) and the enlarged images of cortex and hippocampal DG region (right) (**g**). Quantification of TS-positive Aβ plaque area in cortex and hippocampal DG region (**h**), n = 27 views from 3 mice per group for cortex, n = 18 views from 3 mice per group for DG. Quantification of TBST-soluble Aβ_1-42_ and Guanidine-soluble Aβ_1-42_ in hippocampus with ELISA, n = 8 mice per group (**i**).

**j, k** Representative images of Aβ (red) and Iba1 (green) staining in the hippocampal DG region (left) and the enlarged images (right) (**j**). Quantifications of Iba1-positive area (up) and Iba1-positive Fractal dimension (down) in hippocampus (**k**), n = 24 views from 3 mice per group for area fraction, n = 36 views from 3 mice per group for fractal dimension. Data are mean ± SEM. *p < 0.05, **p < 0.01; Two-way ANOVA with Tukey's multiple comparisons test was used in **b, d**. Multiway repeated measure ANOVA was used in **c**. Two-tailed unpaired Student’s t test was used in **e, f, h, i** and **k**.


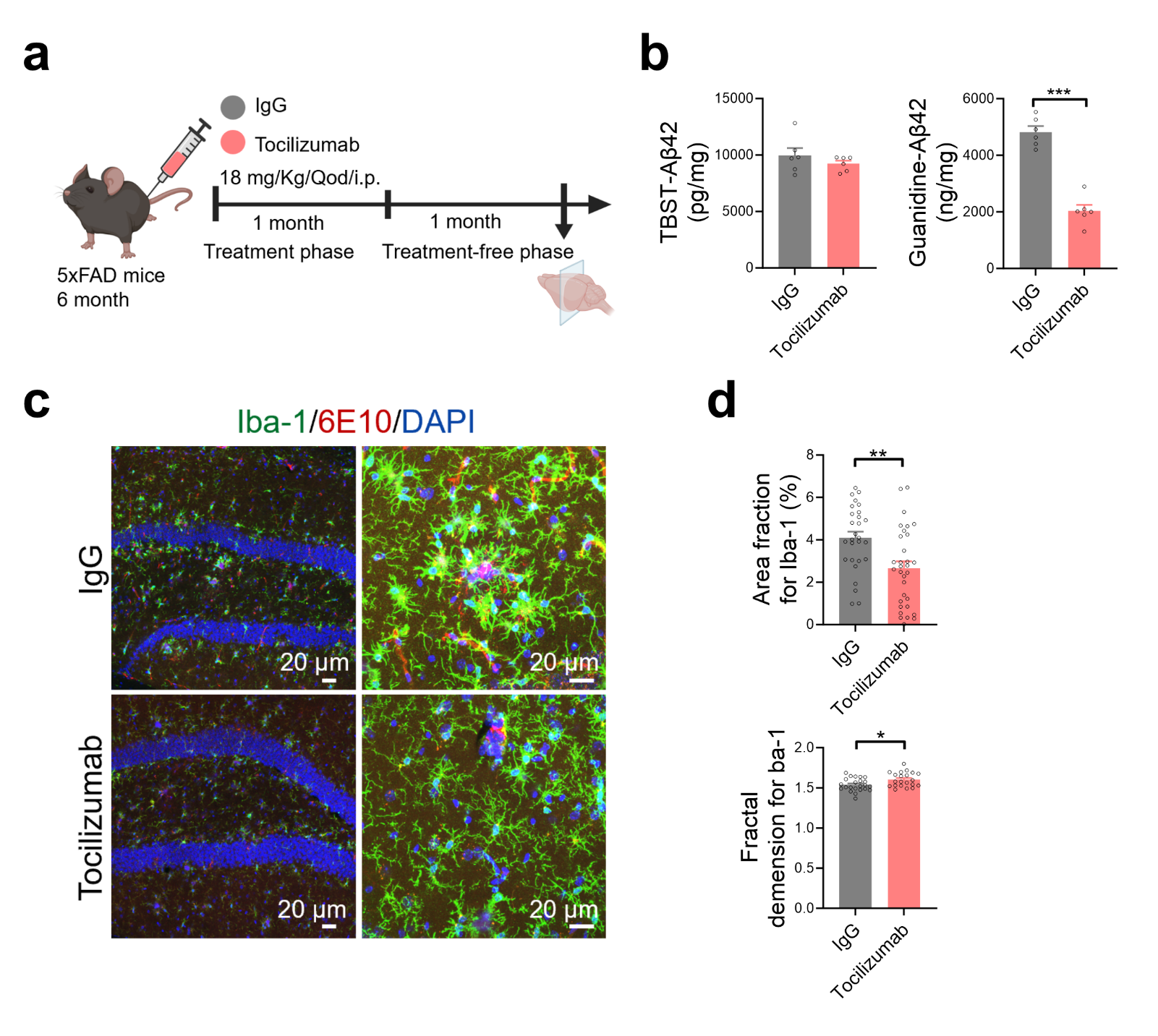
 Figure. S9.

Evaluation of sustained therapeutic efficacy following IL-6R blockade, related to Fig. 6.

**a** Scheme of tocilizumab administration in 5×FAD mice. Tocilizumab (18mg/kg) or matched IgG control were intraperitoneally injected in 6-month-old 5×FAD mice every other day for 1 month. Pathological assessment was carried out after a 1-month period of treatment withdrawal. **b** Quantification of TBST-soluble Aβ_1-42_ and Guanidine-soluble Aβ_1-42_ in hippocampus with ELISA, n = 6-7 mice per group. **c, d** Representative images of Aβ (red) and Iba1 (green) staining in the hippocampal DG region (**c**). Quantifications of Iba1-positive area (up) and Iba1-positive Fractal dimension (down) in hippocampus (**d**), n = 30 views from 4 mice per group for area fraction, n = 24 views from 4 mice per group for fractal dimension. Data are mean ± SEM. *p < 0.05, **p < 0.01; ***p < 0.001; Two-way ANOVA with Tukey's multiple comparisons test was used.

**Table S1. Baseline characteristics and injected dose of 18F-92 or 18F-AV45.**

| Observation item | Non-AD dementia (n=8) | AD dementia  (n=18) | P-value |
| --- | --- | --- | --- |
| Male, n (%) | 2 (25.0%) | 11 (61.1%) | 0.202 |
| Age (year) | 66.88± 7.16 | 68.47 ±9.61 | 0.681 |
| Education (year) | 11.63 ± 5.81 | 10.76 ± 5.27 | 0.716 |
| MMSE (score) | 21.63 ± 5.37 | 17.65 ± 5.37 | 0.10 |
| Injected dose of 18F-92 (mCi) | 9.95 ± 0.37 | 9.72 ± 0.47 | 0.396 |
| Injected dose of 18F-AV45 (mCi) | 5.15 ± 0.17 | 5.20 ± 0.29 | 0.610 |
| Non-AD dementia was defined as a Brain amyloid plaque load (BAPL) score of 1, AD dementia was defined as a (BAPL) score of 2-3 in the brain parenchyma.  MMSE: Mini⁃Mental State Examination, mCi: Milli Curie.  Data are (mean ± SD) or number (%), *P < 0.05. | | | |

**Table S2.**

| Gene name | Forward primer（5’→3’） | Reverse Primer（5’→3’） |
| --- | --- | --- |
| IL6 | TAGTCCTTCCTACCCCAATTTCC | TTGGTCCTTAGCCACTCCTTC |
| β-actin | GGTCATCACTATTGGCAACG | TCCATACCCAAGAAGGAAGG |

**Resources Table**

| \| **REAGENT or RESOURCE** \| **SOURCE** \| **IDENTIFIER** \| \| --- \| --- \| --- \| \| **Antibodies** \| \| \| \| Brilliant Violet 421™ anti-mouse Lineage Cocktail (17A2; RB6-8C5; RA3-6B2; Ter-119; M1/70) \| Biolegend \| Cat# 133311, RRID: AB_11203535 \| \| Brilliant Violet 605™ anti-mouse Ly-6A/E (Sca-1) antibody (D7) \| Biolegend \| Cat# 108134, RRID: AB_2650926 \| \| Brilliant Violet 510™ anti-mouse CD117 (c-kit) antibody (ACK2) \| Biolegend \| Cat# 135119, RRID: AB_2562011 \| \| APC/Fire™ 750 anti-mouse CD34 Antibody (HM34) \| Biolegend \| Cat# 128614, RRID: AB_2715985 \| \| PE Rat Anti-Mouse CD135 (FLK2) antibody (A2F10.1) \| BD Biosciences \| Cat# 553842, RRID: AB_395079 \| \| APC anti-mouse CD48 Antibody (HM48-1) \| Biolegend \| Cat# 103432, RRID: AB_2561463 \| \| PE/Dazzle™ 594 anti-mouse CD150 (SLAM) Antibody (TC15-12F12.2) \| Biolegend \| Cat# 115936, RRID: AB_2565961 \| \| FITC anti-mouse CD16/32 Antibody (93) \| Biolegend \| Cat# 101306, RRID: AB_312805 \| \| Brilliant Violet 650™ anti-mouse CD115 antibody (AFS98) \| BD Biosciences \| Cat# 750891, RRID: AB_2874987 \| \| PE/Dazzle™ 594 anti-mouse CD127 (IL-7Rα) Antibody (A7R34) \| Biolegend \| Cat# 135032, RRID: AB_2564217 \| \| Brilliant Violet 421™ anti-mouse CD45 Antibody (30-F11) \| Biolegend \| Cat# 103134, RRID: AB_2562559 \| \| APC-Cy™7 anti-Mouse CD45 Antibody \| BD Biosciences \| Cat# 557659, RRID: AB_396774 \| \| Brilliant Violet 510™ anti-mouse/human CD11b Antibody (M1/70) \| Biolegend \| Cat# 101263, RRID: AB_2629529 \| \| PE/Cyanine7 anti-mouse/human CD11b Antibody \| Biolegend \| Cat# 101216, RRID: AB_312799 \| \| APC anti-mouse F4/80 Antibody (BM8) \| Biolegend \| Cat# 123116, RRID: AB_893481 \| \| FITC anti-mouse Ly-6G Antibody (1A8) \| Biolegend \| Cat# 127606, RRID: AB_1236494 \| \| PE anti-mouse Ly-6C Antibody (HK1.4) \| Biolegend \| Cat# 128008, RRID: AB_1186132 \| \| Brilliant Violet 510™ anti-mouse CD3 Antibody (17A2) \| Biolegend \| Cat# 100234, RRID: AB_2562555 \| \| Brilliant Violet 785™ anti-mouse CD3 Antibody \| Biolegend \| Cat# 100232, RRID: AB_2562554 \| \| PE anti-mouse CD4 Antibody (GK1.5) \| Biolegend \| Cat# 100408, RRID: AB_312693 \| \| FITC anti-mouse CD8a Recombinant Antibody (QA17A07) \| Biolegend \| Cat# 155004, RRID: AB_2750211 \| \| Brilliant Violet 605™ anti-mouse CD19 Antibody (6D5) \| Biolegend \| Cat# 115540, RRID: AB_2563067 \| \| FITC anti-mouse CD19 Antibody \| BD Biosciences \| Cat# 557398, RRID: AB_396681 \| \| PE anti-mouse CD19 Antibody \| Biolegend \| Cat# 152408, RRID: AB_2629817 \| \| APC anti-mouse NK-1.1 Antibody (PK136) \| Biolegend \| Cat# 108710, RRID: AB_313397 \| \| Alexa Fluor 700 anti-mouse CD21/CD35 (CR2/CR1) Antibody (7E9) \| Biolegend \| Cat# 123432, RRID: AB_2860650 \| \| Brilliant Violet 421™ anti-mouse CD21/CD35 (CR2/CR1) Antibody \| Biolegend \| Cat# 123422, RRID: AB_2650891 \| \| PE/Dazzle 594 anti-mouse CD23 Antibody (B3B4) \| Biolegend \| Cat# 101634, RRID: AB_2687194 \| \| Brilliant Violet 605™ anti-mouse CD23 Antibody \| Biolegend \| Cat# 101637, RRID: AB_2832279 \| \| APC/Cyanine7 anti-mouse/human CD11b Antibody (M1/70) \| Biolegend \| Cat# 101226, RRID: AB_830642 \| \| PerCP/Cyanine5.5 anti-mouse CD11c Antibody \| Biolegend \| Cat# 117328, RRID: AB_2129641 \| \| PE anti-mouse CD11c Antibody (N418) \| Biolegend \| Cat# 117308, RRID: AB_313777 \| \| 7-AAD Viability Staining Solution \| Biolegend \| Cat# 420404 \| \| Brilliant Violet 650™ anti Ki-67 Antibody (B56) \| BD Biosciences \| Cat# 563757, RRID: AB_2688008 \| \| APC anti-Mouse CD138 Antibody \| BD Biosciences \| Cat# 558626, RRID: AB_1645216 \| \| Alexa Fluor® 700 anti-mouse CD5 Antibody \| Biolegend \| Cat# 100636, RRID: AB_2687002 \| \| PE-SIP1/ZEB2 (E-11) Antibody \| Santa \| Cat# sc-271984PE \| \| BV786 anti-mouse T-bet Antibody \| BD Biosciences \| Cat# 564141 \| \| Fixable Viability Stain 510 \| BD Biosciences \| Cat# 564406, RRID: AB_2869572 \| \| CD19 microbeads, mouse \| Milteny Biotec \| 130-121-301 \| \| CD11c microbeads, mouse \| Milteny Biotec \| 130-125-835 \| \| CD11b microbeads, mouse \| Milteny Biotec \| 130-093-634 \| \| Anti-PE MultiSort Kit \| Milteny Biotec \| 130-090-757 \| \| Anti-Iba1 Antibody \| Abcam \| ab178846 \| \| Purified anti-β-Amyloid, 1-16 Antibody \| Biolegend \| 803001 \| \| Purified anti-mouse P2RY12 Antibody \| Biolegend \| 848002 \| \| CD11c (D1V9Y) Rabbit mAb \| Cell signaling technology \| 97585 \| \| CD19 Monoclonal Antibody (6OMP31) \| Invitrogen \| Cat# 14-0194-82, RRID: AB_2637171 \| \| Alexa Fluor 488-conjugated donkey anti-rabbit IgG \| Invitrogen \| A21206 \| \| Alexa Fluor, 594-conjugated donkey anti-mouse IgG \| Invitrogen \| A21203 \| \| **Chemicals, peptides, and recombinant proteins** \| \| \| \| Thioflavin S \| Sigma‒Aldrich \| Cat# T1892-25G \| \| Human Aβ_1-42_ peptides \| AnaSpec \| Cat# AS-20276 \| \| Scrambled Aβ_1-42_ peptides \| AnaSpec \| Cat# AS-25382 \| \| Deep red CellTracker™ dye \| Molecular Probes \| [C34565](https://www.ncbi.nlm.nih.gov/nuccore/C34565) \| \| CMFDA (green) CellTracker™ dye \| Molecular Probes \| C7025 \| \| Tocilizumab \| Roche \| N/A \| \| IgG isotype control \| BioXCell \| BP0297 \| \| IL-6 Protein, Mouse \| MedChemExpress \| HY-P7063 \| \| BD Pharmingen™ Leukocyte Activation Cocktail, with BD GolgiPlug™ \| BD Biosciences \| Cat# 550583, RRID: AB_2868893 \| \| Beta-Amyloid (1-42), HiLyte™ Fluor 555 \| AnaSpec \| AS-60480-01 \| \| **Critical commercial assays** \| \| \| \| Human Aβ_1-42_ ultrasensitive ELISA kit \| Invitrogen \| KHB3544 \| \| Human Aβ_1-42_ ELISA kit \| Invitrogen \| KHB3441 \| \| IL-6 ELISA kit \| Ruixinbio \| RX203049M \| \| Mouse IgG (Immunoglobulin G) ELISA Kit \| elabscience \| E-EL-M0692 \| \| Mouse IgA (Immunoglobulin A) ELISA Kit \| elabscience \| E-EL-M0690 \| \| Mouse IgM (Immunoglobulin M) ELISA Kit \| elabscience \| E-EL-M3036 \| \| **Experimental models: Organisms/strains** \| \| \| \| Mouse: 5×FAD \| Inhouse breeding \| N/A \| \| Mouse: APP/PS1 \| Inhouse breeding \| N/A \| \| Mouse: C57BL/6J \|  \| N/A \| \| Mouse: IL-6 knock out \| Inhouse breeding \| N/A \| \| **Software and algorithms** \| \| \| \| EthoVision XT \| Noldus \| [https://www.noldus.com](https://www.noldus.com/) \| \| GraphPad Prism version 8.0 \| GraphPad Software \| [https://www.graphpad.com](https://www.graphpad.com/) \| \| ImageJ \| National Institutes of Health \| <https://imagej.nih.gov/ij/download.html> \| \| **Other** \| \| \| \| Guide cannula \| RWD \| Cat# 62070 \| \| Stainless-steel cap \| RWD \| Cat# 62170 \| |
| --- | --- | --- | --- | --- | --- | --- | --- | --- | --- | --- | --- | --- | --- | --- | --- | --- | --- | --- | --- | --- | --- | --- | --- | --- | --- | --- | --- | --- | --- | --- | --- | --- | --- | --- | --- | --- | --- | --- | --- | --- | --- | --- | --- | --- | --- | --- | --- | --- | --- | --- | --- | --- | --- | --- | --- | --- | --- | --- | --- | --- | --- | --- | --- | --- | --- | --- | --- | --- | --- | --- | --- | --- | --- | --- | --- | --- | --- | --- | --- | --- | --- | --- | --- | --- | --- | --- | --- | --- | --- | --- | --- | --- | --- | --- | --- | --- | --- | --- | --- | --- | --- | --- | --- | --- | --- | --- | --- | --- | --- | --- | --- | --- | --- | --- | --- | --- | --- | --- | --- | --- | --- | --- | --- | --- | --- | --- | --- | --- | --- | --- | --- | --- | --- | --- | --- | --- | --- | --- | --- | --- | --- | --- | --- | --- | --- | --- | --- | --- | --- | --- | --- | --- | --- | --- | --- | --- | --- | --- | --- | --- | --- | --- | --- | --- | --- | --- | --- | --- | --- | --- | --- | --- | --- | --- | --- | --- | --- | --- | --- | --- | --- | --- | --- | --- | --- | --- | --- | --- | --- | --- | --- | --- | --- | --- | --- | --- | --- | --- | --- | --- | --- | --- | --- | --- | --- | --- | --- | --- | --- | --- | --- | --- | --- | --- | --- | --- | --- | --- | --- | --- | --- | --- | --- | --- | --- | --- | --- | --- | --- | --- | --- | --- | --- | --- | --- | --- | --- | --- | --- | --- | --- | --- | --- | --- | --- | --- |

**REFERENCES**

1. Oakley, H. et al. Intraneuronal Beta-Amyloid Aggregates, Neurodegeneration, and Neuron Loss in Transgenic Mice with Five Familial Alzheimer's Disease Mutations: Potential Factors in Amyloid Plaque Formation. *J. Neurosci.* **26**, 10129-10140 (2006).

2. Zheng, K. et al. Curcumin Ameliorates Memory Decline Via Inhibiting Bace1 Expression and Beta-Amyloid Pathology in 5Xfad Transgenic Mice. *Mol. Neurobiol.* **54**, 1967-1977 (2017).

3. Hammers, A. et al. Three-Dimensional Maximum Probability Atlas of the Human Brain, with Particular Reference to the Temporal Lobe. *Hum. Brain Mapp.* **19**, 224-247 (2003).

4. Bullich, S. et al. Optimized Classification of (18)F-Florbetaben Pet Scans as Positive and Negative Using an Suvr Quantitative Approach and Comparison to Visual Assessment. *Neuroimage Clin.* **15**, 325-332 (2017).

5. Shi, K. et al. Bone Marrow Hematopoiesis Drives Multiple Sclerosis Progression. *Cell*. **185**, 2234-2247 (2022).

6. Herisson, F. et al. Direct Vascular Channels Connect Skull Bone Marrow and the Brain Surface Enabling Myeloid Cell Migration. *Nat. Neurosci.* **21**, 1209-1217 (2018).

7. Zheng, Q. et al. Trisomy 21-Induced Dysregulation of Microglial Homeostasis in Alzheimer's Brains is Mediated by Usp25. *Sci. Adv.* **7**, (2021).

8. Vorhees, C. V. & Williams, M. T. Morris Water Maze: Procedures for Assessing Spatial and Related Forms of Learning and Memory. *Nat. Protoc.* **1**, 848-858 (2006).

9. Zhong, L. et al. Soluble Trem2 Ameliorates Pathological Phenotypes by Modulating Microglial Functions in an Alzheimer's Disease Model. *Nat. Commun.* **10**, 1365 (2019).

10. Zhang, J. et al. Aging-Related Changes in Rp3V Kisspeptin Neurons Predate the Reduced Activation of Gnrh Neurons During the Early Reproductive Decline in Female Mice. *Neurobiol. Aging*. **35**, 655-668 (2014).
