# Supplementary material for: Bone marrow B lymphopoiesis accelerates early cerebral amyloid pathology
Source: Signal Transduct Target Ther. 2025 Sep 18;10:312. doi: 10.1038/s41392-025-02419-0 (PMC12443962; doi:10.1038/s41392-025-02419-0)
Supplement: Supplementary file 2 — Revised similarity report [file 41392_2025_2419_MOESM2_ESM.pdf]

rubriq

by Account6775 .

Similarity Index
15%
Similarity by Source
Internet Sources:
9%
Publications:
13%
Student Papers:
2%

Submission date: 22-Aug-2025 08:49PM (UTC+0530)
Submission ID: 2733501980
File name: 21950732.docx (184.37K)
Word count: 6909
Character count: 38162

Title page

Bone marrow B lymphopoiesis accelerates early cerebral amyloid pathology

ABSTRACT

Bone marrow is a major source of hematogenous cells that orchestrate brain immunity. However, alterations in the bone marrow hematopoietic system in patients with Alzheimer's disease (AD) and their potential impacts on neuroinflammation and cerebral β-amyloid (Aβ) pathology remain unknown. Here, we report that Aβ accumulates within the bone marrow of patients with AD and is particularly concentrated in the central nervous system-surrounding bones. In 5×FAD and APP/PS1 mice, two classic mouse AD models, Aβ accumulates within the skull bone marrow prior to substantial cerebral Aβ deposits. Flow cytometry and cell tracking analyses demonstrated that these AD mice exhibit enhanced bone marrow hematopoiesis in B lymphoid lineages, specifically an increase in age-associated B cells (ABCs), accompanied by heightened output of these cells into the brain parenchyma. Furthermore, intracranial Aβ injection into IL-6 knockout mice revealed that Aβ promotes B lymphocyte generation, particularly ABCs, via IL-6 signaling. Single-cell sequencing analysis following intracerebroventricular ABCs injection, combined with *in vitro* microglial culture studies, demonstrated that bone marrow-derived ABCs directly augment microglial reactivity, ultimately exacerbating Aβ neuropathology and cognitive deficits in AD models. Notably, blockade of IL-6R restricts B-cell activity and ABCs in the bone marrow, delays cerebral Aβ pathology and improves cognition. Our findings reveal the potential involvement of bone marrow-derived B cells in the early cerebral amyloid pathology in two mouse AD models and suggest that these B cells may serve as potential therapeutic candidates for patients with AD.

KEYWORDS Alzheimer's disease, beta-amyloid, B lymphocyte, microglia, IL-6

INTRODUCTION

Amyloid plaques feature prominently in Alzheimer's disease (AD). As an essential indicator of AD diagnosis, Aβ starts to aggregate insidiously about 20 years before the presence of any obvious symptoms [1]. During this long interval, neuroinflammation is a key driving factor for the AD progression [2], which can be exacerbated by nearly half of the AD risk genes (APOE, TREM2, CD33, etc.) that are implicated in immune processes [3,4], increased levels of complement molecules and inflammatory mediators [5], and the reactive gliosis and infiltration of immune cells in the brain. Recent studies document microglia as the primary contributors to neuroinflammation in the AD brain, and suggest that their activation status regulates the pathological progression of AD [5,6]. In addition to the resident microglia in the brain[7-10], the peripheral immune system, encompassing innate and acquired immune cells, has been found to be involved in the AD pathogenesis[11,12].

Previous studies have revealed the detrimental effects of peripheral-derived myeloid cells (e.g., neutrophils and monocytes) on the AD progression [13,14]. Recent studies reported an increase of T cells in AD mouse models [15] and in the leptomeninges, CSF, and hippocampus of patients with AD [16]. Moreover, T cell infiltration in AD brains is linked with tau pathology, but not with amyloid pathology, and tau aggregation is considered to be an advanced stage of AD progression [17-19]. Wei et al. explored the beneficial role of $CD8^+$ T cells in AD mice [20]. The involvement of B cells in AD has been reported less extensively than other immune components. Patients with AD demonstrate elevated systemic autoantibodies and an increased population of antibody-secreting B cells in peripheral blood [21]. Matteo et al. documented that individuals with moderate-to-severe AD report a decrease in peripheral $CD19^+$ B cell count and a remodeling of B cell subsets. This remodeling is characterized by an increase of

double-negative (IgD^-CD27^-) memory B cells, a marked reduction in naïve B cells (IgD^+CD27^-), and an upregulation of receptors for pro-inflammatory cytokines [22,23]. Within the AD brain, B lymphocytes are found to be adjacent to Aβ plaques [24] and IgG-immunopositive neurons [25]. Although the above evidence suggests that the peripheral immune system is implicated in AD pathogenesis, much remains to be desired with regards to the origin and types of peripheral immune cells and the relevant mechanisms in the early stage of AD.

Under physiological conditions, the integrity of the brain barrier system—encompassing the meninges, blood-cerebrospinal fluid barrier, and blood-brain barrier (BBB)—limits the presence of peripheral immune cells within the brain parenchyma to very low levels, allowing their participation in brain development and functional homeostasis. Conversely, in the late stages of AD, vascular inflammation, BBB dysfunction, and impaired meningeal lymphatic drainage collectively facilitate substantial infiltration of peripheral immune cells. However, it remains unresolved regarding the origin of peripheral immune cells and the operative mechanism by which they enter the brain parenchyma during the early stages of AD. As a critical immune organ within the peripheral compartment, the bone marrow (BM) is the major site of hematopoiesis, where hematopoietic stem and progenitor cells (HSPCs) produce immune cells that orchestrate immune homeostasis in both the periphery and the central nervous system (CNS) [26-32]. Recent evidence has shown that there is a direct short-vessel connection between the cranial bone marrow and the meninges, through which the skull BM supplies myeloid cells and B cells to the meninges and CNS parenchyma to monitor brain homeostasis [26-29]. Utilizing TSPO-PET imaging, Kolabas et al. detected elevated myeloid cell activation within the skull bone marrow of AD patients [27]. Consequently, the skull bone marrow serves as a primary source of brain-surveilling immune cells, reflecting neuroinflammatory responses in various neurological

disorders. Although few studies have reported the presence of abnormal bone marrow hematopoiesis in AD model mice [30,33,34], several key questions need to be answered in order to understand how bone marrow in the peripheral compartment communicates with the brain to orchestrate neuroinflammation.

In this study, we uncover skull bone marrow-derived B-cell that accelerates early AD pathogenesis. We detected Aβ deposition in the skull BM of AD patients and young AD model mice. Aβ promotes the differentiation and proliferation of HSCs and B lymphocytes, especially age-associated B cells (ABCs), in the bone marrow via IL-6. Notably, *in vivo* tracing of bone marrow cells revealed that ABCs from skull BM can infiltrate the brain parenchyma and lead to accelerated Aβ deposition. Furthermore, blocking IL-6R suppresses B cells and ABCs in the bone marrow, delays cerebral Aβ pathology and improves cognition. These findings suggest that bone marrow-derived B cells are potential therapeutic targets for patients with AD.

RESULTS

Aβ accumulates in the bone marrow of patients with AD and AD model mice.

The aggregation of Aβ in the brain is a major neuropathologic hallmark of AD, but whether Aβ deposits in peripheral immune organs such as the bone marrow remains unknown in patients with AD. For this purpose, Aβ accumulation was assessed via the regional cortical tracer uptake (RCTU) system by positron emission tomography/computed tomography (PET/CT)[35,36]. A total of 26 dementia patients were included, comprising 8 non-AD dementia patients with negative brain Aβ-PET scans and 18 AD dementia patients with positive brain Aβ-PET scans (**supplementary Table 1**). In addition to Aβ deposition in the brain parenchyma, Aβ accumulation in the skull bones and other peripheral bones was detected in patients with AD dementia compared with non-AD dementia patients (**Fig. 1a**).

Analysis of tracer uptake revealed that the standardized uptake value ratio (SUVr) in the skull of patients with AD dementia was markedly greater than that in non-AD dementia patients (**Fig. 1b**). Interestingly, Aβ deposits were also found in the bone marrow of flat bones, such as the ilium, in AD dementia patients but not in the bone marrow of long bones (e.g., the femur) in any patient group. When SUV values in skull subregions were analyzed according to corresponding cerebral cortex regions, significant increases were found in the frontal, temporal, and occipital skulls of patients with AD dementia compared with those with non-AD dementia, whereas the parietal skull showed no significant difference (**Fig. 1c**). These results demonstrate that Aβ is deposited within the skull bone marrow of AD dementia patients.

To test whether Aβ deposits in the bone marrow also exist in mouse models of AD, we quantified $A\beta_{42}$ levels in the skull and femur BM of 2.5-month-old and 5-month-old 5×FAD mice via a high-sensitivity ELISA kit. We found that the level of $A\beta_{42}$ in the skull BM supernatant was higher in 2.5-month-old 5×FAD mice than in wild-type (WT) mice and increased further in the skull BM supernatant at the age of 5 months (**Fig. 1d**). In contrast, no marked increase in $A\beta_{42}$ was evident in the femoral BM of 5×FAD mice. Similarly, $A\beta_{42}$ was deposited in the skull bone marrow of 8-month-old APP/PS1 mice but not in the femoral bone marrow (**Fig. 1e**). The immunohistochemical results also revealed Aβ accumulation in the skull BM of 5-month-old 5×FAD mice (**Fig. 1f**). These results demonstrate that Aβ deposition occurs in the skull BM prior to massive plaque accumulation within the brain parenchyma during the early AD stage.

Proliferation and differentiation of skull bone marrow HSCs and B lymphopoiesis in the early AD stage.

To assess the potential alterations in bone marrow hematopoietic cell lineages in AD, we performed flow cytometric analysis of bone marrow HSCs, immune progenitor cells and mature immune cells

from 3-month-old 5×FAD mice and WT mice. The results showed a remarkable increase of long-term HSCs (Lin$^-$ c-kit$^+$ sca-1$^+$ CD34$^-$ FLK2$^-$ CD48$^-$ CD150$^+$) in the skull BM (**Fig. 2a**), Ki67$^+$ HSCs in 5×FAD mice (**Fig. 2b**), and skull BM lymphoid progenitor cells (CLPs) (**Fig. 2c**). Notably, there was a robust increase of B lymphocytes, not CD4$^+$ T cells or CD8$^+$ T cells, in the skull BM (**Fig. 2d, supplementary Fig. 1a**). Whereas, the number of monocyte–dendritic progenitor (MDP) cells, common myeloid progenitors (CMPs), and granulocyte–monocyte progenitors (GMPs) was not significantly altered (**Fig. 2e, supplementary Fig. 1b**). Moreover, except for Ly6C^{high} monocytes, no apparent discrepancy in the number of neutrophils or Ly6C^{low} monocytes was evident between 5×FAD mice and WT mice (**Fig. 2f, supplementary Fig. 1c**). In addition, we detected increases in HSCs, Ki67$^+$ HSCs, CLPs and MDPs within femur BM (**Fig. 2a-c and 2e**). However, there were few changes in the number of other immune progenitor cells and mature immune cells within the femur BM (**Fig. 2d and 2f**). Overall, in 3-month-old 5×FAD mice, we observed an increase of both HSC counts and activity in the skull and femur BM but only an increase of B lymphopoiesis in the skull BM. In 8-month-old APP/PS1 mice, both HSCs and proliferative HSCs were also elevated in the BM (**supplementary Fig. 2a-b and 2e-f**), with a marked increase of CLPs only in the skull BM (**supplementary Fig. 2c, 2g**).

To further characterize the dynamic alterations in the bone marrow hematopoietic system, we also performed flow cytometry analysis of bone marrow from 10-month-old 5×FAD mice and WT mice. We found a sustained increase in HSC count and activity (**supplementary Fig. 3a-b**), together with a dramatic increase in B lymphopoiesis in both skull BM and femur BM (**supplementary Fig. 3c-d**). These results highlight an enhanced differentiation of HSCs to the lymphoid lineage, particularly B cells in the BM of AD mice, early only in the skull BM and late in the femoral BM.

Bone marrow-derived B lymphocytes infiltrate the brain parenchyma in the early AD stage.

To test whether B cells from skull BM infiltrate the brain parenchyma in 3-month-old 5×FAD mice, we injected APC- or FITC-labeled tracers into the cavities of the skull and femur, respectively, to trace skull- or femur-derived cells by flow cytometry 24 hours or 96 hours after injection (**Fig. 3a**). Consistent with our speculation, the results showed the presence of APC-positive B cells in the brain parenchyma of 3-month-old 5×FAD mice at both 24 hours and 96 hours. Importantly, there were no FITC-positive B cells in the brain parenchyma at any time point (**Fig. 3b**). These results evidence that newly generated B cells can be mobilized from skull BM and penetrate the brain parenchyma.

Next, we further investigated whether the production of B cells from the skull BM increases B cells in the brain. We calculated the number of peripheral immune cells in the brain parenchyma of young AD mice. Indeed, there was an increase of B cells in the brain tissue of 3-month-old 5×FAD mice (**Fig. 3c**), but no marked changes in the number of $CD8^+$ T cells, $CD4^+$ T cells, neutrophils, $Ly6C^{high}$ monocytes, or $Ly6C^{low}$ monocytes, between 5×FAD mice and WT counterparts (**Fig. 3d**). Similarly, an increase of B cells was predominant in the brains of 8-month-old APP/PS1 mice (**supplementary Fig. 2h**). Notably, we detected a sustained and dramatic increase in B cells in the brains of 10-month-old 5×FAD mice (**supplementary Fig. 4a**). In addition, we detected similar numbers of B cells in the spleens of 3-month-old (**supplementary Fig. 4c**) and 10-month-old (**supplementary Fig. 4d**) 5×FAD and WT mice. These data demonstrate sustained B-cell output from the bone marrow to the brain parenchyma in AD mice, indicating the contribution of these cells to Aβ pathology.

Bone marrow-derived age-associated B cells infiltrate the brain parenchyma in the early AD stage.

Furthermore, we analyzed the major subclusters of B cells in the skull BM via flow cytometry. Compared with littermate WT mice, 3-month-old 5×FAD mice presented a significantly greater frequency and number of ABCs (CD45$^+$ CD19$^+$ CD11b$^+$ CD11c$^+$ CD21$^-$ CD23$^-$) and no significant differences in CD138$^+$B, B1a (CD11b$^+$ CD5$^+$), B1b (CD11b$^+$ CD5$^-$), B2 (CD11b$^-$ CD5$^-$), CD21$^-$ CD23$^-$ B, CD21$^+$ CD23$^-$ B and FOB (CD21$^+$ CD23$^+$) (**Fig. 3e**) (**supplementary Fig. 1d**). Consistently, an increase in ABCs was also present in the skull BM of 8-month-old APP/PS1 mice (**supplementary Fig. 2d**). Bone marrow tracing further revealed that skull BM-derived ABCs could be exported to the brain parenchyma in 3-month-old 5×FAD mice at either 24 hours or 96 hours (**Fig. 3f**). Furthermore, the number and percentage of ABCs in the brain parenchyma of both 3-month-old 5×FAD mice (**Fig. 3g**) and 8-month-old APP/PS1 mice (**supplementary Fig. 2j**) were significantly increased, as determined via flow cytometry.

Next, consistent with the previous report [37], we also found that ABCs highly express the transcription factor Zeb2 (**supplementary Fig. 5a-b**). Immunofluorescence staining revealed that ABCs were predominantly located in the perivascular and periventricular regions of the brain parenchyma in 5×FAD mice (**supplementary Fig. 5c**). To characterize the features of ABCs, we further performed bulk RNA-sequencing analysis of bone marrow-derived ABCs (**Fig. 3h**). Differential gene expression analysis between WT and 5×FAD (fold change > 2.0; FDR < 0.05) revealed 410 differentially expressed genes. Notably, compared with WT mice, the 5×FAD mice reported an increase in TLR7, IRF7, IRF9, and Vpreb1/2, which were previously reported to be associated with ABC production [38],

and IL33, Lgals9, Cfb, and Tnfsf10, which are reportedly associated with the dysfunction of myeloid cells [39,40]. However, Nr4a1, the gatekeeper of B-cell immune tolerance, was decreased in 5×FAD mice [41]. Pathway analysis confirmed that cell senescence, primary immunodeficiency, the B-cell receptor signaling pathway and immunoprotein production were among the highly impacted pathways in the context of AD. These results suggest that the differentiation and output to the brain parenchyma of the skull BM B lymphoid lineage, especially ABCs, are increased in the early AD stage.

Aβ deposition increases the number of age-associated B cells in the BM via IL-6.

Since Aβ is deposited in the skull cavity both in patients with AD and in AD model mice, we hypothesized that Aβ directly promotes the proliferation and differentiation of HSCs and increases B lymphopoiesis and ABC production in AD mice. To address this issue, $A\beta_{42}$ or scrambled $A\beta_{42}$ was first injected into the skull cavities of WT mice and then the skull BM underwent the flow cytometric analysis at 1 month after injection (**Fig. 4a**). Surprisingly, $A\beta_{42}$ injection significantly increased the number and frequency of HSCs, CLPs (**Fig. 4b**), and ABCs (**Fig. 4d**). However, the counts and frequencies of CMPs, GMPs, and MDPs were not significantly altered (**Fig. 4c**). Second, to determine the underlying mechanisms of increased B lymphopoiesis and ABC production in the skull BM of AD mice, we quantified immune factors from the skull BM and found that IL-6 level was greatly increased in 3-month-old 5×FAD mice (**Fig. 4e**) while that of other cytokines, such as IL-2, IL-4, IL-5, IL-10, TNF-α and IFN-γ, did not differ. Consistently, $A\beta_{42}$ injection increased not only IL-6 level in the supernatants and precipitates of skull BM (**Fig. 4f**) but also the number and frequency of IL-6-positive cells in the skull BM of WT mice after 7 days of injection (**Fig. 4a, 4g**). These results indicate that Aβ

not only increases IL-6 levels but also directly promotes the proliferation and differentiation of HSCs, the differentiation of B lymphocytes and the upregulation of ABCs in the bone marrow.

To determine the full and essential role of IL-6 in Aβ-mediated B lymphopoiesis and ABC production, first, IL-6 was injected into the BM cavity of WT mice, and flow cytometric analysis revealed that IL-6 increased the number and frequency of B cells and ABCs (**Fig. 4h**). Furthermore, $Aβ_{42}$ was injected into the BM cavities of WT mice and IL-6 knockout mice and then the BM underwent flow cytometry at 1 month after injection. The results revealed that the number and frequency of B cells and ABCs were markedly lower in IL-6 KO mice than in WT counterparts (**Fig. 4i**). These data evidence that Aβ contributes to the differentiation and proliferation of B lymphocytes and the increase of ABCs in skull BM through IL-6 in the early AD stage.

Bone marrow-derived ABCs accelerate early Aβ pathology and augment microglial reactivity in 5×FAD mice.

To test the impact of ABC infiltration into the brain parenchyma on Aβ pathology, we sorted bone marrow-derived ABCs ($CD19^+$ $CD11c^+$) from 10-month-old 5×FAD mice and injected them into the bilateral ventricles of 3-month-old 5×FAD mice once a month until 5 months of age. $CD19^+$ $CD11c^+$ B cells derived from the BM of WT mice were used as controls (**Fig. 5a**). Compared with that of the control counterpart, cognitive impairment of the ABC group was greatly aggravated, as evidenced by the decrease in spatial learning and memory performance of 5×FAD mice in both the training trial (**Fig. 5b**) and probe trial (**Fig. 5c**) of the Morris water maze (MWM) task. ABCs also obviously impaired performance in the Y maze (**Fig. 5d**). Furthermore, thioflavin S (TS) staining was performed to examine Aβ plaques in brain sections from FAD-control and FAD-ABC mice to investigate whether

ABC injections impact Aβ pathology in AD mice. Indeed, ABC injections significantly increased the Aβ plaque content in the hippocampus and cortex (**Fig. 5f-g**). Consistent with the TS staining results, ELISA analysis also revealed that the level of TBST-soluble $A\beta_{42}$ was significantly greater in the hippocampus of FAD-ABC mice than in that of FAD-control counterparts (**Fig. 5e**). Microglia are central players in neuroinflammation that facilitate Aβ production and promote the deposition of Aβ plaques [42-46]. We measured microglial activity after the injection of ABCs and found that ABC injection augmented reactive microgliosis and reduced the complexity of the microglial branches in 5×FAD mice (**Fig. 5h-i**). These findings demonstrate that the infiltration of ABCs into the brain accelerates the pathological process of AD.

To decipher specific microglial states associated with the ABCs that infiltrate the brain, single $CD11b^+$ cells were sorted from the cortex and hippocampus of FAD-control and FAD-ABCs, and single-cell transcriptomes were obtained via the 10x Genomics platform (**supplementary Fig. 6a**). A total of 37146 single cells were visualized on UMAP dimensions. The unsupervised clustering reported 8 distinct cell types that were annotated on the basis of cell type-specific markers (**supplementary Fig. 6b-c**). Among microglia, eleven clusters, including homeostatic (HM), disease-associated (DAM), inflammatory, interferon-responsive (IFN-R), MHC-II, transition and proliferating microglia, were identified (**Fig. 5j and supplementary Fig. 6d**). The proportion of the HM cluster was lower in the FAD-ABC mice, whereas the proportion of the DAM, inflammation and IFN-R clusters was greater in the FAD-ABC mice (**Fig. 5k**). Pseudotime analysis revealed that the HM cluster of microglia may differentiate into DAMs in FAD-ABC mice (**Fig. 5l**). Consistently, the microglia in the FAD-control mice presented an increase in homeostatic marker genes (Cx3cr1, P2ry12 and Tmem119). In contrast, the expression of disease-related marker genes (Trem2, Apoe, Lyz2, Cst7, Lpl, Axl, Itgax and Spp1)

was increased in FAD-ABC mice (**supplementary Fig. 6e-f**). Further immunofluorescence analysis confirmed the decrease in the homeostatic marker P2ry12 and increase in the disease-related marker Cd11c in FAD-ABC mice (**supplementary Fig. 6g**). GO term analysis revealed that response to interferon-gamma, antigen processing and presentation and positive regulation of the adaptive immune response were among the top altered pathways of DAM in the FAD-ABC group versus the FAD-control group (**Fig. 5m**). These data demonstrate that at the transcriptional level, ABCs can enhance the maladaptive response of microglia.

To investigate ABC–microglia crosstalk, microglia were treated with conditioned media (CM) from ABCs derived from 5×FAD mice and WT counterparts (**supplementary Fig. 7a**). The results showed that 5×FAD-derived ABCs increased IL-6 levels (**supplementary Fig. 7b**) and impaired microglial $A\beta_{42}$-555 phagocytosis (**supplementary Fig. 7c-d**). Similarly, intracerebroventricular ABC administration to 10-month-old APP/PS1 mice markedly exacerbated the cerebral amyloid burden and maladaptive microglial activation (**supplementary Fig. 7e-h**). The results suggest that ABCs can augment microglial reactivity and switch on their detrimental effects related to neurodegeneration [7,9,47].

Collectively, these data indicate that the infiltration of ABCs into the brain parenchyma aggravates Aβ pathology and enhances microglial reactivity in the early AD stage.

Blockade of IL-6 signaling reduces the number of bone marrow-derived ABCs, cerebral Aβ pathology and cognitive impairments

To further investigate the role of IL-6 signaling in bone marrow B-cell production in AD mice, the 3-month-old 5×FAD mice received an intraperitoneal injection of an FDA-approved monoclonal antibody (tocilizumab) that blocks the IL-6 receptor (**Fig. 6a**). The results showed that tocilizumab

treatment markedly reduced counts and frequencies of B cells and ABCs in the skull BM (**Fig. 6b-c**) and the number of ABCs in the brain parenchyma of AD mice (**Fig. 6d**). These data evidence that IL-6 signaling plays a key role in bone marrow B lymphopoiesis and the output of ABCs into the brain parenchyma in AD mice.

Next, to explore whether the inhibition of IL-6 signaling confers protection against early AD, we collected brain tissue to measure Aβ deposition. Tocilizumab decreased Aβ deposition in the hippocampal DG and cortex of 5×FAD mice (**Fig. 6e-f**). Guanidine-soluble $Aβ_{42}$ levels also greatly declined in the hippocampus of 5×FAD mice (**Fig. 6g**). Moreover, reactive gliosis and a reduction in the complexity of the microglial branches were improved in 5×FAD mice receiving tocilizumab (**Fig. 6h**). Immunofluorescence analysis confirmed the increase in the homeostatic marker P2ry12 and the decrease in the disease-related marker Cd11c in the 5×FAD-tocilizumab group (**Fig. 6i**). These results indicate that decreasing the number of BM-derived ABCs by blocking IL-6R can restrain early brain Aβ pathology and microglial reactivity.

Since the increase in the differentiation of HSCs to the B lymphoid lineage persisted in the bone marrow of AD mice (**Fig. 2, supplementary Fig. 3 and supplementary Fig. 4**), we investigated whether blocking BM B cells would similarly benefit AD mice at intermediate and advanced stages. We intraperitoneally injected 5-month-old 5×FAD mice with tocilizumab every two weeks until 8 months, after which the behavioral tests were performed (**supplementary Fig. 8a**). Tocilizumab obviously enhanced the performance of the mice in the Y maze (**supplementary Fig. 8b**). Moreover, tocilizumab treatment significantly improved spatial cognitive impairment, as indicated in the improved learning and memory performance of 5×FAD mice in both the training trial (**supplementary Fig. 8c**) and probe trial (**supplementary Fig. 8d**) of the MWM task. Notably, the

low-dose and long-term tocilizumab intervention markedly decreased the number of ABCs in the skull BM and brain parenchyma of 5×FAD mice (**supplementary Fig. 8e-f**). Similarly, tocilizumab diminished the Aβ deposition in the hippocampus and cortex of 5×FAD mice (**supplementary Fig. 8g-i**). Moreover, gliosis and the complexity of the microglial branches were improved in 5×FAD mice receiving tocilizumab (**supplementary Fig. 8j-k**). To investigate the long-term effects of IL-6R blockade on AD pathology, 6-month-old 5×FAD mice received intraperitoneal injections (tocilizumab or control IgG) for one month, followed by a 1-month drug washout period. The results demonstrated that even after washout, the tocilizumab-treated group still presented a significantly reduced Aβ burden and attenuated aberrant microglial activation compared with the IgG-treated control group (**supplementary Fig. 9**).

Altogether, these data demonstrate that the blockade of IL-6 signaling can reduce the number of bone marrow-derived ABCs, restrain cerebral Aβ pathology and decrease microglial reactivity during AD progression.

DISCUSSION

This study revealed that there was a current under-recognition of BM as a source of B cells governing Aβ pathology in the early stage of AD. PET/CT scanning unexpectedly revealed Aβ deposits within the skull BM of AD patients. Surprisingly, we detected Aβ deposition in the skull BM but not in the femoral BM of young 5×FAD mice or APP/PS1 mice. Importantly, Aβ promotes the proliferation and differentiation of HSCs and B lymphopoiesis in the BM via IL-6 signaling. Notably, *in vivo* tracing of BM cells revealed that B lymphocytes, particularly ABCs from skull BM, can infiltrate the brain parenchyma and lead to accelerated Aβ deposition and microglial reactivity. Importantly, blocking IL-

6R suppresses bone marrow B lymphopoiesis, cerebral Aβ pathology, microglial reactivity and cognitive impairments.

Aβ accumulation occurs naturally during the aging process [48-50]. In the present study, Aβ accumulated in the skull BM of patients with AD and early AD mice (**Fig. 1**). Interestingly, Aβ deposition is preferentially found in the subregion of the skull, which corresponds to the frontal lobe, temporal lobe and occipital lobe susceptible to AD [51,52]. Importantly, we demonstrated that Aβ deposition enhances bone marrow B lymphopoiesis, particularly that of ABCs, in AD model mice (**Fig. 4**). Combined with *in vivo* cell labeling in the skull and femur [53] and flow cytometry analysis, we demonstrated that in the early AD stage in model mice, compared with cells in the femur BM, skull BM-derived B cells were more likely to migrate to adjacent brain tissue (**Fig. 3b, 3f**). Nevertheless, femur BM may also be involved in brain B-cell infiltration in late-stage AD mice. Our findings revealed that the CNS adjacent to skull bone marrow can sense the environment earlier, and B cells represent the "first responders" in the context of AD. Recent studies describe the calvaria as a source of meningeal myeloid cells [53] and B cells [54], and indicate that, under homeostasis and inflammation, skull BM provides the CNS with myeloid cells[29,54.] We further integrate these findings and reveal that these immune cells are involved in the typical neurodegenerative disease AD.

We found that B cells enter the brain, particularly ABCs, and exacerbate Aβ deposition and microglial reactivity (**Fig. 5**), which is in line with the previous findings that B cells are detrimental to AD [55]. Instead, the bright spot in our work was that B cells infiltrating the brain parenchyma from the CNS adjacent to the skull bone marrow, not from the circulation. These findings highlight the importance of B cells within skull bone marrow during the initiation and progression of AD. ABCs can produce antibodies and display antigen-presenting and proinflammatory abilities, which are

critical components of the immune response and increasingly associated with the pathogenesis of aging-related diseases, autoimmune disorders, and infections [56]. We show that bone marrow-derived ABCs from AD mice overexpress interferon-stimulated genes, cytokines, adhesion molecules, etc., which likely confers greater damage to the AD brain. Consistently, single-cell transcriptomic analysis of microglia revealed that ABCs accelerate the transition from HMs to DAMs, inflammation, and IFN-R subclusters, and enhance neurodegeneration-associated microglial activity [7,9,47]. Further cellular experiments demonstrated that AD mouse-derived ABCs promoted inflammatory factor expression in microglia and impaired their phagocytosis of Aβ (**supplementary Fig. 7a-d**). However, many issues remain to be addressed, such as the possible interaction molecules between ABCs and microglia and the effects of ABC infiltration into the brain on chemotaxis and the activation of peripheral T cells.

The literature indicates that the bone marrow microenvironment is critical for HSC aging [57]. The senescence of the bone marrow hematopoietic niche drives a shift from asymmetric to symmetric division in HSCs and impairs their self-renewal capacity due to a decrease in key niche factors such as IGF1, Cxcl12, IL7, SCF, and Notch ligands. Moreover, inflammatory mediator-primed accumulation of megakaryocytes (MKs), macrophages (Mφs), plasma cells (PCs), aging-associated B cells, and myeloid-derived suppressor cells (MDSCs) collectively promotes the platelet/myeloid-biased phenotype in HSCs through the secretion of inflammatory cytokines, including CCL5, TNF-α, IL-1β, IFN-γ, Wnt5a, and TGFβ. Our findings demonstrate that Aβ induces HSC proliferation and differentiation and B lymphopoiesis through IL-6 signaling. Bone marrow hematopoiesis is controlled by BM niche cell-derived molecules, including growth factors, chemokines and membrane-binding ligands[57]. B lymphopoiesis also relies on the production of supportive signals by BM stromal cells [58,59]; in particular, IL-6 plays a critical role in the differentiation and maturation of B cells [60-62]. In the present

study, we provide evidence that IL-6 signaling is a sufficient and necessary condition for B lymphopoiesis in the bone marrow (**Fig. 4e-i**). Future research urgently requires integration with patient-derived samples to comprehensively elucidate the effects of bone marrow microenvironmental factors on AD-associated hematopoietic dysregulation. Although the cellular components that produce IL-6 in the context of AD await further examination, the priority should focus on B cells as preferential responders so as to appreciate their role in AD development. In addition, where does Aβ in skull BM come from in the early stage? Recent studies revealed that cerebrospinal fluid (CSF) accesses skull BM via dura-skull channels and that CSF-borne cues promote myelopoiesis during spinal cord injury [28] and bacterial meningitis [57]. These findings suggest that future studies need to confirm whether the source of Aβ in skull BM involves CSF in AD.

Our findings have clinical relevance. Given that the bone marrow output of ABCs accelerates Aβ neuropathology and microglial reactivity, we propose that the detection of B-cell levels in CSF holds promise as a marker for the early diagnosis of clinical AD. Given that the changes in B cells within the bone marrow during the early AD stages in mice are dominated by an increase in ABCs (**Fig. 3e**) and that IL-6 is essential for the Aβ-induced upregulation of ABCs (**Fig. 4h-i**), we treated AD model mice with the IL-6R inhibitor tocilizumab rather than the traditional approach of depleting all B cells with an anti-CD20 antibody. Unsurprisingly, we found that tocilizumab treatment reduced ABCs in the bone marrow and brain of AD mice, restrained brain Aβ pathology, regulated microglial activation, and improved cognitive function in both the early (**Fig. 6**) and late stages (**supplementary Fig. 8-9**). However, Rishi et al. did not find any association between the risk of AD and that of related dementia in patients with rheumatoid arthritis treated with tocilizumab compared with abatacept (a T-cell activation inhibitor)[63]. Our findings indicate that targeting bone marrow B cells with tocilizumab may

deserve future investigations in patients with AD. As such, we propose that targeting bone marrow B cells can reduce early cerebral Aβ pathology and perhaps benefit patients with AD. Given that the IL-6 signaling pathway is involved not only in immune regulation but also in broader physiological processes, long-term tocilizumab use requires careful monitoring for hematologic, metabolic, and immune-related off-target effects, balancing therapeutic benefits against systemic physiological disruptions.

Our study revealed that bone marrow-derived B cells accelerate Aβ pathology and exacerbate microglial reactivity and that immune therapy targeting bone marrow-derived B cells via IL-6R blockade may benefit early AD patients. There are several issues to be explored in the future. Owing to the complexity of bone marrow stromal cells, we cannot currently identify the major cell type responsible for both the response to Aβ and the production of IL-6. In the present study, we have not yet been able to directly block the IL-6/IL-6R axis in the bone marrow cavity, and for tocilizumab to affect ABC or B cells to attenuate Aβ pathology, this will need to be addressed with bone marrow chimeras of IL-6R-specific deficient B cells. Owing to ethical limitations, determining how the bone marrow of patients with AD changes is difficult, and a transgenic monkey AD model may be able to answer this question. In addition, APOE4 and TREM2, two important risk factors for AD, are highly expressed in peripheral myeloid cells. Further investigations are warranted to test whether APOE4 and TREM2 can accelerate the pathological process of AD involving bone marrow-derived B cells.

MATERIALS AND METHODS

Patients

Patient studies observed the Declaration of Helsinki. The inclusion of human subjects and supporting documentation were granted by the Ethics Committees of The First Affiliated Hospital of Fujian Medical University (Approval No. : MRCTA, ECFAH of FMU [2021]692). All the subjects gave their informed consent at the time of enrollment. PET/CT images were acquired from 8 non-AD dementias (2 males and 6 females) and 18 AD dementias (11 males and 7 females). No marked difference was present in age (66.88± 7.16 vs. 68.47 ±9.61 years, P = 0. 681), education (11.63 ± 5.81 vs. 10.76 ± 5.27 years, P = 0.716) and MMSE (21.63 ± 5.37 vs. 17.65 ± 5.37, P = 0.10) scores of the recruited subjects (**supplementary Table 1**).

Animals

The 5×FAD mice, a model that coexpresses five familial AD mutations in the human amyloid precursor protein [K670N/M671L (Swedish) + I716V (Florida) + V717I (London)] and human presenilin 1 (M146L+L286V) under the control of the murine Thy-1 promoter [64], were provided by Jackson Laboratory (stock no. 034848-JAX, Bar Harbor, ME, USA). APP/PS1 mice (stock no. 034832-JAX), another model that features a mutant human presenilin 1 (PS1-dE9) and a chimeric mouse/human amyloid precursor protein (Mo/HuAPP695swe), and IL-6 knockout mice (B6.129S2-*Il6*^{tm1Kopf}/J) (stock no. 002650) were procured from Jackson Laboratory (Bar Harbor, ME, USA). All the mutant mice were backcrossed to the B6 background for 15 generations. The genotypes were verified via a PCR analysis of the tail DNA as documented previously [65]. Both sexes of mice were enrolled in this study and raised (a maximum of five individuals per cage) in a pathogen-free environment, which was maintained on a controlled light-dark cycle, with a stable temperature of 21 ± 1°C and a humidity level ranging from 50% to 60%. The animals accessed both water and food unrestrictedly. All animal procedures followed the regulations and policies established by the Institutional Animal Care and Use

Committee at Fujian Medical University and adhered to international standards for the ethical treatment of animals.

Single-cell RNA sequencing.

For the intracerebroventricular injection of ABCs, the single-cell sequencing was performed for $CD11b^+$ cells from the forebrain and hippocampus of 5×FAD mice. Single-cell capture was achieved via a 10x Genomics single-cell 3' system. Downstream gene expression matrices were acquired via the function of Cell Ranger with default parameters. Low-quality cells whose gene expression was fewer than 200 or more than 8,000 genes and whose number of mitochondrial genes was > 6% and whose genes were expressed in fewer than 3 cells were excluded from further analysis. After filtering, the remaining 18685 MG cells from the control group and 18461 MG cells from the ABC group were analyzed in this study. The data were then normalized via the function 'NormalizeData' of Seurat. The top 2000 highly variable genes were recruited via the function 'FindVariableFeatures', and then 'ScaleData' was adopted. Principal component (PC) analysis was performed via the 'RunPCA' function of Seurat. The top 20 PCs were used for dimensionality reduction via the 'RunUMAP' function. The cell types were categorized according to the expression of canonical marker genes for each cluster: microglia (Tmem119, Cx3cr1, P2ry12), astrocytes (Gfap, Slc1a2, S100b), BAMs (Mrc1, Ms4a7, Pf4), oligodendrocytes (Mbp, Mog, Olig1), neutrophils (Lcn2, Retnlg, Msrb1), ependymal cells (Foxj1, Ttr, Ak7), endothelial cells (Prom1, Pecam1, Fn1), and T cells (Ccl5, Trbc2, Id2).

After the identification of cell types, the microglial lineage was extracted and subclustered for further analysis. Subclustering was performed via Seurat with the top 11 principal components. The identification of marker genes was accomplished by comparing each cluster with all other clusters via the FindAllMarkers function with default settings (log-fold change threshold of 0.25 and >10% of cells

expressing the gene). The cell clusters from each tissue were annotated on the basis of the expression of marker genes. To further assess the activation of microglia, the Monocle package was used to analyze single-cell trajectories to discover developmental transitions. GO enrichment analysis of the differentially expressed gene sets was processed via the clusterProfiler R package. GO terms with adjusted P values less than 0.05 were deemed significantly enriched with DEGs.

Bone marrow cell dye tracer

The cell tracker was injected into the cranium and femur according to the literature with some modifications [53], the bone marrow cell tracer for leg bones was the FITC channel (CellTracker™ Green CMFDA dye), and the bone marrow cell tracer for cranial bones was the APC channel (CellTracker™ dark red dye). Flow cytometric assays were performed on the tracer cells 24 hours or 96 hours after dye injection. In summary, the procedure for microinjection into the tibial and skull marrow was carried out sequentially under anesthesia with 1.5% isoflurane, utilizing a 5 µl syringe (#65 Hamilton Co., USA) fitted with a custom 34G blunt needle. A midline incision was created in the skin above the skull to reveal both the anterior (near the bregma) and posterior (including the cerebellum) marrow sites. One or two microinjections were administered at each marrow location (specifically the left frontal, right frontal, and occipital sites). Initially, a 33G needle was employed for a careful "predrilling" procedure to avoid damaging the inner skull wall. If the procedures failed at this phase, the animals involved were excluded from the study. Approximately 2–3 µl of a red fluorescent tracker was meticulously injected at each site through the previously drilled openings, taking 20–30 seconds per injection, culminating in a total of 10 µl of the red tracker injected across the skull (spanning four injection sites). The injection process was closely observed under a microscope. Subsequently, the skin was sutured using 6–0 silk thread. For the tibial marrow injection, the skin was

disinfected and incised just below the knee, where the muscle insertion site was gently scraped off the bone at the designated injection point. A 30G needle was utilized to perforate the bone wall, after which the needle from the Hamilton syringe was inserted into the marrow cavity to deliver 3 µl of a green cell tracker over a span of 30 seconds. Finally, the skin was sutured using 6–0 thread.

Bone marrow cavity injection of Aβ or IL-6

Preparation of oligomeric $A\beta_{42}$: Human $A\beta_{42}$ peptide (AS-20276, AnaSpec) was first dissolved in 1,1,1,3,3,3-hexafluoro-2-propanol (HFIP), evaporated in a hood overnight, and then vacuum freeze-dried at 4°C for 1 hour before the dried film was stored at -80°C. After the preparation of oligomeric $A\beta_{42}$, the dried peptide was dissolved in dimethyl sulfoxide (DMSO) to a final concentration of 5 mM. After brief sonication for 1 min in the bath sonicator, a cold phenol-free F-12 cell culture medium was added to a final concentration of 100 µM $A\beta_{42}$. Finally, this mixture was transferred to 4°C and incubated for 24 h to generate oligomeric $A\beta_{42}$.

The cranial injection method involves the use of a 33G needle to first lightly drill the hole, which does not penetrate the skull; then, the injection site at the location of the parietal bone near the sagittal fossa is selected, the 34G needle is slowly inserted into the lumen, and the microliter syringe (Hamilton) is slowly pushed to reduce fluid leakage. Two sites were injected into the skull, and 3 µl of 0.6 µg oligomeric Aβ was injected into each site. Scrambled $A\beta_{42}$ was used as a control.

IL-6 femur marrow injection method: The disinfected skin was incised open up to the knee, and the muscle insertion site was locally scraped off the bone at the chosen site. A pore was created on the bone wall with a 30G needle. The needle of the Hamilton syringe was inserted into the marrow cavity

to inject 2 µl of 100 ng of IL-6 protein (50 ng/µl) into each femur over 30 seconds. The skin was sutured with 6–0 thread.

Tocilizumab administration

Tocilizumab (Roche, Schweiz) was diluted in saline. Briefly, for short-term intervention, we administered tocilizumab (18 mg/kg) or control IgG intraperitoneally to three-month-old mice once every other day for 1 month. For long-term intervention, we administered tocilizumab (8 mg/kg) or control IgG intraperitoneally to five-month-old mice once every 2 weeks for 3 months. The corresponding behavioral experiments were proceeded at the end of the treatment. The animals were divided into IgG-treated and tocilizumab-supplemented groups according to a random number table.

Statistical analysis

Animals were randomly assigned to treatment groups by Excel-generated random numbers. All analyses were processed by investigators blinded to the grouping. All the experiments reported were duplicated at least twice. All the data were described as the means ± SEMs and analyzed with GraphPad Prism 8.0. Data normality was examined by the Shapiro–Wilk test. The homogeneity of variance was assessed by Bartlett's test. For comparisons between two independent, unpaired groups, the data with a normal distribution and homogeneous variances were assessed by the unpaired Student's t test and otherwise by the Mann–Whitney U test. Data from three groups with one variable were compared by One-way analysis of variance (ANOVA) followed by Tukey's post hoc test. Comparisons among multiple groups with two or more variables were analyzed by two-way ANOVA followed by the Bonferroni post hoc correction. MWM analysis was conducted by one-way or multiway repeated-measures ANOVA. The specific statistical methods and statistical parameters are detailed in the figures

and figure legends. Significance was set at $p < 0.05$ and expressed as $*p < 0.05$, and $**p < 0.01$, $p < 0.001$.

Further information is provided in the Supplementary Materials and Methods.

rubriq

ORIGINALITY REPORT

15%	9%	13%	2%
SIMILARITY INDEX	INTERNET SOURCES	PUBLICATIONS	STUDENT PAPERS

PRIMARY SOURCES

1	Fanny Herisson, Vanessa Frodermann, Gabriel Courties, David Rohde et al. "Direct vascular channels connect skull bone marrow and the brain surface enabling myeloid cell migration", Nature Neuroscience, 2018<br>Publication	2%
2	www.ncbi.nlm.nih.gov<br>Internet Source	1%
3	www.researchgate.net<br>Internet Source	1%
4	www.nature.com<br>Internet Source	1%
5	Shin-Bi Oh, Min Sun Kim, SuJi Park, HyunJu Son, Seog-Young Kim, Min-Seon Kim, Dong-Gyu Jo, Eunyoung Tak, Joo-Yong Lee. "Clusterin contributes to early stage of Alzheimer's disease pathogenesis", Brain Pathology, 2018<br>Publication	1%
6	Gabriel Virella. " Medical Immunology", CRC Press, 2019<br>Publication	1%
7	www.mdpi.com<br>Internet Source	<1%
8	dokumen.pub<br>Internet Source	<1%
9	Si Wang, Xiaohong Yao, Shuai Ma, Yifang Ping et al. "A single-cell transcriptomic landscape	<1%

of the lungs of patients with COVID-19", Nature Cell Biology, 2021
Publication

10	Geng-Di Huang, Li-Xin Jiang, Feng Su, Hua-Li Wang, Chen Zhang, Xin Yu. "A novel paradigm for assessing olfactory working memory capacity in mice", Translational Psychiatry, 2020<br>Publication	<1 %
11	Li-Xin Jiang, Geng-Di Huang, Feng Su, Huali Wang, Chen Zhang, Xin Yu. "Vortioxetine administration attenuates cognitive and synaptic deficits in 5×FAD mice", Psychopharmacology, 2020<br>Publication	<1 %
12	assets.researchsquare.com<br>Internet Source	<1 %
13	bmcoralhealth.biomedcentral.com<br>Internet Source	<1 %
14	Xue Mi, Xinglin Ruan, Renyi Lin, Shuxin Huang, Ping Cai, Xiaochun Chen, Jiangfeng Liao, Xiaoman Dai. "Intranasal administration of Ganoderma lucidum-derived exosome-like nanovesicles ameliorates cognitive impairment by reducing inflammation in a mouse model of Alzheimer's disease", Frontiers in Pharmacology, 2025<br>Publication	<1 %
15	Nicole C. Walsh. "Rheumatic diseases: the effects of inflammation on bone", Immunological Reviews, 12/2005<br>Publication	<1 %
16	elifesciences.org<br>Internet Source	<1 %
17	www.spandidos-publications.com<br>Internet Source	

<1%

18	Shunta Sakaguchi, Sonoko Mizuno, Yasushi Okochi, Chiharu Tanegashima et al. "Single-cell transcriptome atlas of Drosophila gastrula 2.0", Cell Reports, 2023<br>Publication	<1%
19	pmc.ncbi.nlm.nih.gov<br>Internet Source	<1%
20	www.ijbs.com<br>Internet Source	<1%
21	www.science.gov<br>Internet Source	<1%
22	Justin Rustenhoven, Jonathan Kipnis. "Brain borders at the central stage of neuroimmunology", Nature, 2022<br>Publication	<1%
23	Ki Kim, Xin Wang, Emeline Ragonnaud, Monica Bodogai et al. "Therapeutic B-cell depletion reverses progression of Alzheimer's disease", Nature Communications, 2021<br>Publication	<1%
24	Nidhi Puranik, Shiv Kumar Yadav. "Nanomedicine in the Treatment and Management of Alzheimer's Disease", CRC Press, 2025<br>Publication	<1%
25	Se Hoon Choi, Enjana Bylykbashi, Zena K. Chatila, Star W. Lee et al. "Combined adult neurogenesis and BDNF mimic exercise effects on cognition in an Alzheimer's mouse model", Science, 2018<br>Publication	<1%
26	Giulia Castellani, Tommaso Croese, Javier M. Peralta Ramos, Michal Schwartz.	<1%

"Transforming the understanding of brain immunity", Science, 2023
Publication

27 molecularneurodegeneration.biomedcentral.com <1 %
Internet Source

28 Qingtao Sun, Jianping Zhang, Anan Li, Mei Yao, Guangcai Liu, Siqi Chen, Yue Luo, Zhi Wang, Hui Gong, Xiangning Li, Qingming Luo. "Acetylcholine deficiency disrupts extratelencephalic projection neurons in the prefrontal cortex in a mouse model of Alzheimer's disease", Nature Communications, 2022 <1 %
Publication

29 keep.lib.asu.edu <1 %
Internet Source

30 Adam O. Ghoweri, Lara Ouillette, Hilaree N. Frazier, Katie L. Anderson et al. "Electrophysiological and Imaging Calcium Biomarkers of Aging in Male and Female 5×FAD Mice", Journal of Alzheimer's Disease, 2020 <1 %
Publication

31 Daniel L Aldridge, Zachary Lanzar, Anthony T Phan, David A Christian, Ryan Pardy, Booki Min, Ross M Kedl, Christopher A Hunter. "IL-27 limits HSPC differentiation during infection and protects from stem cell exhaustion", eLife Sciences Publications, Ltd, 2025 <1 %
Publication

32 Jose A. Mazzitelli, Leon C. D. Smyth, Kevin A. Cross, Taitea Dykstra et al. "Cerebrospinal fluid regulates skull bone marrow niches via direct access through dural channels", Nature Neuroscience, 2022 <1 %
Publication

33	aseducationbook.hematologylibrary.org<br>Internet Source	<1 %
34	downloads.hindawi.com<br>Internet Source	<1 %
35	www.scitechnol.com<br>Internet Source	<1 %
36	Hyun-Seok Hong. "Inhibition of Alzheimer's amyloid toxicity with a tricyclic pyrone molecule *in vitro* and *in vivo*", Journal of Neurochemistry, 02/2009<br>Publication	<1 %
37	hdl.handle.net<br>Internet Source	<1 %
38	pubmed.ncbi.nlm.nih.gov<br>Internet Source	<1 %
39	www.bdbiosciences.com<br>Internet Source	<1 %
40	Dilip Ghosh, Benny Antony. "Ashwagandha - Potential Drug Candidate from Ancient Ayurvedic Remedy", CRC Press, 2025<br>Publication	<1 %
41	Gaia Faustini, Margherita Tassan Mazzocco, Albert Comelli, Alberto Brugnoli et al. "Alpha-synuclein pathological deposition alters functional homeostasis and connectivity of striatal dopaminergic synapses and results in microglia activation, pathology spreading, muscle stiffness, whole brain atrophy and metabolic changes before producing frank nigral neuron deafferentation", Springer Science and Business Media LLC, 2025<br>Publication	<1 %
42	Kunmu Zheng, Xiaoman Dai, Nai'an Xiao, Xilin Wu, Zhen Wei, Wenting Fang, Yuangui Zhu,	<1 %

Jing Zhang, Xiaochun Chen. "Curcumin Ameliorates Memory Decline via Inhibiting BACE1 Expression and β-Amyloid Pathology in 5×FAD Transgenic Mice", Molecular Neurobiology, 2016
Publication

43	Xiaoqin Zhang, Yufei Mei, Yang He, Dongpi Wang et al. "Ablating Adult Neural Stem Cells Improves Synaptic and Cognitive Functions in Alzheimer Models", Stem Cell Reports, 2021<br>Publication	<1 %
44	Yimiao Wang, Ze Wang, Yue Li, Min Cao, Shuying Zhang, Shixin Ding, Sijia Chen, Yuxi Jin, Yanli Zhang, Junying Gao, Ming Xiao. "Arhgef7 as a key target for enriched environment rescuing spatial cognitive deficits and anxiety-like behaviors in a mouse model of Alzheimer's disease following early social isolation", Alzheimer's Research & Therapy, 2025<br>Publication	<1 %
45	Yue Xu, Kai Jiang, Feng Chen, Jie Qian et al. "Bone marrow-derived naïve B lymphocytes improve heart function after myocardial infarction: a novel cardioprotective mechanism for empagliflozin", Basic Research in Cardiology, 2022<br>Publication	<1 %
46	advances.sciencemag.org<br>Internet Source	<1 %
47	link.springer.com<br>Internet Source	<1 %
48	www.biorxiv.org<br>Internet Source	<1 %

49	Mesquita, Ângela Sofia Gerós. "Cognitive Perfomance: Exploring the Role of Interleukin-10 in Male Mice", Universidade do Minho (Portugal), 2023<br>Publication	<1 %
50	Shoutang Wang, Raki Sudan, Vincent Peng, Yingyue Zhou et al. "TREM2 drives microglia response to amyloid-β via SYK-dependent and -independent pathways", Cell, 2022<br>Publication	<1 %
51	Simone Brioschi, Wei-Le Wang, Vincent Peng, Meng Wang et al. "Heterogeneity of meningeal B cells reveals a lymphopoietic niche at the CNS borders", Science, 2021<br>Publication	<1 %
52	Colonna-Romano, G.. "A double-negative (IgD^-CD27^-) B cell population is increased in the peripheral blood of elderly people", Mechanisms of Ageing and Development, 200910<br>Publication	<1 %
53	Michael D. Hoos, Brenna M. Richardson, Matthew W. Foster, Angela Everhart, J. Will Thompson, M. Arthur Moseley, Carol A. Colton. "Longitudinal Study of Differential Protein Expression in an Alzheimer's Mouse Model Lacking Inducible Nitric Oxide Synthase", Journal of Proteome Research, 2013<br>Publication	<1 %
54	Peñagaricano, Francisco, Alex H. Souza, Paulo D. Carvalho, Ashley M. Driver, Rocio Gambra, Jenna Kropp, Katherine S. Hackbart, Daniel Luchini, Randy D. Shaver, Milo C. Wiltbank, and Hasan Khatib. "Effect of Maternal Methionine Supplementation on the	<1 %

Transcriptome of Bovine Preimplantation Embryos", PLoS ONE, 2013.
Publication

55	Rui-Yuan Pan, Lin He, Jing Zhang, Xinhua Liu et al. "Positive feedback regulation of microglial glucose metabolism by histone H4 lysine 12 lactylation in Alzheimer’s disease", Cell Metabolism, 2022<br>Publication	<1%
56	Yasuhisa Ano, Rena Ohya, Yuta Takaichi, Terukatsu Washinuma, Kazuyuki Uchida, Akihiko Takashima, Hiroyuki Nakayama. "β-Lactolin, a Whey-Derived Lacto-Tetrapeptide, Prevents Alzheimer’s Disease Pathologies and Cognitive Decline", Journal of Alzheimer's Disease, 2020<br>Publication	<1%

Exclude quotes	Off	Exclude matches	Off
Exclude bibliography	On		
